# Fungal Diversity of Selected Habitat Specific *Cynorkis* Species (Orchidaceae) in the Central Highlands of Madagascar

**DOI:** 10.3390/microorganisms9040792

**Published:** 2021-04-10

**Authors:** Kazutomo Yokoya, Alison S. Jacob, Lawrence W. Zettler, Jonathan P. Kendon, Manoj Menon, Jake Bell, Landy Rajaovelona, Viswambharan Sarasan

**Affiliations:** 1 Royal Botanic Gardens Kew, Richmond, Surrey TW9 3DS, UK; kazyokoya@gmail.com (K.Y.); ali.jacob@virginmedia.com (A.S.J.); J.Kendon@kew.org (J.P.K.); jakebell93@gmail.com (J.B.); L.Rajaovelona@kew.org (L.R.); 2 Department of Biology, Illinois College, Jacksonville, IL 62650-2299, USA; lwzettle@ic.edu; 3 Department of Geography, University of Sheffield, Sheffield S10 2TN, UK; m.menon@sheffield.ac.uk

**Keywords:** Orchidaceae, conservation, global biodiversity hotspot, endemic, orchid mycorrhizal fungus, climate change, *Ceratobasidium*, *Serendipita*, *Tulasnella*

## Abstract

About 90% of *Cynorkis* species are endemic to the biodiversity hotspot of Madagascar. This terrestrial habitat-specific genus received little study for fungal diversity to support conservation. We evaluated the diversity of culturable fungi of 11 species and soil characteristics from six sites spanning a >40 km radius in and along the region’s inselbergs. Peloton-forming fungi were grown in vitro from root/protocorm slices and positively identified using DNA sequencing. The fungal diversity was then correlated with soil pH, NO_3_^-^N, P, and K. All species harbored either putative mycorrhizal associates in the *Rhizoctonia* complex or Hypocreales fungi. *Tulasnella* Operational Taxonomic Units (OTUs) were most prevalent in all soil types while *Serendipita* OTUs were found in species inhabiting granite/rock outcrops in moist soil (seepage areas). Most *Cynorkis* species were present in soil with low NO_3_^-^N and P levels with diversity of mycorrhizal fungi inversely correlated to NO_3_^-^N levels. Of the different orchid life stages sampled, only one species (*Cynorkis fastigiata*) yielded putative mycorrhizal fungi from juvenile stages. As diversity of mycorrhizal fungi of *Cynorkis* spp. was negatively correlated with NO_3_*^-^*N, and majority of the studied taxa were found in soils with low NO_3_^-^N and P contents, reintroduction studies must include analysis of N and P in detail. For the first time, we showed that the assemblage of culturable fungi in the roots of habitat-specific species of *Cynorkis* (Orchidaceae) are intimately tied to specific soil characteristics.

## 1. Introduction

Current estimates project 40% of all plant species will become extinct within 30 years due to anthropogenic factors and climate change [1] and these dire projections have instilled a sense of urgency amongst conservationists to safeguard the species at highest risk. Despite being incredibly diverse with upwards of 25,000 species worldwide [2], orchids are considered highly vulnerable to extinction. According to the International Union for Conservation of Nature (IUCN), half of all orchids that have already succumbed to extinction are herbaceous terrestrials despite comprising only about one-third of all species worldwide [3]. Depending on the species and where it grows, some terrestrial orchids may live for decades (e.g., *Cypripedium calceolus*), whereas others are ephemeral, quickly colonizing an area, setting seed, and perishing shortly thereafter within the span of one year (e.g., *Habenaria repens* [4]). For conservationists faced with the burden of securing orchids under immediate threat, targeting the short-lived terrestrials is a logical priority. To date, those that have received the most study with respect to their biotic and abiotic requirements are rooted in temperate regions, and surprisingly little is known about their tropical counterparts. Considering that most global biodiversity "hotspots" occur in the tropics where 90% of endemic orchids are concentrated, studying these species is of utmost importance. Unfortunately, many of these hotspots are in developing countries that have few monetary resources and, consequently, they remain highly vulnerable to deforestation and other threats (e.g., agricultural practices, mining, political unrest, and poaching). Some of these areas are also remote, making their access by researchers very costly and at times hazardous.

Their vulnerability and success appear to be linked to the close associations they form with other organisms, namely, winged pollinators for sexual reproduction, and mycorrhizal fungi for seed germination and growth through to maturity. These biotic factors therefore transcend all parts of the plant throughout every phase of its life. The orchid, and the organisms they rely on, are also intimately tied to the abiotic factors present in their surroundings such as moisture [5], soil pH [6], sunlight [7], and nutrients [8], all of which may vary considerably along the terrain in each habitat [9,10,11]. As a result, orchids are widespread but limited in where they grow, and they are acutely sensitive to subtle environmental changes once they become established from seed. Conserving these remarkable plants, therefore, poses many challenges that are exacerbated in this age of climate change, and fully understanding these complex interactions is a primary goal.

Despite occupying only 0.04% of the global land surface, the island nation of Madagascar is home to 4% of the world’s biological diversity, and an extraordinary level of endemism among its native flora and fauna [12]. For example, of its estimated 10,000 vascular plant species, 90% are found nowhere else on earth [13] and this is also true of 1000 orchid species within 57 genera [14]. Given that 90% of Madagascar’s original vegetation has been cleared or permanently altered, many orchid populations throughout the country have been reduced in size to fragmented patches where seedling recruitment is generally low [15], possibly due to inbreeding depression [16]. Nevertheless, these forest remnants still support a surprisingly diverse orchid flora. A recent study revealed that Malagasy inselbergs (isolated mountains of granitic or gneissic origin) in the Central Highlands accommodated 87 orchid species despite frequent disturbances present in the region (e.g., annual burning, mining, cattle raising) [17]. They also revealed that *Cynorkis* is the most abundant genus in the region (18 species) whose members are typically short-lived, appealing terrestrial herbs that form a dense vegetative layer over shallow, nutrient-poor substrates. Collectively, these species persist in “micro-hotspots” or even smaller patches termed “nano-hotspots” where exceptional endemism may be concentrated in areas < 3 km^2^ [18]. The scale of diversity within these small hotspots offers a unique opportunity to study the fungal associates of these orchids, and by doing so these plants could potentially be propagated artificially from seed and reintroduced, augmenting their long-term conservation.

The genus *Cynorkis* encompasses 170 species, with 120 found in Madagascar alone [19], and the vast majority of which remain unstudied with respect to their mycorrhizal associations. Yokoya et al. [20], however, revealed that one species (*Cynorkis purpurea*), native to stream beds in the Central Highlands, harbored a surprisingly rich assemblage of *Rhizoctonia*-like fungi that typically form mycorrhizal associations (*Ceratobasidium*, *Serendipita*, *Tulasnella*). When seeds of *C. purpurea* were later inoculated with these fungi in vitro, the mycorrhizal nature of the fungi was confirmed [21] suggesting that this orchid may be a “fungal generalist” [1]. The success of these two studies served as the impetus for further work involving other *Cynorkis* species in the region.

The aim of this paper is to characterize the fungal diversity associated with at least 50% of *Cynorkis* species found in the micro-hotspot of the Central Highlands in Madagascar, including their mycorrhizal associates as well as non-mycorrhizal (saprotrophic) inhabitants. In addition, we attempt to link fungal diversity to various abiotic soil factors (e.g., pH, N, P, and K nutrient levels) at various geographic locations to garner a broader picture of how these orchids function and persist in nature. To our knowledge this is the first study of its kind for an orchid genus from Madagascar. Our goal is to provide more information for a poorly understood but well-known orchid genus in one of the most remote, highly vulnerable, and biologically significant regions on earth.

## 2. Materials and Methods

### 2.1. Study Area and Collection of Samples

**Figure 1:**
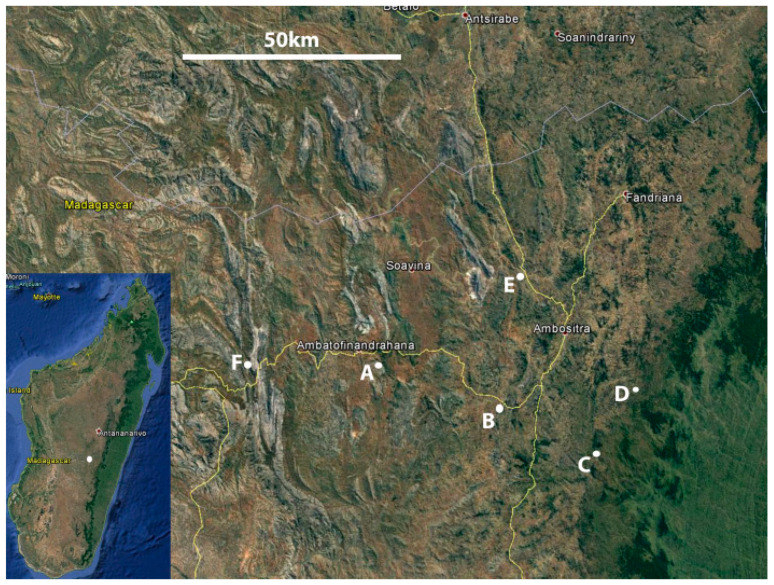
Location of study site in the Central Highlands of Madagascar (inset) and collection sites A–F (main). Scale bar= 50 km. Image courtesy of Google Maps.

**Figure 2:**
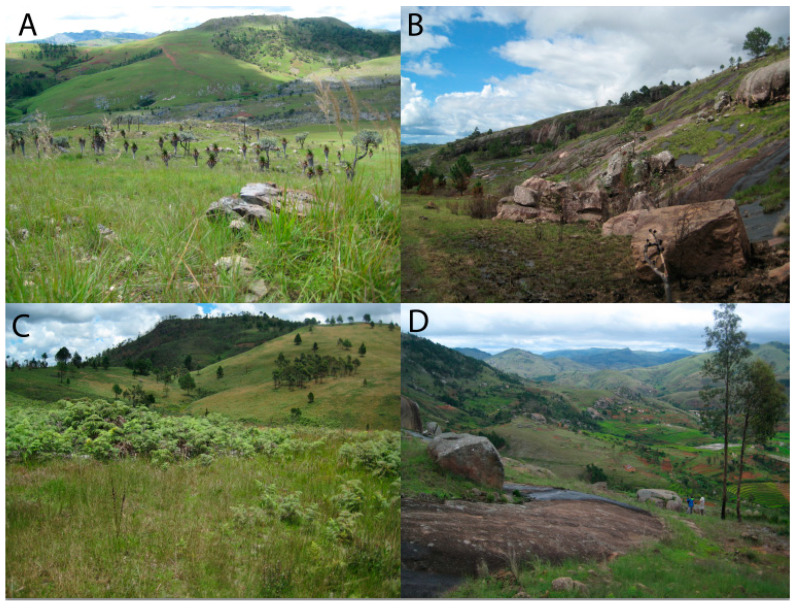
Topography of four of the collection sites; (**A**) Mountainous, outcrops of marble and quartzite, (**B**) Base of wide valley with granite slabs and large, fast-flowing river, (**C**) Rolling hills with few granite outcrops, (**D**) Steep hills with boulders.

Six collection sites A–F (Figure 1 and Figure 2; Table 1) were selected with a 40 km radius to collect the samples. The sites were selected based on their distinctive habitats, past records of *Cynorkis* occurrence, high local diversity of orchids and relative accessibility. All sites were subjected to typical weather patterns for the Central Highlands of Madagascar, namely pronounced wet (January to April) and dry (May to September) seasons. This study took place at the beginning of the rainy season. Figure 2 shows the typical topography of four selected sites.

Import and export permits, including CITES (the Convention on International Trade in Endangered Species of Wild Fauna and Flora), were secured for this purpose, and are described in detail in Zettler et al. [22]. Out of 18 species of *Cynorkis* known to occur in the Itremo Massif of Central Madagascar [14], permits were obtained for 14 species, 11 of which were collected during two separate trips during the dry and rainy seasons, respectively. Government restrictions were imposed on the number of roots that were collected per species (3), limiting the amount of material available for export thereby reducing our sample sizes.

**Figure 3:**
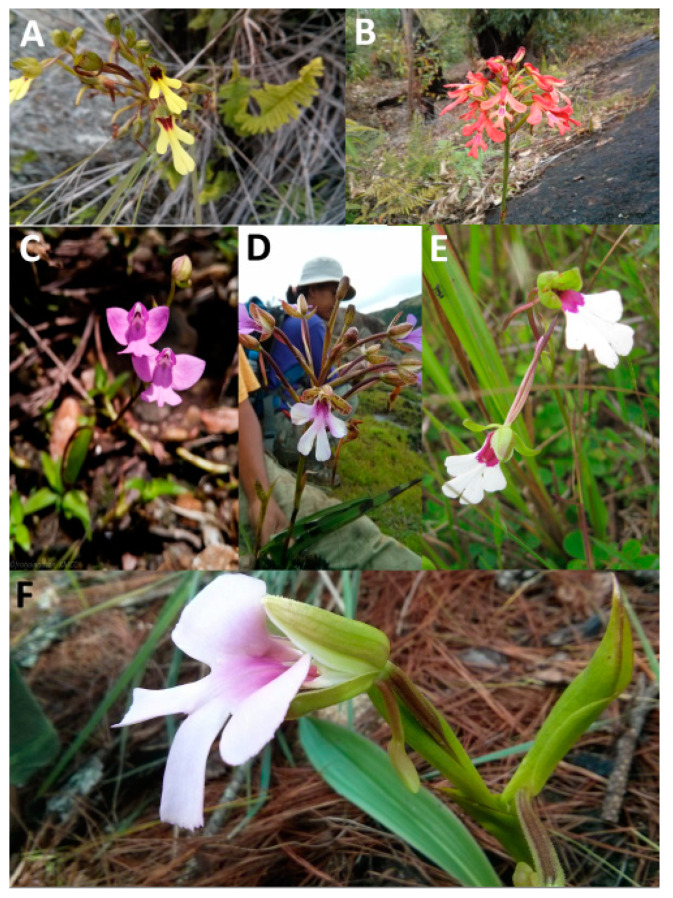
Some of the *Cynorkis* species from which fungi were isolated. (**A**) *Cynorkis flexuosa*, (**B**) *C. gibbosa*, (**C**) *C. purpurea*, (**D**) *C.* × *ranaivosonii*, (**E**) *C. speciosa*, (**F**) *C. uniflora*.

Except seedlings of *C. purpurea,* which were collected during dry season, all other taxa were collected in 2015 immediately following the rainy season (Figure 3 showing some of the taxa collected). These taxa were studied for mycorrhizal and non-mycorrhizal fungi; however, *C. purpurea* was studied just for mycorrhizal fungi. Therefore, this species is treated separately for the purpose fungal diversity analysis. Samples of protocorms, seedlings and mature-phase plants were sourced from more than one population in many cases and screened for peloton-forming fungi.

Due to the 2–3-week delay between field collection and fungal isolation in the UK and USA, roots remained in contact with soil until the day before export. Upon arrival at the Kew’s Madagascar Conservation Center (KMCC) base in Antananarivo, 2–7 days after field collection, root samples and root balls were chilled at ca. 6 °C in a refrigerator. Approximately 24 h before departure from the country, roots of *Cynorkis* spp. were removed from soil and rinsed off with UV-irradiated water to remove organic debris to comply with US and UK import regulations. Lateral branch roots were detached and placed over a pre-moistened cotton ball in a pre-sterilized 8 mL glass vial and sealed with Parafilm^®^ M. Roots were transported as carryon baggage by air to minimize exposure of the samples to temperature extremes [22]. In addition to live material, soil samples (organic material at various stages of decomposition mixed with soils/sediments) were collected in the immediate (1 m) vicinity from all target species studied. As with the export of living orchid material, soil samples were also restricted requiring a collecting permit issued by the Madagascan authorities. These restrictions allowed for a single soil sample to be exported with each of plant samples collected. Although more samples would have been preferable for our soil data analysis, the existing samples were of sufficient quantity. Soil samples were analyzed using LaMotte STH Series Combination Soil Testing Outfit (LaMotte, Chestertown, MD, USA) to obtain pH, NO_3_^-^N, P, and K. For each soil component, the appropriate test reagents were added to soil sample extracts according to the Testing Outfit manual instructions. Following each reaction, soil extract solution colors and turbidity were compared to the test cards to ascertain the amount present. Soil moisture content was rated based on an arbitrary scoring system i.e., (1) dry = soil feels dry to the touch; (2) moist = soil feels moist to the touch; (3) wet = soil water-logged/saturated.

### 2.2. Fungal Isolation, Initial Identification, and Deposition

Processing of root samples lasted 10 days during which time fungal isolations took place. Immediately upon arrival, root samples were placed in refrigeration (4–6 °C) at Royal Botanical Gardens, Kew, United Kingdom and Illinois, USA from Madagascar. Mycorrhizal fungi were isolated following the method of Zettler et al. [22]. Once the screening and culturing completed some pelotons failed to grow and produce viable colonies of fungi in culture. On average more than six roots were processed with each root yielded >10 slices and >200 pelotons. Wherever individual pelotons were difficult to isolate and cultured individually additional clumps of macerated cortical cells containing pelotons were also isolated. Pelotons and cortical cells containing pelotons were immersed in Fungal Isolation Medium (FIM) [23] containing streptomycin sulphate [24] and incubated at 18 °C. After 1–4 days, hyphal tips that were observed emerging from pelotons under a dissection microscope were subcultured to FIM (Kew) or Potato Dextrose Agar (PDA, Difco™, Becton, Dickinson and Co., Sparks, MD, USA) using a sterile scalpel. Potential mycorrhizal fungi (MF) were initially distinguished from common sporulating fungi using previously published descriptions [25]. Those that displayed morphological characteristics of *Rhizoctonia*-complex (e.g., Tulasnellaceae, Ceratobasidiaceae), such as monolioid cells, were retained for further identification by ribosomal DNA internal transcribed spacer (rDNA ITS) amplification and sequencing. To safeguard the mycorrhizal OTUs (Operational Taxonomic Units) for the purposes of future work (e.g., symbiotic seed germination) and long-term conservation, they were stored in cryopreservation in vapor phase as described by Schofield et al. [26].

### 2.3. Molecular Identification of Fungi by ITS Sequencing

Cultures that were provisionally identified as potential mycorrhizal fungi were retained for further study. Pure cultures of fungi were positively identified using DNA sequencing as described in Yokoya et al. [20]. Briefly, DNA from fresh mycelia was extracted in 96-well plates using the Extract-N-Amp™ Plant Tissue PCR Kit (Sigma Aldrich, Gillingham, UK). PCR amplification of the ITS region using primer ITS1F with ITS4 and ITS1 with ITS4-tul [27,28,29] was followed by Sanger sequencing using the same forward and reverse primers. The forward and reverse sequences were checked for accuracy and consensus and compared with database sequences using BLAST (National Center for Biotechnology Information, Bethesda, MD, USA). Sequences that matched *Rhizoctonia*-like fungi were aligned and grouped into OTUs based on a conservative similarity threshold of 95%. Representative sequences of each OTU were used to re-query the GenBank database using BLAST and deposited in GenBank. Orchid mycorrhizal fungal isolates were cryopreserved at RBG Kew for use in symbiotic seed germination trials and use in conservation.

### 2.4. Phylogenetic Characterization of *Cynorkis* spp.

The DNA of root materials was extracted to sequence the chloroplast DNA region trnL-F. DNA was extracted using a modified CTAB (cetyltrimethylammonium bromide) protocol [30] followed by chloroform/isoamyl alcohol (24:1) extraction and precipitation in isopropanol. The trnL-F sequences were amplified using primer combinations c with d for the trnL intron and e with f for the trnL-F intergenic spacer [31] as described previously [20]. The PCR products were cleaned using QIAquick^®^ columns (Qiagen Inc., East Crawley, UK) and sequenced as described above for fungus ITS sequencing. Sequences were aligned and phylogenetic trees were created using MEGA X: Molecular Evolutionary Genetics Analysis across computing platforms [32].

### 2.5. Species Richness and Diversity

The aim of this analysis was to see whether *Cynorkis* spp. (except *C. purpurea*) showed any trend on fungal diversity based on soil characteristics. Species richness of root-associated fungi was calculated as the sum of all fungal OTUs identified by ITS sequencing for an individual plant (with biological replicates averaged where present). Species diversity was calculated with the Shannon diversity index (H) [33] and the Simpson diversity index (D) [34]. *H* measures the richness and proportion of a given species within a community, whilst D measures the probability of two randomly selected individuals (OTUs) belonging to the same species [35].

H was calculated using the following equation:

$$H=-\overset{S}{\underset{j=1}{\sum }}p_{i}\ln\;p_{i}$$

D was calculated using the following equation:

$$H=\;\frac{1}{\sum_{j=1}^{S}p_{i}²}$$

For both H and D, S represents the total number of species in the community (richness), and pi represents the proportion of species richness made up of an individual species. Both and H and D were calculated separately for mycorrhizal and non-mycorrhizal fungi as well as when they were combined.

### 2.6. Statistical Analysis

We used GraphPad Prism (v 9) to generate heatmaps of Pearson correlation (r) between the environmental factors (pH, N, K and Elevation), diversity indices (H and H).

## 3. Results

Collecting orchid and soil samples from the Central Highlands of Madagascar for the current study was challenging due to problems with access, number of days required to screen and culture in vitro over a three-week period. The number of roots that yielded pelotons was relatively low (Supplementary Materials File S1). A smart kit is the answer to collect and culture pelotons within couple of days of collecting which will help recover all peloton-forming culturable fungi collected from the wild. Although, in many species, more than two plants were sampled recovery of *Rhizoctonia* OTUs was significantly low. Except in *Cynorkis fastigiata*, which yielded tul5, all *Rhizoctonia* OTUs were identified from roots of mature plants.

### 3.1. Distribution of Mycorrhizal and Non-Mycorrhizal Fungi

**Figure 4:**
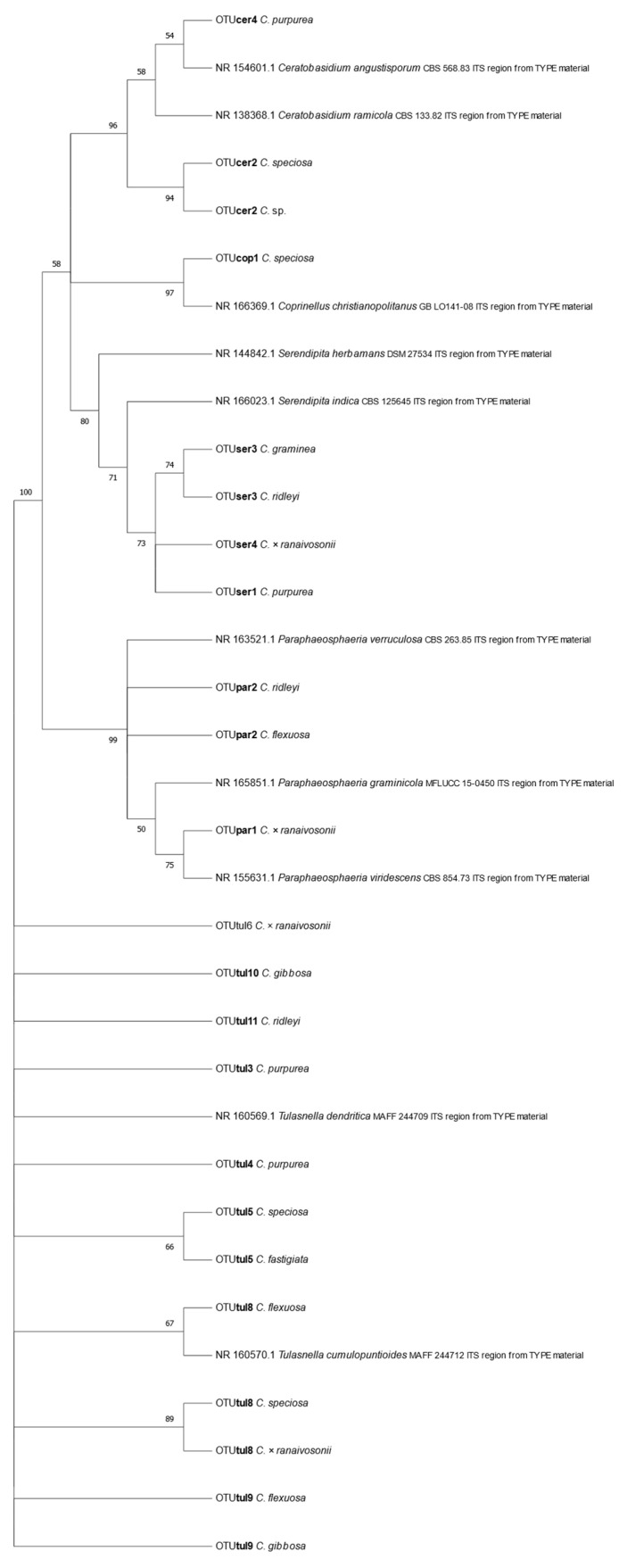
Phylogenetic tree of putative *Rhizoctonia* mycorrhizal fungi (cer—*Ceratobasidium*, ser—*Serendipita,* and tul—*Tulasnella*), non-*Rhizoctonia* fungi such as *Coprinellus* and *Paraphaeosphaeria* and their hosts.

Culturable mycorrhizal fungi were obtained from nine of the 11 Cynorkis species: *Cynorkis flexuosa*, *Cynorkis fastigiata*, *Cynorkis gibbosa*, *Cynorkis graminea*, *C. purpurea*, *C.* × *ranaivosonii*, *Cynorkis ridleyi*, *Cynorkis speciosa.* and *Cynorkis* sp.) while two species (*Cynorkis papillosa* and *Cynorkis uniflora*) failed to yield fungal endophytes. Cantharellales (*Ceratobasidium* and *Tulasnella*) fungi were successfully isolated from eight out of 11 species while members of Serendipita (*Sebacinales*) were recovered in four species (Figure 4). Except *C. papillosa* and *C. uniflora*, all other species had mycorrhizal OTUs in their root cortical cells. 

Of the fungi obtained, mycorrhizal fungal OTUs from the genera *Ceratobasidium*, *Serendipita,* and *Tulasnella* were identified in addition to orchid mycorrhizae such as *Coprinellus* and *Paraphaeosphaeria*. In total two *Ceratobasidium*, three *Serendipita* and eight *Tulasnella* OTUs were associated with nine of the *Cynorkis* species. OTU tul8 was found in three *Cynorkis* species, OTUs tul5 and tul9 in two species while the remainder were recorded only once in the genus. Culturable mycorrhizal OTUs were distributed in varied numbers with four in *C. purpurea* while *C.* × *ranaivosonii* and *C. speciosa* had three OTUs. Only a single OTU each were recovered from *C. graminea* and *Cynorkis* sp.

In total there were13 *Rhizoctonia* OTUs identified from nine of the 11 *Cynorkis* species of which eight were *Tulasnella*, three were *Serendipita* and two were *Ceratobasidium* (Figure 4). *Cynorkis purpurea* and *C.* × *ranaivosonii* hosted four OTUs followed by, *C. flexuosa*, *C. ridleyi* and *C. speciosa* (three each). OTUs tul8, tul9, cer2 seb3 were the only OTUs found in two *Cynorkis* spp. *C. fastigiata* and *C. graminea* had a single OTU each. Non-Rhizoctonial *Paraphaeosphaeria* was identified from three taxa (*C. flexuosa*, *C.* × *ranaivosonii* and *C. ridleyi*).

### 3.2. Phylogenetic Characterization of *Cynorkis* spp.

The primary aim of genotyping the host plants was to confirm the taxonomic status of protocorms, seedlings and plants without flowers from which root materials were collected. All sequenced juvenile samples matched those of the expected *Cynorkis* species following sequencing of the chloroplast DNA region trnL-F. Phylogenetic analysis showed that *C. graminea* and *C. ridleyi* were of the same clade and they were also closely related to *C. papillosa* and *Cynorkis* sp., all of which consist of relatively small plants with small flowers.

### 3.3. Fungal Diversity Shannon’s Index and (H) and Simpson’s Index (D) of *Cynorkis* spp.

**Figure 5:**
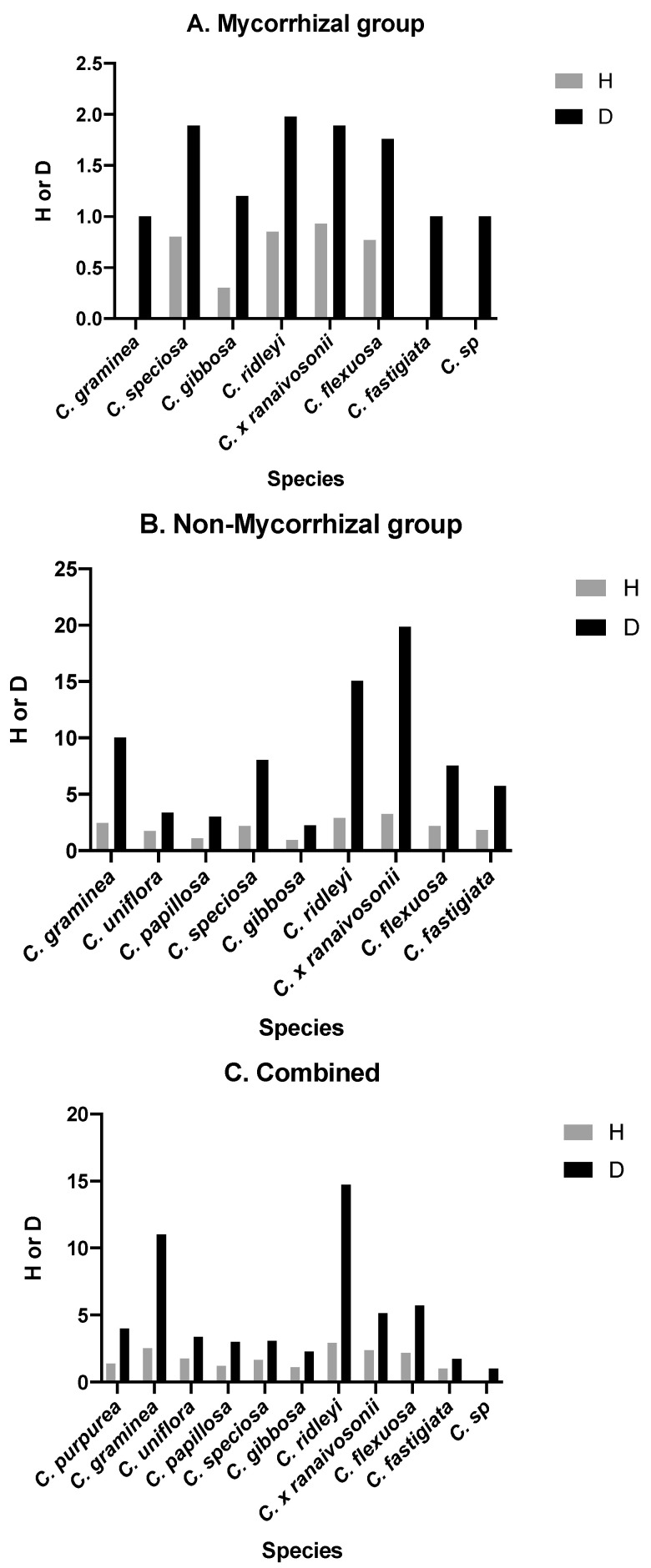
Diversity indices, Shannon’s (H) and Simpson’s (D), of mycorrhizal fungi (**A**), non-mycorrhizal fungi (**B**) and Total (**C**) of eleven *Cynorkis* species (non-mycorrhizal values not counted for *C. purpurea*).

Figure 5A–C shows H and D for mycorrhizal (A), non-mycorrhizal (B) and combined (C) groups). For mycorrhizal group (Figure 5A), H and D were higher for four species namely *C. speciosa, C. purpurea*, *C.* × *ranaivosonii and C. ridleyi* in comparison to others whereas the latter two were amongst the top in H and D for the non-mycorrhizal group (Figure 5B). When mycorrhizal and non-mycorrhizal groups were combined, H and D showed slightly different trend as *C. ridleyi* > *C. graminea*> *C.* × *ranaivosonii.*

Our analyses revealed the diversity values for different *Cynrokis* spp. For example, *C.* × *ranaivosonii* followed by *C. ridleyi* from wet habitats (arb. value 3), had the highest diversity index values (Supplementary Materials File S2). However, the combined diversity index of 10 species (excluding *C. purpurea*) shows *C. ridleyi* harbored the most fungal diversity (only two mycorrhizal fungi identified- *Tulasnella* and *Serendipita*) followed by *C. graminea* which had *Serendipita* as the lone mycorrhizal fungus. Combined analysis for diversity using Shannon and Simpson’s shows *C. ridleyi* harboring the most diverse assemblage of fungi identified from its roots.

*C. purpurea* followed by *C.* × *ranaivosonii* from wet habitats (arb. value 3) had the highest diversity for mycorrhizal fungi (as confirmed by Shannon diversity analysis in Figure 5) while *C. ridleyi* came second in diversity index according to Simpson’s index.

### 3.4. Relationships between Abiotic Factors and the Fungal Diversity

Except for *C. purpurea*, the link between these *Cynorkis* species, fungal diversity (H) and (D) (mycorrhizal and non-mycorrhizal), and soil characteristics is a mixed one. For example, the *Cynorkis* species documented in this study were found growing in seasonally wet, free-draining soil, somewhat peaty and high in organic matter at varying stages of decomposition. Very little loam, sand, silt, or other sediments were observed. Soil sample analysis revealed that all *Cynorkis* species inhabited soils that were nutritionally poor and slightly acidic. Average values (for 102 mycorrhizal and non-mycorrhizal OTUs) were in the lower scale for respective minerals. NO_3_^-^N (24 ppm, 10 ppm lowest value), P (18.4 ppm, 5 ppm lowest value), K (110 ppm, 50 ppm lowest value), and average pH was 5.2. Our observations showed that out of the 11 species studied, three (*Cynorkis* sp., *C. fastigiata*, *C*. *flexuosa*) were growing in a moist soil environment (arbitrary value of 2) while the others were associated with a wetter (value 3) soil.

**Figure 6:**
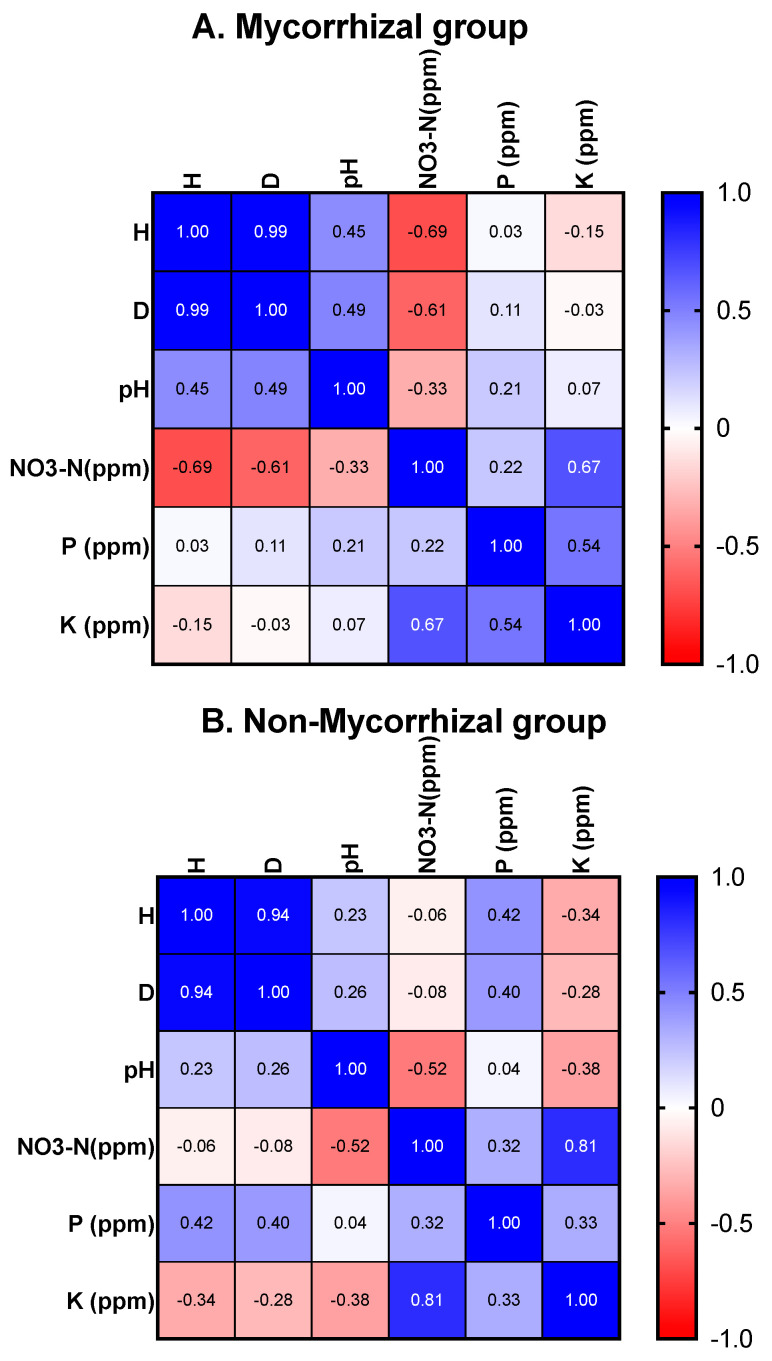

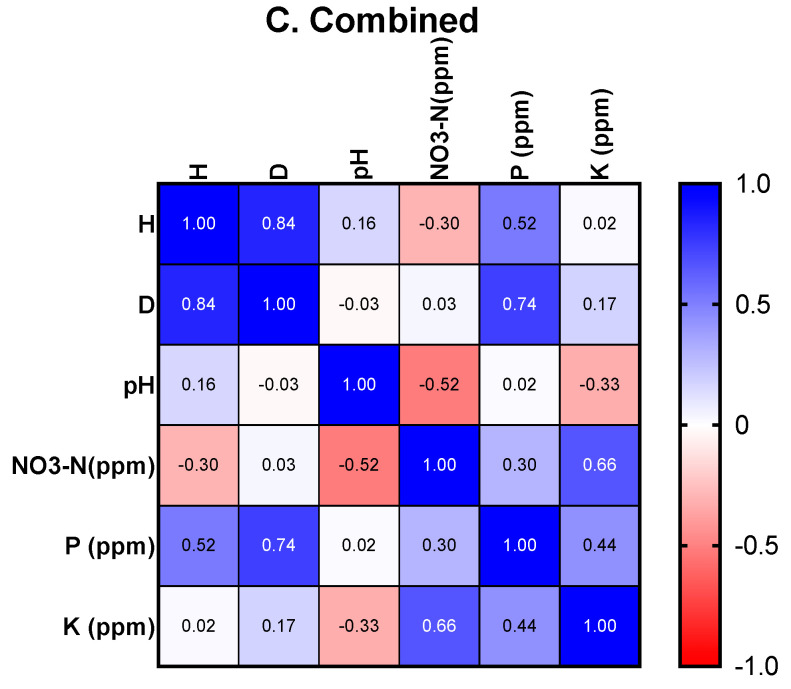
(**A**–**C**) Pearson correlation heatmaps showing the relationship between diversity indices (Shannon’s— H, Simpson’s— D) and soil parameters (pH, NO_3_^-^N, P, and K).

Based on the measured soil properties (pH, NO_3_^-^N, P, and K) and calculated diversity indices (H and D), Pearson correlation heatmaps are depicted in Figure 6 for mycorrhizal (Figure 6A), non-mycorrhizal (Figure 6B) and combined (Figure 6C) for all taxa of except *C. purpurea*. Note that *p* values are provided in the Supplementary Materials File S2.

**Figure 7:**
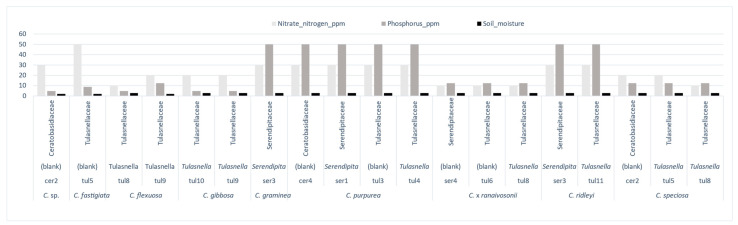
Distribution of putative mycorrhizal Operational Taxonomic Units (OTUs) (families and genera) in 11 species of *Cynorkis* at two different moisture levels, nitrogen, and phosphorus content (ppm).

*Tulasnella* OTUs were found in all soil types while *Serendipita* OTUs were found in species inhabiting in granite/rock areas close to river/seepage. Diversity of mycorrhizal OTUs was comparably higher in wet habitat as shown in Figure 7 (arbitrary score of 3). *C. flexuosa* (tul8 and tul9) *C.* × *ranaivosonii* (ser4, tul6, and tul8), *C. speciosa* (cer2, tul5, and tul8) and *C. gibbosa* (tul9, tul10) were from soil with low NO_3_^-^N and P content. These orchids recruited either tul8 or tul9, two closely related OTUs (Figure 4). Of these four species tul8 was present in all but *C. gibbosa* which recruited tul9. This means low P and N favored orchids to recruit a selection of *Tulasnella* OTUs and small number of *Serendipita* and *Ceratobasidium* OTUs.

On the contrary, *C. graminea*, *C. purpurea* and *C. ridleyi* were the only three species found in N and P rich soils, P (50 ppm) and N (30 ppm). Under same soil conditions *C. graminea* and *C. ridleyi* recruited a single *Serandipita* OTU and an additional tul11 by *C. ridleyi*. Both were from similar moisture conditions. In *C. ridleyi* a diminutive species putative mycorrhizal OTUs ser3 and tul11 were identified from plants collected from soil with 50 ppm of *p* while plants from soil with 9 ppm of P had Hypocreales and Helotiales fungi in addition to other non-mycorrhizal fungi (Figure 7).

When all *Cynorkis* spp. were analyzed (except *C. purpurea*) NO_3_^-^N, pH, P, and K are found to be correlated with diversity of fungi (H and D). Specifically, mycorrhizal diversity is inversely correlated to NO_3_^-^N in *Cynorkis* spp. (Figure 6A). However, pH is positively correlated with both diversity indices (the difference between pH values were miniscule though). This revealed a positive correlation between higher pH values and higher orchid diversity and abundance. When different taxa were analyzed separately most of the orchids studied favored lower nitrogen and P levels (Figure 7).

P is positively correlated with diversity (H and D) of non-mycorrhizal for all *Cynorkis* taxa (except *C. purpurea*). This indicates that more non-mycorrhizal fungi were recruited when P was comparatively high. K content was negatively correlated with diversity (H) of non-mycorrhizal fungi (Figure 6B). Likewise, P is positively and significantly (*p* = 0.009) correlated with D when mycorrhizal and non-mycorrhizal fungi are combined (Figure 6C).

**Figure 8:**
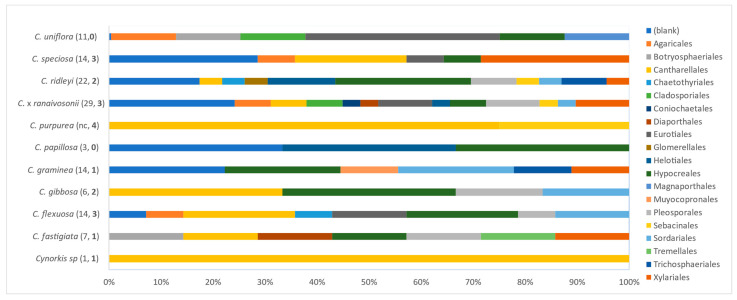
Proportion of mycorrhizal and non-mycorrhizal orders in 11 species of *Cynorkis* (total number OTUs and mycorrhizal OTUs (in bold) in brackets, nc—non-mycorrhizal fungi not counted for *C. purpurea*).

*Paraphaeosphaeria* OTUs, a group of putative mycorrhizal fungi, were detected in *C. flexuosa*, *C.* × *ranaivosonii*, and *C. ridleyi* while *Coprinellus* was found in *C. speciosa*. *Cynorkis* × *ranaivosonii* followed by *C. ridleyi*, then *C. graminea* had the highest non-mycorrhizal fungal diversity (as shown by Shannon and Simpson’s diversity analyses (Figure 6B and Figure 8). Data on non-mycorrhizal fungi of *C. purpurea* were missing because this species was not collected and included for further analysis. Hence, results on *C. purpurea* are incomplete.

The fungal diversity in 11 species of *Cynorkis* was statistically significant based on P content in the soil. However, data were not significant when calculated separately for mycorrhizla and non-mycorrhizal fungi. Hypocreales, an order characterized by having dark-septate hyphae, were recruited invariably by these orchids when *Rhizoctonia* lineages were absent.

Two smallest herbaceous orchids, *C. graminea* and *C. ridleyi*, growing in arb wet = 3, harbored the most non-mycorrhizal fungi, second only to *C.* × *ranaivosonii* (Figure 8). Members of the Hypocreales colonized nine out of 10 species assessed (*C. purpurea* excluded). Fungi assignable to the Eurotiales and Xylariales were secondary inhabitants, found in five species each. Members of the Eurotiales, Pleosporales, Sordariales, and Xylariales were present in nearly 50% of *Cynorkis* species (Figure 8) suggesting that a variety of dark-septate endophytes colonize the roots of this genus. Interestingly, fungi assignable to Pleosporales and Xylariales were found only in roots that were also colonized with mycorrhizal fungi. Further, worth noting were the presence of Psathyrellaceae OTUs (Agaricales comprises ectomycorrhizal fungi such as *Coprinellus*) detected in four *Cynorkis* species. Only a few OTUs were recovered from *Cynorkis* sp. and *C. papillosa* due to deterioration of root samples over the three week period.

## 4. Discussion

The Central Highlands of Madagascar is characterized by tough terrain that severely restricts access to orchid populations found clustered along the region’s inselbergs in pockets where moisture collects and only accessible by foot. Restrictions by the Madagascan authorities on sample collecting also limited the number of root/protocorms that we were able to collect. Despite these hardships, we were able to obtain enough samples to provide a snapshot of *Cynorkis* diversity in the Central Highlands linked to soil, moisture, nutrients, and fungal diversity for a respectable number of species.

About 10% of all angiosperm species are orchids which are associated with roughly 25,000 Basidiomycota and some Ascomycota as fungal partners [36,37]. These associations are guided by soil characteristics, seasonality, and life forms of the orchids [9,10,11].

To our knowledge, this is the first study that correlated soil nutrients, moisture, and fungal diversity in a well-known group of terrestrial orchids (*Cynorkis* spp.), the majority of which are endemic to Madagascar. These results could have a long-term impact on the conservation of species native to central Madagascar especially for reintroduction efforts being planned that will require navigating hardships imposed by climate change.

Most of the 11 *Cynorkis* species studied herein occupied two primary habitats—gallery forests and grasslands. Historically these species would have been abundantly distributed in wet soil (arb value 3) but may have adapted to less wet (arb value 2) soil due to deforestation and ongoing climate change. In a climate change scenario of erratic rainfall and drought, it is plausible that these *Cynorkis* species will face increasing challenges in survival and natural regeneration. Accordingly, mycorrhizal diversity needed by these orchids for seed germination and survival must be maintained to reduce population decline in these unique habitats that support the majority of *Cynorkis* spp. Even though some *Cynorkis* spp. appear to be resilient in moist (arb 2) areas, further environmental changes that permanently alter these habitats by stripping critically needed moisture may impede the long-term survival of these species. The results of a previous in vitro symbiotic seed germination study by Rafter et al. [21] demonstrated that *C. purpurea* is a generalist. Most OTUs were identified from *C. purpurea* in this study which may also suggest the generalist nature. Nurfadilah et al. [38] demonstrated that different soil N forms (ammonium and nitrate) can be exploited by generalists possibly because the co-occurrence of mycorrhizal fungi enables the plants to utilize a wider range of nutrient sources.

Ephemeral flush vegetation as reported by Rabarimanarivo et al. [17] has a meadow-like physiognomy and display seasonality between rainy and dry season known to support genera such as *Cynorkis*. Most members of this genus occupy gently inclined rocky slopes where water constantly seeps during the rainy season and in some cases even during the dry season. Although *Cynorkis* is distributed in many other parts of Madagascar, the majority are found in wetter habitats which make them vulnerable to population decline in the absence of optimal moisture levels. Annual wet season rains may represent a key factor in generating spontaneous seedlings, and that if this cycle is broken in successive years, some *Cynorkis* taxa will likely suffer from population decline. As this biodiversity hotspot has experienced extensive deforestation and fragmentation, climate change is expected to exacerbate species loss in vulnerable habitats in the decades to come [39].

Out of 11 species, *C. speciosa*, *C. purpurea*, *C*. × *ranaivosonii,* and *C*. *ridleyi* had highest biodiversity indices for mycorrhizal fungi. When mycorrhizal and non-mycorrhizal groups were combined, H and D showed slightly different trend as *C. ridleyi* > *C. graminea* with the highest indices. Combined analysis for diversity using Shannon and Simpson’s shows *C. ridleyi* harboring the most diverse assemblage of fungi identified from its roots. The small-flowered *C. graminea* and *C. ridleyi*, inhabiting close to fast flowing river from lithophytic habitat hosted the greatest number of fungi. This may be because these species undergo more environmental fluxes than other orchids which would drive them to recruit a diverse fungal assemblage for their resilience in the habitat.

Diversity (H and D) of *Cynorkis* spp. were correlated with NO_3_^-^N, pH, P, and K. The general picture is showing less nitrogen favored greater mycorrhizal diversity. However, when each species from unique habitats and soil types were assessed separately, they showed low P in addition to NO_3_^-^N favored distribution of *Cynorkis* spp. and the assemblage of mycorrhizal fungi. Therefore, for the purpose of understanding specific requirements for reintroduction, targeted environmental analysis is required. Previous studies indicate that low P content may be linked to higher mycorrhizal diversity in *Cephalanthera rubra* [40] and several orchids from Madagascar [11]. Likewise, Van der Ent et al. [41] found that the rhizosphere soil concentrations of P and K was low in soil in the study site where *Paphiopedilum rothschildianum* was growing. Figura et al. [42], based on in vitro seed germination studies, found that nitrates negatively affect symbiotic seed germination. The current study showed less NO_3_^-^N and higher P favored greater mycorrhizal diversity.

*Cynorkis* spp. had high diversity (H and D indices) for non-mycorrhizal fungi when P content was high and K content was low. As Bell et al. [40] reported with *Cephalanthera rubra*, members of *Cynorkis* also recruited more non-mycorrhizal fungi when soil P values were comparatively high. The Central Highlands landscape is prone to eutrophication due to burning and grazing. Nitrate leeching into soil and finding their way into streams where *Cynorkis* are rooted is a real possibility which may adversely affect seedling recruitment. A more detailed analysis, with soil samples from wider population ranges, will be required to demonstrate the likelihood that this may occur.

In *C. ridleyi*, plants collected from soil with 50 ppm of P had two putative mycorrhizal OTUs, ser3 and tul11, while plants from soil with 9 ppm of P had non-mycorrhizal fungi like Hyopcreales and Helotiales. The distribution of soil fungi is related to spatial variation and soil chemistry [43,44,45], the suite of fungi the orchid hosts is correlated to the soil environment, indicating how soil chemistry determines orchid fungal associations across landscapes. However, Waud et al. [46] noted that while mycorrhizal specificity is not critical for the distribution of an endangered mycorrhizal generalist orchid (*Liparis loeselii*), germination was affected by soil moisture content. Thus, the distribution of rare orchid species may depend on a complex interaction between soil parameters and the soil mycobiota. Further studies to understand orchid recruitment are needed to reach conclusions on the causes of rarity of *Cynorkis* orchids.

We studied 11 *Cynorkis* species of which nine yielded 13 mycorrhizal fungi. While eight had *Tulasnella* OTUs, *Serendipita* was only observed in three species suggesting that *Serendipita* is more limited in distribution in granite/rocky habitats. These fungi when available, it may have considerable potential at generating seedlings. Rafter et al. [21] revealed that in vitro seedling development in *C. purpurea* was supported by a wide suite of fungi but was most pronounced with *Serendipita* OTUs. The phylogenetic tree of mycorrhizal fungi shows the *Serendipita* fungal OTUs are associated with phylogenetically close *C. graminea*, and *C. ridleyi*. This observation is important, and requires further study linking phylogeny of orchid species, mycorrhizal fungi, and their habitats, in this case granite outcrops which has a thin layer of topsoil.

Ascomycetous *Paraphaeosphaeria* was also present in *C. flexuosa*, *C.* × *ranaivosonii*, and *C. ridleyi* while *Coprinellus* was found in *C. speciosa.* These species may be of generalist nature or we may incidentally have collected the healthiest samples. Molecular data in a forage grass revealed that the root mycobiota was dominated by *Paraphaeosphaeria* spp. which was clearly tied to water amendments and the addition of similar volumes of water [47]. They also suggested that *Paraphaeosphaeria* was involved with a common but complicated strategy of water translocation in a variety of soil types, like that shown in the arbuscular mycorrhizal fungi. Whether or not this strategy is also employed in *Cynorkis* is not known but warrants further study.

Yagame et al. [48] revealed for the first time that members of the basidiomycete genus *Coprinellus* (Psathyrellaceae) have the potential to serve as orchid symbionts capable of facilitating seed germination in a photosynthetic orchid. In a subsequent study by Shao et al. [49], OTUs assignable to *Coprinellus* were present in protocorms of several epiphytic *Dendrobium* species, although in their germination trials, these *Coprinellus* isolates failed to stimulate symbiotic germination in vitro. Yokoya et al. [11] reported *Coprinellus* sp. was identified in *Liparis ochracea* collected from Madagascar.

Four orchid taxa recruited Helotiales in this study, and these fungi were included as putative mycorrhizal associates in studies pertaining to fungal diversity in *Epipactis* spp. and *Dactylorhiza* spp. [37,50]. They found that, in *Epipactis* spp., Helotiales was the most abundant alleged mycorrhizal fungal group. For *Paphiopedilum spicerianum* inhabiting natural habitats, the most significant factors for fungal communities were hydrolysableN, totalP, and water content [51], where it was found that fungal community change could be related to nutrient requirements and plant phenology.

*Serendipita* isolates from *C. graminea* and *C. ridleyi* were of the same OTU (OTUser3) that was previously found to support seedling growth of *C. purpurea* [21]. These two species are phylogenetically closely related and, like *C. purpurea*, are relatively diminutive among the *Cynorkis* species that were seen growing at the margins of small rivers. These species may have retained juvenile characteristics that predispose them to continue their association with fungal symbionts from seedling stage. OTU ser3 may be one of the favored OMFs of *Cynorkis* species, especially for juvenile stages supporting results of the in vitro symbiosis seen in *C. purpurea* [21]. Studies by Djordjević et al. [9] showed that among other variables soil moisture, altitude, and soil pH were the main factors affecting the distribution and abundance of orchid species in grasslands and herbaceous wetlands in western Serbia. As herbaceous terrestrial orchids are more vulnerable to extinction compared to other orchids, species like *C. graminea* and *C. ridleyi* found in riverbanks may be vulnerable to population decline due to climate change related moisture imbalances.

## 5. Conclusions

All species studied had either mycorrhizal fungi or Hypocreales fungi. Only one juvenile sample yielded a *Rhizoctonia* OTU (from a protocorm of *C*. *fastigiata*) which is a striking finding. *Tulasnella* fungi were identified from different soil types while *Serendipita* were only from *Cynorkis* spp. from rocky/granite close to rivers or have water seepages. Phylogenetically closely related, two small-flowered species, *C. graminea* and *C. ridleyi,* hosted the same *Serendipita* OTU. They were from the banks of a river in a lithophytic habitat which calls for further studies to understand these associations and its influence on species resilience.

Diversity of mycorrhizal fungi were negatively correlated with NO_3_^-^N while higher P content favored greater non-mycorrhizal diversity (H and D indices). However, when different species from unique habitats were assessed separately there were clear differences. The majority of the *Cynorkis* spp. studied were found in soils with low NO_3_^-^N and P contents. Specifically, most *Tulasnella* OTUs, the dominant mycorrhizal lineage, were found in soil with low P content. Although this study was hindered by the small sample sizes imposed by permit restrictions, our findings have indicated avenues of future investigations. Detailed studies on other *Cynorkis* species from different regions of Madagascar with varied soil types and moisture levels will shed more light on the association between different fungal groups and members of this important genus.

The majority of *Cynorkis* species the most dominant herbaceous orchid genus in the Central Highland inselbergs of Madagascar [17]—are found in soil which is either moist or wet for at least part of the year [14]. Climate change, illegal collecting, and other anthropogenic pressures will continue to undermine orchid population resilience in the coming decades in the island nation. Detailed studies are required to understand the similarities and differences between these orchids and how they adapt to different ecological conditions. This will help develop pragmatic conservation policies underpinned by symbiotic technology and soil data for effective reintroduction/reinforcement to augment populations vulnerable to decline.

Our results advocate the need to introduce *Cynorkis* seedlings to primarily wet habitats, beginning with an explorative study to assess seedling establishment and survival. Considering that members of this genus appear to be short-lived (ephemeral) compared to other terrestrial orchids, establishing self-sustaining populations capable of anthesis and seedling recruitment from these plants is another conservation priority.

## Figures and Tables

**Table 1:** Description of collection sites detailing locality, topography, and vegetation.

Collection Site	Coordinates (Lat., Long.)	Area	Locality	Topography	Vegetation
A	S20.586765 E46.82227	Ambatofinandrahana	Magrama	Mountainous. Outcrops of marble and quartzite	Open montane grassland. Low shrubs near rock outcrops
B	S20.661717 E47.120037	Ivato	Ambodiriana	Base of wide valley. Granite slabs and large, fast-flowing river	Stands of large trees, glades, scrub, and wet grassland
C	S20.772407 E47.321264	Ambositra	Antoetra	Rolling hills with few granite outcrops	Mix of forest, grassland, farmland and lithophytic habitat
D	S20.414766 E47.166029	Ampasina	Ambohimitombo	Steep hills, no bare rock	Humid forest (degraded), grassland at forest margins
E	S20.617151 E47.434183	Amoron’i mania	Ambelatany	Steep-sided granite inselberg. Rice cultivation on lower slopes	Few small trees at lower elevation. Wet grassland above rice fields
F	S20.35.739 E46 33.863	Ambatofinandrahana	Itremo	Mountainous. Outcrops of quartzite and granite. Open grassland.	Mix of inselberg, montane grassland, and humid gallery forest

## Supplementary Materials

The following are available online at https://www.mdpi.com/article/10.3390/microorganisms9040792/s1.
