# Supplementary material for: Fungal Diversity of Selected Habitat Specific Cynorkis Species (Orchidaceae) in the Central Highlands of Madagascar
Source: Microorganisms. 2021 Apr 10;9(4):792. doi: 10.3390/microorganisms9040792 (PMC8069969; doi:10.3390/microorganisms9040792)
Supplement: Supplementary file 1 [file microorganisms-09-00792-s001.zip › Non-mycorrhiza fungi.docx]

Non-mycorrhizal fungi list

>56 cynrid AscomycotaA 1.6b(8)/2.4a(4) consensus

CCGACCTCCCAACCCTTTGTTTACCGAACCTCTGTTGCTTCGGCGGGCCCGTCTCACGACCGCCGGAGGACCGC CGACAGGTGTCCTCTGGCCCGTGTTCGCCGACAGCCAACCTCTCAAAACTCTGTATGAATCGTGTCTTTACCTC TAAGTCTATGATTTAAACAAAATCAAAAGCAAAACTTTCAACAACGGATCTCTTGGTTCTGGCATCGATGAAG AACGCAGCGAAATGCGATAAGTAATGCGAATTGCAGAATTCCAGTGAGTCATCGAATCTTTGAACGCACATTG CGCCCTTTGGTATTCCGAAGGGCATGCCTGTTCGAGCGTCATTATCACCCCTCAAGCCCCTGGCTTGGTGTTGG ACGGCTTGGTGGACTGTAAATGTCGACCCCTCCTAAAGACAATGACGGCGCCCCGCGGCACCCCCGGTACACT GAGCTTTTCGTCGAGCACGTATCGGACAAGGGTCCCCGGGACACGGTCTCTCTTTCCCACGCATAGCGGGAAA

ATTTT

>220.1.2e(21) cynspe AscomycotaA

TAGGTGAACCTGCGGAAGGATCATTAACGAGTTAGGGTCTTCTCAGGCCCGACCTCCCAACCCTTTGTTTACC GAACCTCTGTTGCTTCGGCGGGCCCGTCTCACGACCGCCGGAGGACCGCCGACAGGTGTCCTCTGGCCCGTGT CCGCCGACAGCCAACCTCTCAAAACTCTGTATGAATCGTGTCTTTACCTCTAAGTCTATGATTTAAACAAAATCA AAAGCAAAACTTTCAACAACGGATCTCTTGGTTCTGGCATCGATGAAGAACGCAGCGAAATGCGATAAGTAAT GCGAATTGCAGAATTCCAGTGAGTCATCGAATCTTTGAACGCACATTGCGCCCTTTGGTATTCCGAAGGGCAT GCCTGTTCGAGCGTCATTATCACCCCTCAAGCCCCCTGGCTTGGTGTTGGATGGCTTGGTGGACTGTAAATGTC GACCCCTCCTAAAGACAATGACGGCGCCCCGCGGCACCCCCGGTACACTGAGCTTTTCATCGAGCACGTATCG GACAAGGGTCCCCGGGACACGGTCTCTCTTTCCCACGCATAGCGGGAAAATTTTCTGAAAGGTTGA

>221.1.1b(48)purple cynuni AscomycotaB

GGGGCCCCCGGTGGACATATATAAACTCTGCTTCAATTTGTCGTCTGAGTAAATTGATTAAATCAAATCAAAAC TTTCAACAACGGATCTCTTGGTTCTGGCATCGATGAAGAACGCAGCGAAATGCGATAAGTAATGTGAATTGCA GAATTCAGTGAATCATCGAATCTTTGAACGCACATTGCGCCCTTTGGTATTCCGAAGGGCATGCCTGTTCGAGC GTCATTACACCACTCAAGCCTCGCTTGGTATTGGAGGACGCGGTTCGCCGCGCCTCTTGAATCTTTCGGCTGA GCATATCGTCTCTTAGCGTTGTGATACATATTCGCTTGTGAGGTCGGTAGTGTTTGCGCCGTTAAACCCCCAAA TTTTAAAGGTG

>222.1.3d(11)brown AscomycotaB1

CCTGCGGAGGGATCATTACTGAGTTCTGGGTCTCCAAGACCCGACCTCCAACCCTTTGTTGTCCGACTCTGTTG CCTCGGGGGTGACCCTGACCCGCTCGCGGGTTTTTGGGGCCCCCGGTGGACATATATAAAACTCTGCTTCAAT TTGTCGTCTGAGTAAATTGATTAAATCAAATCAAAACTTTCAACAACGGATCTCTTGGTTCTGGCATCGATGAA GAACGCAGCGAAATGCGATAAGTAATGTGAATTGCAGAATTCAGTGAATCATCGAATCTTTGAACGCACATTG CGCCCTTTGGTATTCCGAAGGGCATGCCTGTTCGAGCGTCATTACACCACTCAAGCCTCGCTTGGTATTGGAG GACGCGGTTCGCCGCGCCTCTTGAATCTTTCGGCTGAGCATATCGTCTCTTAGCGTTGTGATACATATTCGCTT GTGAGGTCGGTAGTGTTTGCGCCGTTAAACCCCCAAATTTTAAAGGTTGACCTCG

>222.1.3d(47) cynhyb AscomycotaC2

GGATCATTACACAATAACATATGAAGGCTGTACGCGGCTGTGCTCTCGGGCGCAGTGCTGCTGAGGCTGGAG TATTTATTTACCCTTGTCTTTTGCGCACTTGTTGTTTCCTGGGCGGGTTCGCTCGCCACCAGGACCACACTATAA ACCTTTTTTATGCAATTGTAATCAGCGTCAGTACAACAAATGTAAATCATTTACAACTTTCAACAACGGATCTCT TGGTTCTGGCATCGATGAAGAACGCAGCGAAATGCGATACGTAGTGTGAATTGCAGAATTCAGTGAATCATC GAATCTTTGAACGCACATTGCGCCCTTTGGTATTCCAAAGGGCATGCCTGTTCGAGCGTCATTTGTACCCTCAA GCTTTGCTTGGTGTTGGGCGTCTTGTCTGTTGGCTTTTTTGCCCAAAGACTCGCCTTAAAACGATTGGCAGCCG GCCTACTGGTTTCGCAGCGCAGCACATTTTTGCGCTTGCAATCAGCAAAAGAGGACGGCACTCCATCAAGACT CCTTCTCACGTT

>222.1.3d(49) cynhyb AscomycotaA3

AGGATCATTAACGAGTTAGGGTCTTCTCAGGCCCGACCTCCCAACCCTTTGTTTACCGAACCTCTGTTGCTTCG GCGGGCCCGTCTCACGACCGCCGGAGGACCGCCGACAGGTGTCCTCTGGCCCGTGTTCGCCGACAGCCAACC TCTCAAAACTCTGTATGAATCGTGTCTTTACCTCTAAGTCTATGATTTAAACAAAATCAAAAGCAAAACTTTCAA CAACGGATCTCTTGGTTCTGGCATCGATGAAGAACGCAGCGAAATGCGATAAGTAATGCGAATTGCAGAATT CCAGTGAGTCATCGAATCTTTGAACGCACATTGCGCCCTTTGGTATTCCGAAGGGCATGCCTGTTCGAGCGTC ATTATCACCCCTCAAGCCCCTGGCTTGGTGTTGGACGGCTTGGTGGACTGTAAATGTCGACCCCTCCTAAAGAC AATGACGGCGCCCCGCGGCACCCCCGGTACACTGAGCTTTTCATCGAGCACGTATCGGACAAGGGTCCCCGG GACAGCGGTCTCTCTTTCCCACGCATAGCGGGAAAATTT

>222.1.4c(8) cynhyb AscomycotaA

TGCGGAAGGATCATTAACGAGTTAGGGTCTTCTCAGGCCCGACCTCCCAACCCTTTGTTTACCGAACCTCTGTT GTTTCGGCGGGCCCGTCTCACGACCGCCGGAGGACCGCCGACAGGTGTCCTCTGGCCCGTGTTCGCCGACAG CCAACCTCTCAAAACTCTGTATGAATCGTGTCTTTACCTCTAAGTCTATGATTTAAACAAAATCAAAAGCAAAAC TTTCAACAACGGATCTCTTGGTTCTGGCATCGATGAAGAACGCAGCGAAATGCGATAAGTAATGCGAATTGCA GAATTCCAGTGAGTCATCGAATCTTTGAACGCACATTGCGCCCTTTGGTATTCCGAAGGGCATGCCTGTTCGA GCGTCATTATCACCCCTCAAGCCCCTGGCTTGGTGTTGGACGGCTTGGTGGACTGTAAATGTCGACCCCTCCTA AAGACAATGACGGCGCCCCGCGGCACCCCCGGTACACTGAGCTTTTCATCGAGCACGTATCGGAACAAGGGT CCCCCGGGA

>222.1.6d(8) cynhyb AscomycotaA (8)(9)(10) consensus

GTGAACCTGCGGAAGGATCATTAACGAGTTAGGGTCTTCTCAGGCCCGACCTCCCAACCCTTTGTTTACCGAA CCTCTGTTGCTTCGGCGGGCCCGTCTCACGACCGCCGGAGGACCGCCGACAGGTGTCCTCTGGCCCGTGTTCG CCGACAGCCAACCTCTCAAAACTCTGTATGAATCGTGTCTTTACCTCTAAGTCTATGATTTAAACAAAATCAAA AGCAAAACTTTCAACAACGGATCTCTTGGTTCTGGCATCGATGAAGAACGCAGCGAAATGCGATAAGTAATGC GAATTGCAGAATTCCAGTGAGTCATCGAATCTTTGAACGCACATTGCGCCCTTTGGTATTCCGAAGGGCATGC CTGTTCGAGCGTCATTATCACCCCTCAAGCCCCTGGCTTGGTGTTGGACGGCTTGGTGGACTGTAAATGTCGA CCCCTCCTAAAGACAATGACGGCGCCCCGCGGCACCCCCGGTACACTGAGCTTTTCATCGAGCACGTATCGGA CAAGGGTCCCCGGGACACGGTCTCTCTTTCCCACGCATAGCGGGAAAATTTTCTGAAAGGTTGACCTCGGATCAG

G

>217.1 cynfle basidiomycete consensus 2b(10)/4c(10)

TCTTTCGAGACATGTGCACGTTTGCCACCAATTTTAACCACCTGTGCACCTTTTGTAGACTGGAAACGTTTCTCG AGGCAACTCGGATTGAGGACTGCTGTGCGCAAGCCGGCTGTTCTTGTGTTTCTCAGTCTATGTTTTTATATACT CCTAATGAATGTATCAGAATGTATTGCTGGGCCTTAGTGCCTTTAAATCAAATACAACTTTCAACAACGGATCT CTTGGCTCTCGCATCGATGAAGAACGCAGCGAAATGCGATAAGTAATGTGAATTGCAGAATTCAGTGAATCAT CGAATCTTTGAACGCACCTTGCGCTCCTTGGTATTCCGAGGAGCATGCCTGTTTGAGTGTCATTAAATTCTCAA CTATACAAGTTTTTTAAACATGTATAGCTTGGATCTTGGGATTTGCGGGCTTTCACGAGTCGGCTATCCTTAAA TGCATTAGCAGAGCTTTTGCCGCTAACCACTGGTGTGATAATTATCTACGCCTTTGAGTTGCGACATATTGAGG CTTAGCTTCTAACCGTCCGCAAGGACAATATTTGACAATCTGACCTCAAATCAGGT

>201.5.wp(26) cynfas *Dioszegia*

TCCGTAGGTGAACCTGCGGAAGGATCATTAATAAATGGCGAAGCTTTCGAGCCTAGCTTTTATCATCCTATACA CCTGTGAACTATTTGCCTTCGGGCACCAAACAAACATAATGTAATGAATGTAATCTACTATAATAAATACAACT TTCAACAACGGATCTCTTGGCTCTCACATCGATGAAGAACGCAGCGAAATGCGATAAGTAATGTGAATTGCAG AATTCAGTGAATCATCGAATCTTTGAACGCAAATTGCGCCCTTTGGTATTCCGAAGGGCATGCCTGTTTGAGTG TCTTATAAACCTCACCCCTAATGTTTTATTACAATAGGTTGGTGCTTTGGGTGTTGCCATTTACCTGGCTCGCCT

TAAATGACTAAGTGGAATGCGTAATAAGTTCTCGCAACCCTCCACTCTAATACAATTTATATTCTGACCTCAAAT C

>221.1.4b(22) cynuni *Coprinopsis* 1a

ACTGCGGAAGGATCATTATTGAATAAATCTGACATGGTTGTAGCTGGCTCTTCCGGGCATGTGCTCGCCTGTC ACCTTTATCTTTCCACCTGTGCACACACTGGTAGTCCTGGATACCTCTCGCCGCAAGGCGGATACAGAGACTGC TGTTCAGCCTCCGAAAGGGGGAGGTCGGCTATCTCTGAATTTCCAGGTCTATGATTATCACACACCCCAATTGA ATGTTACAGAATGTTATCAAAGGGCCTTGTGCCTATAAATCTATACAACTTTCAGCAACGGATCTCTTGGCTCT CGCATCGATGAAGAACGCAGCGAAATGCGATAAGTAATGTGAATTGCAGAATTCAGTGAATCATCGAATCTTT GAACGCACCTTGCGCTCCTTGGTATTCCGAGGAGCATGCCTGTTTGAGTGTCATTAAATTCTCAACCTCACCAA CTTTGTTGTGTGAAGGCTTGGATGTGGGGGTTTGCAGGTCCACACTTGTGTGGTCTGCTCCTCTGAAACATATT AGTGGGTTAGCCCCCTAATCTATTGGTG

>222.7.wp(6) cynhyb *Coprinus* 1b

GTGAACCTGCGGAAGGATCATTATTGAATAAATCTGACATGGTTGTAGCTGGCTCTTCGGGGCATGTGCTCGC CTGTCACCTTTATCTTTCCACCTGTGCACACACTGTAGTCCTGGATACCTCTCGCCGCAAGGCGGATACAGAGA CTGCTGTTCAGCCTCCGAAAGGGGGAGGTCGGCTATCTCTGAATTTCCAGGTCTACGATTATCACACACCCCA ATTGAATGTTACAGAATGTTATCAAAGGGCCTTGTGCCTATAAATCTATACAACTTTCAGCAACGGATCTCTTG GCTCTCGCATCGATGAAGAACGCAGCGAAATGCGATAAGTAATGTGAATTGCAGAATTCAGTGAATCATCGA ATCTTTGAACGCACCTTGCGCTCCTTGGTATTCCGAGGAGCATGCCTGTTTGAGTGTCATTAAATTCTCAACCTC ACCAACTTTGTTGTGTGAAGGCTTGGATGTGGGGGTTTGCAGGTCCACACTTGTGTGGTCTGCTCCTCTGAAA CATATTAGTGGGTTAGCCCCCTAATCTATTGGTGTGATAATTATCTACGCCGTGGATTTGGAAGGCTATTATGG ACCTGCTTCTAACCGTCCTCTGGACAACTTTGACAATTTGGCCTCAAATCAGG

>222.1.3d(41) C. x ranaivosonii *Deconica* 1a

CCTGCGGAAGGATCATTATTGAATAAACCTGATGTAGTTGTAGCTGGCTCTCTCGGGAGCATGTGCTCGCTCA TCATCTTTATATTTCCACCTGTGCACCTTTTGTAGACCCAGGCGACTTGAGCAATCAAGTCATTTGGGCCTATGT TTTATCATATACCCCATAGTATGTATCAGAATGTATCAATGGGCTTCGTGCCTATAAACACTATACAACTTTCAG CAACGGATCTCTTGGCTCTCGCATCGATGAAGAACGCAGCGAAATGCGATAAGTAATGTGAATTGCAGAATTC AGTGAATCATCGAATCTTTGAACGCACCTTGCGCTCCTTGGTATTCCGAGGAGCATGCCTGTTTGAGTGTCATT AAATTCTCAACCTCTCTAGTTTTTTTGACTAGTAGATGGCTTGGATGTGGGGGTTTTTTGCAGGCTTTCACAAG TCAGCTCCCCTTAAATATATTAGCCGGTGCCCTCTAACCGTCTATTGGTGTGATAATTATCTACGCCGTGGACT ATTAGACTTGGTTGTACTGCTTA

>222.1.1k(7) C. x ranaivosonii *Deconica* 1b

ATCATTATTGAAATAAACCTGATGTAGTTGTAGCTGGCTCTCTCGGGAGCATGTGCTCGCTCATCATCTTTATA TTTCCACCTGTGCACCTTTTGTAGACCCAGGCGACTTGAGCAATCAAGTCATTTGGGCCTATGTTTTATCATATA CCCCATAGTATGTATCAGAATGTATCAATGGGCTTCGTGCCTATAAACACTATACAACTTTCAGCAACGGATCT CTTGGCTCTCGCATCGATGAAGAACGCAGCGAAATGCGATAAGTAATGTGAATTGCAGAATTCAGTGAATCAT CGAATCTTTGAACGCACCTTGCGCTCCTTGGTATTCCGAGGAGCATGCCTGTTTGAGTGTCATTAAATTCTCAA CCTCTCTAGTTTTTTGACTAGTAGATGGCTTGGATGTGGGGGTTATTTGCAGGCTTTCACAAGTCAGCTCCCCT TAAATGTATTAGCCGGTGCCCTCTAACCGTCTATTGGTGTGATAATTATCTACGCCGTGGACTATTAGACTTGG TTGTACTGCTTATAACCGTCCTGTACTGGACAATCTATGACATTTTGACCTCAAAT

>201.2.wp(3) cynfas *Aplosporella*

GAACCTGCGGAAGGATCATTACCGAGTTCTGGGTTCTCTCATCGAGGCCCGCCTCTTCCCACCCTTTGTGAACT TGACCTCTGTTGCTTTGGCGTGGCCAGCGCTGTAAAAAGCGCGGCCGGCGGGGCCTGGCCCCCGCTGGCAAG CTTGCCCGCCAGAGGACTATCAAACTCTTGTCAGTAAACGATGCAGTCTGATCAAACATTTAATTGTTTAAAAC TTTCAACAACGGATCTCTTGGTTCTGGCATCGATGAAGAACGCAGCGAAATGCGATAAGTAATGTGAATTGCA GAATTCAGTGAATCATCGAATCTTTGAACGCACATTGCGCCCTATGGTATTCCGTAGGGCATGCCTGTTCGAGC GTCATTACACACCTCAAGCTCTGCTTGGTATTGGGCGTCGTCCCCTAAACGGACGTGCCTCAAAGACCTCGGC GGTGGCGTCTTTGCCTCAAGCGTAGTAATACTTTTATCTCGCTTTGGAGTCGAAGGGCGTCGCCCGCCGGACG AAACCTTTATTATTTCTATCAAGGTTGACCTCGGAT

>222.7.wp(4) C. x ranaivosonii *Cladosporium*

GTGAACCTGCGGAGGGATCATTACAAGTGACCCCGGTCTAACCACCGGGATGTTCATAACCCTTTGTTGTCCG ACTCTGTTGCCTCCGGGGCGACCCTGCCTTCGGGCGGGGGCTCCGGGTGGACACTTCAAACTCTTGCGTAACT TTGCAGTCTGAGTAAACTTAATTAATAAATTAAAACTTTTAACAACGGATCTCTTGGTTCTGGCATCGATGAAG AACGCAGCGAAATGCGATAAGTAATGTGAATTGCAGAATTCAGTGAATCATCGAATCTTTGAACGCACATTGC GCCCCCTGGTATTCCGGGGGGCATGCCTGTTCGAGCGTCATTTCACCACTCAAGCCTCGCTTGGTATTGGGCA ACGCGGTCCGCCGCGTGCCTCAAATCGACCGGCTGGGTCTTCTGTCCCCTAAGCGTTGTGGAAACTATTCGCT AAAGGGTGTTCGGGAGGCTACGCCGTAAAACAACCCCATTTCTAAGGTTGACCTCGGATCAGTAG

>57.2.4b cyngra *Muyocopron* (8)(14) consensus

GGTTCTTCAAACCTCTGCGTACCAAACCTTTCAGTTGCCTCCGGCGGCCCTGGGCCGACGCGGCGCGCGACCC TCCCTCGCGGGGGGGCCGCTCCCGCGGCGGACCACCCGCCGGGCGGTCGTAAACCAAACCTTTTGTCGAGAT GGCATCGTCTAATTTCTTCATAACAAAATATGAAATACAACTTTCAACAATGGATCTCTTGGCTCCGGCATCGA TGAAGAACGCAGCGAAATGCGATAACTAGTGTGAATTGCAGATTTCAGTGAATCATCGAGTCTTTGAACGCAC ATTGCGCCTCTTGGTATTCCTCGAGGCATGCCTGTTCGAGCGTCGTTACGCCCCTCAAGCGCAAGCTTGGTGTT GGGGATCGCCCCTGAGATACGGCGGCGGCCCTTAAATGCATCGGCGGTGCTGGTGTCAGCCCGGAGCGCAG CAGACATGCGGCTTCCAGGCGACCACGCGCCCGCCGGA

>222.1.1k(16) cynhyb PleosporalesA1

CCGTAGGTGAACCTGCGGAAGGATCATTAATTACGAAAGCTATAGCTCCGAAAGGAGTTATTGCATCCACCCT TTGTCTACTTGTACCTCTTGTTGTTTCCTCGGCAGGCTTGCCTGCCGCTAGGAACCCCATAAACCCTTGTATTCA TAGTATTAAAATCTCTGATAACTATTTAAATTATTACAACTTTCAACAATGGATCTCTTGGTTCTGGCATCGATG AAGAACGCAGCGAAATGCGAAAAGTAGTGTGAATTGCAGAATTCCGTGAATCATCGAATCTTTGAACGCACA TTGCGCCCCTCGGTATTCCGTGGGGCATGCCTGTTCGAGCGTCATTTACACCCTCAAGCTCTGCTTGGTGTTGG GCGTCTGTCCCGCTTCGTGCGTGGACTCGCCCCAAAGTCATTGGCAGCGGTCGTGCCAGCTTCTCGCGCAGCA CATTAGCGTTTCTTGAAGTTTGGTGGATCAGCATCCAGTAAGCTCTTTTTATGACTTGACCTCGGATCA

>201 cynfas PleosporalesA2 2.wp(4)(8)/5.wp(34) consensus

TCCGTAGGTGAACCTGCGGAAGGATCATTAATTACGAAAGCTATAGCTCCGAAAGGAGTTATTGCATCCACCC TTTGTCTACTTGTACCTCTTGTTGTTTCCTCGGCAGGCTTGCCTGCCGCTAGGAACCCTCATAAACCCTTGTATT AATAGTATTAAAATCTCTGATAACTATTTAAATTATTACAACTTTCAACAATGGATCTCTTGGTTCTGGCATCGA TGAAGAACGCAGCGAAATGCGAAAAGTAGTGTGAATTGCAGAATTCCGTGAATCATCGAATCTTTGAACGCA CATTGCGCCCCTCGGTATTCCGTGGGGCATGCCTGTTCGAGCGTCATTTACACCCTCAAGCTCTGCTTGGTGTT GGGCGTCTGTCCCGCTTCGTGCGTGGACTCGCCCCAAAGTCATTGGCAGCGGTCGTGCCAGCTTCTCGCGCAG CACATTTGCGTTTCTTGAAGCTTGGTGGATCAGCATCCAGTAAGCTCTTTTATGACTTGACCTCGGATCAGGTA

>222.1.1k(8) cynhyb *Curvularia* A

AGGGATCATTACACAATAACATATGAAGGCTGTACGCGGCTGGGCTCTCGGGCGCAGTGCTGCTGAGGCTGG ATTATTTATTCACCCGTTGTCTTTTGCGCACTTGTTGTTTCCTGGGCGGGTTCGCCCGCCGCCAGGACCACACCA TAAACCTTTTTTTATGCAGTTGCAATCAGCGTCAGTACAACAAATGTAAATCATTTACAACTTTCAACAACGGAT CTCTTGGTTCTGGCATCGATGAAGAACGCAGCGAAATGCGATACGTAGTGTGAATTGCAGAATTCAGTGAATC ATCGAATCTTTGAACGCACATTGCGCCCTTTGGTATTCCAAAGGGCATGCCTGTTCGAGCGTCATTTGTACCCT CAAGCTTTGCTTGGTGTTGGGCGTCTTGTCTTTTGGGCTCTTTGCCCAAAGACTCGCCTCAAAACGATTGGCAG CCGGCCTACTGGTTTCGCAGCGCAGCACATTT

>58.1.1d(2) cynrid *Curvularia* B

GTGAACCTGCGGAGGGATCATTACACAAAAAATATGAAGGCTGCAACCGCCAGTTTTGGCGGGGAAGCTGAA TTATTTTTCACCCATGGTCTTTTGCGCACTTGTTGTTTCCTGGGCGGGTTCGCCCGCCACCAGGACCACACCATA

AACCTTTTTTATGCAGTTGCAATCAGCGTCAGTATAACAAATGTAAATCATTTACAACTTTCAACAACGGATCTC TTGGTTCTGGCATCGATGAAGAACGCAGCGAAATGCGATACGTAGTGTGAATTGCAGAATTCAGTGAATCATC GAATCTTTGAACGCACATTGCGCCCTTTGGTATTCCAAAGGGCATGCCTGTTCGAGCGTCATTTGTACCCTCAA GCTTTGCTTGGTGTTGGGCGTTTTGTCTTTGGCTTTTGCCCAAAGACTCGCCTTAAAACGATTGGCAGCCGGCC TACTGGTTTCGGAGCGCAGCACATTTTTGCGCTTGCAACTAGCTAAAGAGGCCAGCAATCCATCAAGACCTTCT TCTCACTTTTGACCTCGGATCA

>71.1.1b(3) cyngib *Alternaria*

AGGTGAACCTGCGGAGGGATCATTACACAATATGAAAGCGGGCTGGAATCCTTTGGGGTTACAGCCTTGCTG AATTATTCACCCGTGTCTTTTGCGTACTTCTTGTTTCCTTGGTGGGTTCGCCCACCATAGGACAAACCATTAAAC CTTTTGTAATTGCAATCAGCGTCAGTAAAAAAATTAATAATTACAACTTTTAACAACGGATCTCTTGGTTCTGGC ATCGATGAAGAACGCAGCGAAATGCGATAAGTAGTGTGAATTGCAGAATTCAGTGAATCATCGAATCTTTGA ACGCACATTGCGCCCTTTGGTATTCCAAAGGGCATGCCTGTTCGAGCGTCATTTGTACCCTCAAGCTTTGCTTG GTGTTGGGCGTCTTGTCTCCAGTTCGCTGGAGACTCGCCTTAAAGTAATTGGCAGCCGGCCTACTGGTTTCGG AGCGCAGCACAAGTCGCGCTCTCTTCCAGCGAAGGTCAGCATCCACAAAGCCTTTTTTCAAC

>201.2.wp cynfas Pleosporales *Stagonospora* (6)(7) consensus

ACCTGCGGAAGGATCATTAATTACGAAAGCTATAGCTCCGAAAGGAGTTATTGCATCCACCCTTTGTCTACTTG TACCTCTTGTTGTTTCCTCGGCAGGCTTGCCTGCCGCTAGGAACCCTCATAAACCCTTGTATTAATAGTATTAAA ATCTCTGATAACTATTTAAATTATTACAACTTTCAACAATGGATCTCTTGGTTCTGGCATCGATGAAGAACGCA GCGAAATGCGAAAAGTAGTGTGAATTGCAGAATTCCGTGAATCATCGAATCTTTGAACGCACATTGCGCCCCT CGGTATTCCGTGGGGCATGCCTGTTCGAGCGTCATTTACACCCTCAAGCTCTGCTTGGTGTTGGGCGTCTGTCC CGCTTCGTGCGTGGACTCGCCCCAAAGTCATTGGCAGCGGTCGTGCCAGCTTCTCGCGCAGCACATTTGCGTT TCTtGAAGCTTGGTGGATCAGCATCCAGTAAGCTCTTTTATGACTTGACCTCGATCAGG

>221.1 cynuni Toxicocladosporium A 1b(30)(32)(34)(35)(37)(39)(46)(47)(49)(50)/ 4b(19)(24)(37)(39)

consensus

GTCTCCGTAGGTGAACCTGCGGAGGGATCATTACAAAAGTAAACCCGCCTCCCGGTCTCGGCCGTCTGGCGG GCTACTACCAACCCTTTGTTGTCCGACCGCGTTGCCTCCGGGGCGACCCTGACCTCCGGGTCATGGGGGACCC CGGGTGGACACCCAAACTCTGCGTAACTTTGTAGTCTGAGTAAACAAGTTAATAAACTAAAACTTTCAACAAC GGATCTCTTGGTTCTGGCATCGATGAAGAACGCAGCGAAATGCGATAAGTAATGTGAATTGCAGAATTCAGT GAATCATCGAATCTTTGAACGCACATTGCGCCCCGTGGTATTCCGCGGGGCATGCCTGTTCGAGCGTCATTTC ACCACTCAAGCCTCGCTTGGTATTGGGCGACGCGGTCCGCCGCGCGCCTCAAATCGACCGGCTGGGTCGATC GCACCCTCAGCGTTGTGGAAACTATTCGCAAAAGGAGTTGTGGTCGGTCACGCCGTGAAACAAACCAATTTTT CAATGGTTGACCTCGGATCAGGTAG

>222.1 cynhyb *Toxicocladosporium* A 1b(11)/3d(18)(32) consensus

CCGTAGGTGAACCTGCGGAGGGATCATTACAAAAGTAAACCCGCCTCTCGGTCTCGGCCGTCTGGTTGGGCTA CTACCAACCCTTTGTTGTCCGACCGCGTTGCCTCCGGGGCGACCCTGACCTCCGGGTCATGGGGGACCCCGGG TGGACACCCAAACTCTGCGTAACTTTGTAGTCTGAGTAAACAAGTTAATAAACTAAAACTTTCAACAACGGATC TCTTGGTTCTGGCATCGATGAAGAACGCAGCGAAATGCGATAAGTAATGTGAATTGCAGAATTCAGTGAATCA TCGAATCTTTGAACGCACATTGCGCCCCGTGGTATTCCGCGGGGCATGCCTGTTCGAGCGTCATTTCACCACTC AAGCCTCGCTTGGTATTGGGCGACGCGGTCCGCCGCGCGCCTCAAATCGACCGGCTGGGTCGATCGCACCCT CAGCGTTGTGGAAACTATTCGCAAaAGGAGTTGTGgTCGGTCACGCCGTGAAACAAACCAATTTTCAATGGTT GACCTCGGATCAGGT

>222.1.1k(6) cynhyb Dothideomycetes

TGCGGAAGGATCATTACCTTTGGCTGCCGTGACCGCTTCGGCGTCGCGACAGTCATTATATAGTGACGCCTCC GTGTCCGGGAAACCGGCGCGGGGCTGACCTAACCCTTCTCTACGAGTACCTAACATTCTCCTTCGGCGGGGCA ACCCGCCGCTGGAATTGAAGAACCAACTTGCATTTAGCATTACCTGTTCTGATAACAATTAATTATTACAACTTT CAACAATGGATCTCTTGGCTCTGGCATCGATGAAGAACGCAGCGAAATGCGATAAGTAGTGTGAATTGCAGA ATTCAGTGAATCATCGAATCTTTGAACGCACATTGCGCCCCTTGGTATTCCATGGGGCATGCCTGTTCGAGCGT CATTTACACCCTCAAGCACTGCTTGGTGTTGGGCGTCTGTCCCGCCTTTGCGCGTGGACTCGCCCCAAAGTCAT TGGCAGCGGTCTCTGGCACCTCAACGCGTAGTACAATGCGTTTCATTGGGGGTGCCGTG

>221.1.3b(1) cynuni *Eutiarosporella*

CGGAAGGATCATTACCGAGTTCTCGGGCTCCGGCCCGAAATCTCCAACCCTTTGCCTACCTCACCTCTGTTGCT TCGGCGCCGCGGCGGCCTCGCGCCGCCGCCCGCCGGAGGACCGTCAAACCCCAGTCAGTGAACGTCGCAGTC TGAGAACAAGTTTAAACAAACTAAAACTTTCAACAACGGATCTCTTGGTTCTGGCATCGATGAAGAACGCAGC GAAATGCGATAAGTAATGTGAATTGCAGAATTCAGTGAATCATCGAATCTTTGAACGCACATTGCGCCCTTTG GTATTCCGAAGGGCATGCCTGTTCGAGCGTCATTACAACCCTCAAGCTCTGCTTGGTATTGGGCACCGTCCGC CTCCCCGCGGACGCGCCTCGAAGACCTCGGCGGCGGCGTCTGGCCCTCAAGCGTAGTAGTGTCTATCTCGCTT TGGAGCGCCTGGCGTCGCCCGCCGGACGAACCTTCTGAACCACTTCTCAA

>222.1.1b(6) cynhyb *Aspergillus* (6)(7) consensus

AGGTGAACCTGCGGGAAGGATCATTACCGAGTGAGGGCCCTCTGGGTCCAACCTCCCACCCGTGTCTATTGTA CCATTGTTGCTTCGGCGGGCCCGCCGTTTCGACGGCCGCCGGGGAGGCCTCGCGCCCCCGGGCCCGCGCCCG CCGAAGACCCCAACATGAACGCTGTTCTGAAAGTATGCAGTCTGAGTTGATTATCATAATCAGTTAAAACTTTC AACAACGGATCTCTTGGTTCCGGCATCGATGAAGAACGCAGCGAAATGCGATAAATAATGTGAATTGCAGAA TTCAGTGAATCATCGAGTCTTTGAACGCACATTGCGCCCCCTGGTATTCCGGGGGGCATGCCTGTCCGAGCGT CATTGCTGCCCTCAAGCACGGCTTGTGTGTTGGGCCGCCGTCCCCGGTTTCCCCCGGGGACGGGCCCGAAAG GCAGCGGCGGCACCGCGTCCGGTCCTCGAGCGTATGGGGCTTTGTCACCCGCTCTGTAGGCCCGGCCGGCGC CAGCCGACACCCAACTTTATTTCTAAGGt

>73.1.5b(12) cynpap Eurotiomycetes

GAGTTAGGGTAGTTATTCACTGCCCGACCTCCCAACCCTGTGTTTACCACACTTTGTCGTTGCTTCGGCGGACC GGTCGACCAACTGGTCGTGACCGCCGGGGGCTGGCTCCGTCCCCCCTGGAGCGCGTCCGCCGATGGCCCAAC CACAAACTCTTGTACCGAAACGTGTCGTCTGAATAATTGAGAAATCAAAAATCAAAACTTTCAACAACGGATCT CTTGGTTCTGGCATCGATGAAGAACGCAGCGAAATGCGATAAGTAATGCGAATTGCAGAATTTCCGTGAGTC ATCGAATCTTTGAACGCACATTGCGCCCATTGGTATTCCGATGGGCATGCCTGTTCGAGCGTCATTATCCTCCC TCAAACTTCGTGTTTGGTGTTGGACCGCGTTGGTCGAGCGACCAACTGGTCTCAAAGATAATGACGGCGTCCG TGGGACCCTCGGTGCAACGAGCTTCTAGGAGCACGCATCGAGTTTCAAGG

>221.1.1b(45) cynuni Talaromyces 1b(45)/4b(9) consensus

AACCTGCGGAAGGATCATTACCGAGTGCGGGCCCCTCGTGGCCCAACCTCCCACCCTTGTCTCTATACACCTGT TGCTTTGGCGGGCCCACCGGGGCCACCTGGTCGCCGGGGGACGTTCGTCCCCGGGCCCGCGCCCGCCGAAGC GCTCTGTGAACCCTGATGAAGATGGGCTGTCTGAGTACTATGAAAATTGTCAAAACTTTCAACAATGGATCTCT TGGTTCCGGCATCGATGAAGAACGCAGCGAAATGCGATAAGTAATGTGAATTGCAGAATTCCGTGAATCATC GAATCTTTGAACGCACATTGCGCCCCCTGGCATTCCGGGGGGCATGCCTGTCCGAGCGTCATTTCTGCCCTCA AGCACGGCTTGTGTGTTGGGTGCGGTCCCCCCGGGGGCCTGCCCGAAAGGCAGCGGCGACGTCCGTCTGGTC CTCGAGCGTATGGGGCTTTGTCACTCGCTCGGGAAGGACTGGCGGGGGTTGGTCACCACCACAAAATTTACC ACGGTTGACCTCGGATCAGGTAGGAGT

>57.2.2e(11) cyngra Penicillium C1

TTCGGCGGGCCCGCCTCACGGCCGCCGGGGGGCTTCTGCCCTCTGGCCCGCGCCCGCCGAAGACACCATTGA ACGCTGTCTGAAGATTGCAGTCTGAGCAATTAGCTAAATAAGTTAAAACTTTCAACAACGGATCTCTTGGTTCC GGCATCGATGAAGAACGCAGCGAAATGCGATACGTAATGTGAATTGCAGAATTCAGTGAATCATCGAGTCTT TGAACGCACATTGCGCCCCCTGGTATTCCGGGGGGCATGCCTGTCCGAGCGTCATTGCTGCCCTCAAGCACGG CTTGTGTGTTGGGCCTCCGTCCTCCTCCCGGGGGACGGGCCCGAAAGGCAGCGGCGGCACCGCGTCCGGTCC TCGAGCGTATGGGGCTTCGTCACCCGCTCTTGTAGGCCCGGCCGGCGCTTGCCGACACATCAATCTTTTTTCCA GGTTGACCTCGGATC

>222.1 cynhyb *Penicillium* C2 1b(1)(19)/1k(4) consensus

AACCTGCGGAAGGATCATTACCGAGTGAGGGCCCTCTGGGTCCAACCTCCCACCCGTGTTTATCGTACCTTGTT GCTTCGGCGGGCCCGCCGTTCCGGCCGCCGGGGGGCATCCGCCCCCGGGCCCGCGCCCGCCGAAGACACCAT TGAACGCTGTCTGAAGATTGCAGTCTGAGCGATTAGCTAAATCAGTTAAAACTTTCAACAACGGATCTCTTGGT TCCGGCATCGATGAAGAACGCAGCGAAATGCGATAAGTAATGTGAATTGCAGAATTCAGTGAATCATCGAGT CTTTGAACGCACATTGCGCCCCCTGGTATTCCGGGGGGCATGCCTGTCCGAGCGTCATTGCTGCCCTCAAGCA CGGCTTGTGTGTTGGGCCCCGCCCCCCGGCTACCGGGGGGCGGGCCCGAAAGGCAGCGGCGGCACCGCGTC CGGTCCTCGAGCGTATGGGGCTTCGTCACCCGCTCTGTAGGCCCGGCCGGCGCCCGCCGGCGACCCCCCTCAA

TCTTTCTCAGGTTGACCTCGGATC

>217.1.4c(5) cynfle *Penicillium* C3

TTCCGTAGGTGAACCTGCGGAAGGATCATTACCGAGTGAGGGCCCTCTGGGTCCAACCTCCCACCCGTGTTTA TCGTACCTTGTTGCTTCGGCGGGCCCGCCTCACGGCCGCCGGGGGGCATCCGCCCCCGGGCCCGCGCCCGCC GAAGACACCATTGAACGCTGTCTGAAGATTGCAGTCTGAGCATCTTAGCTAAATCAGTTAAAACTTTCAACAA CGGATCTCTTGGTTCCGGCATCGATGAAGAACGCAGCGAAATGCGATACGTAATGTGAATTGCAGAATTCAGT GAATCATCGAGTCTTTGAACGCACATTGCGCCCCCTGGTATTCCGGGGGGCATGCCTGTCCGAGCGTCATTGC TGCCCTCAAGCACGGCTTGTGTGTTGGGCCTCCGCCCCCCGGCTCCCGGGGGGCGGGCCCGAAAGGCAGCGG CGGCACCGCGTCCGGTCCTCGAGCGTATGGGGCTTCGTCACCCGCTCCGTAGGCCCGGCCGGCGCCCGCCGG CGACCCCCCTCAATCTTTCCAGGTTGACCTCGGATCAGT

>221.1 cynuni *Penicillium* B 1b(44)/3b(4)

GAACcTGCGGAAGGATCATTACCGAGTGAGGGCCCTCTGGGTCCAACCTCCCACCCATGTTTATTGTACCTTGT TGCTTCGGCGGGCCCGCCTTTGTGGCCGCCGGGGGGCTCTGCCCCCGGGCCCGCGCCCGCCGAAGACACCTA GAACTCTGTCTGAAGATTGCAGTCTGAGTGAAAATATAAATTATTTAAAACTTTCAACAACGGATCTCTTGGTT CCGGCATCGATGAAGAACGCAGCGAAATGCGATACGTAATGTGAATTGCAGAATTCAGTGAATCATCGAGTC TTTGAACGCACATTGCGCCCTCTGGTATTCCGGAGGGCATGCCTGTCCGAGCGTCATTGCTGCCCTCAAGCAC GGCTTGTGTGTTGGGCCCCGTCCTCCGCTCCCGGGGGACGGGCCCGAAAGGCAGCGGCGGCACCGCGTCCG GTCCTCGAGCGTATGGGGCTTTGTCACCCGCTCTGTAGGCCCGGCCGGCGCTTGCCGATCAACCAAACTTTTA TCCAGGTT

>217.1 cynfle *Penicillium* B 2b(11)(13)(19)(21)/4c(11) consensus

taGGTGAACCTGCGGAAGGATCATTACCGAGTGAGGGCCCTCTGGGTCCAACCTCCCACCCATGTTTATTGTAC CTTGTTGCTTCGGCGGGCCCGCCTTTGTGGCCGCCGGGGGGCTCTGCCCCCGGGCCCGCGCCCGCCGAAGAC ACCTAGAACTCTGTCTGAAGATTGCAGTCTGAGTGAAAATATAAATTATTTAAAACTTTCAACAACGGATCTCT TGGTTCCGGCATCGATGAAGAACGCAGCGAAATGCGATACGTAATGTGAATTGCAGAATTCAGTGAATCATC GAGTCTTTGAACGCACATTGCGCCCTCTGGTATTCCGGAGGGCATGCCTGTCCGAGCGTCATTGCTGCCCTCA AGCACGGCTTGTGTGTTGGGCCCCGTCCTCCGCTCCCGGGGGACGGGCCCGAAAGGCAGCGGCGGCACCGC

GTCCGGTCCTCGAGCGTATGGGGCTTTGTCACCCGCTCTGTAGGCCCGGCCGGCGCTTGCCGATCAACCAAAC TTTTATCCAGGTTGACCTCGGATCA

>222.1.6d(12) cynhyb *Penicillium* A

AGGTGAACCTGCGGAAGGATCATTACCGAGTGCGGGCCCTCGCGGCCCAACCTCCCACCCTTGTCTCTATACA CCTGTTGCTTCGGCGGGCCCACCGGGGCCACCTGGTCGCCGGGGGACGCACGTCCCCGGGCCCGCGCCCGCC GAAGCGCTGTGAACCCTGATGAAGATGGACTGTCTGAGTACTATGAAAATTGTCAAAACTTTCAACAATGGAT CTCTTGGTTCCGGCATCGATGAAGAACGCAGCGAAATGCGATAAGTAATGTGAATTGCAGAATTCCGTGAATC ATCGAATCTTTGAACGCACATTGCGCCCCCTGGCATTCCGGGGGGCATGCCTGTCCGAGCGTCATTTCTGCCCT CAAGCACGGCTTGTGTGTTGGGTGTGGTCCCCCCCGGGACCTGCCCGAAAGGCAGCGGCGACGTCCGTCTGG TCCTCGAGCGTATGGGGCTCTGTCACTCGCTCGGGAAGGACCTGCGGGGGTTGGTCACCACCATATTTTACCA CGGTT

>220.1.3d(1) cynspe *Penicillium* A

GCGGAAGGATCATTACCGAGTGCGGGCCCTCGCGGCCCAACCTCCCACCCTTGTCTCTATACACCTGTTGCTTC GGCGGGCCCACCGGGGCCACCTGGTCGCCGGGGGACGCACGTCCCCGGGCCCGCGCCCGCCGAAGCGCTGT GAACCCTGATGAAGATGGACTGTCTGAGTACTATGAAAATTGTCAAAACTTTCAACAATGGATCTCTTGGTTCC GGCATCGATGAAGAACGCAGCGAAATGCGATAAGTAATGTGAATTGCAGAATTCCGTGAATCATCGAATCTTT GAACGCACATTGCGCCCCCTGGCATTCCGGGGGGCATGCCTGTCCGAGCGTCATTTCTGCCCTCAAGCACGGC TTGTGTGTTGGGTGTGGTCCCCCCCGGGACCTGCCCGAAAGGCAGCGGCGACGTCCGTCTGGTCCTCGAGCG TATGGGGCTCTGTCACTCGCTCGGGAAGGACCTGCGGGGGTTGGTCACCACCATATTTTACCACGGTTGACCT

>221.1.1b(29) cynuni *Penicillium* A

AGGTGAAcCTGCGGAAGGATCATTACCGAGTGCGGGCCCTCGCGGCCCAACCTCCCACCCTTGTCTCTATACA CCTGTTGCTTCGGCGGGCCCACCGGGGCCACCTGGTCGCCGGGGGACGCACGTCCCCGGGCCCGCGCCCGCC GAAGCGCTGTGAACCCTGATGAAGATGGACTGTCTGAGTACTATGAAAATTGTCAAAACTTTCAACAATGGAT CTCTTGGTTCCGGCATCGATGAAGAACGCAGCGAAATGCGATAAGTAATGTGAATTGCAGAATTCCGTGAATC ATCGAATCTTTGAACGCACATTGCGCCCCCTGGCATTCCGGGGGGCATGCCTGTCCGAGCGTCATTTCTGCCCT CAAGCACGGCTTGTGTGTTGGGTGTGGTCCCCCCCGGGACCTGCCCGAAAGGCAGCGGCGACGTCCGTCTGG TCCTCGAGCGTATGGGGCTCTGTCACTCGCTCGGGAAGGACCTGCGGGGGTTGGTCACCACCATATTTTACCA CGGTTGACCTCGATCAGGTAGG

>57.3.1c cyngra *Exophiala* (1)(2) consensus

GGGGGGTCTTCGTACCCCTTGGCCCGTGCTCGTCGATAGCCCCCCCAAATTTAAAAAATCTTAACTAAACGTGT CTTGAATCTAAGTATTATTGTTAAATAAAAAACAAAACTTTCAACAACGGATCTCTTGGTTCTGGCATCGATGA AGAACGCAGCGAAATGCGATAAGTAATGCGAATTGCAGAATTCCAGTGAGTCATCGAATCTTTGAACGCACA TTGCGCCCTTTGGTATTCCGAAGGGCATGCCTGTTCGAGCGTCATTATCACCCCTCAAGCCCTCGGCTTGGTGT TGGACGGTTTGGTGGAGACCCCCTTTGCGGGCTTCTACCCCTCCCAAAGACAATGACGGCGGCCTCGTTGGAC CCCCGGTACACTGAGTTCTTCACGGGACACGTATCGGACTACATGGGTTTACGGGACACGGTCTGCCTCCCCT CAGGGA

>217.1.5b(3) cynfle *Cladophialophora*

GTTTCCGTAGGTGAACCTGCGGAAGGATCATTAACGAGTTAGGGTCTTCTCAGGCCCGACCTCCCAACCCTTT GTTTACTGAACCTCTGTTGCTTCGGCGGGCCCGTCTCACGACCGCCGGAGGACCGCCGACAGGTGTCCTCTGG CCCGTGTCCGCCGACAGCCAACCTCTCAAAATTCTGTATGAATCGTGTCCTTTATCTCTAAGTCTATGATTTAAA CAAATCAAAAGCAAAACTTTCAACAACGGATCTCTTGGTTCTGGCATCGATGAAGAACGCAGCGAAATGCGAT AAGTAATGCGAATTGCAGAATTCCAGTGAGTCATCGAATCTTTGAACGCACATTGCGCCCTTTGGTATTCCGAA

GGGCATGCCTGTTCGAGCGTCATTATCACCCCTCAAGCCCCCGGCTTGGTGTTGGACGGCTTGGTTGACCGCA ATGTCGACCCCTCCTAAAGACAATGACAGCGCCCCGCGGCACCCCCGGTACACTGAGCTTTTCATCGAGCACG TATCGGACAAGGGTCCCCGGGACACGGTCTCTCTTTCCCACGCATCGCGGGAACATTTTTTCTGAAAGGTTGA CCTCGGATCA

>57.2.1c(2) cyngra *Exophiala*

GTTTATGATACCTAGTGTTGCTTCGGTAGGCCTGGTCTATCTGTTATAGACCTGCCGGGGGGCCGTAAGACGC CCGCCGGAGAGTGCCTACCGACAGCCTCAACTCCAAAATTCTTTAACCAAACGTGTCTTTGTCTGAGTAACGTC TTTTAAAATAAAGCAAAACTTTCAACAACGGATCTCTTGGTTCTGGCATCGATGAAGAACGCAGCGAAATGCG ATAAGTAATGCGAATTGCAGAATTCTCGTGAGTCATCGAATCTTTGAACGCACATTGCGCCCTTTGGTATTCCG AAGGGCATGCCTGTTCGAGCGTCATTTTCACCCCTCAAGCCCCCGGCTTGGTGTTGGACGGTCTGGTCCGGGG ACCTCAAACCCCCTGGACCCCTCCCAAAGACAATGACGGCGGGCTGTTGAACCCCCGGTACACTGAGCATCTT CACGGAGCACGTACCGGTCTCAAGGGTCGACGGCACCCGGTC

>56.1.6b(2) cynrid *Exophiala*

CCATTGTTTATGATACCTAGTGTTGCTTCGGTAGGCCTGGTCTATCTGTTATAGACCTGCCGGGGGGCCGTAA GACGCCCGCCGGAGAGTGCCTACCGACAGCCTCAACTCCAAAATTCTTTAACCAAACGTGTCTTTGTCTGAGTA ACGTCTTTTAAAATAAAGCAAAACTTTCAACAACGGATCTCTTGGTTCTGGCATCGATGAAGAACGCAGCGAA ATGCGATAAGTAATGCGAATTGCAGAATTCTCGTGAGTCATCGAATCTTTGAACGCACATTGCGCCCTTTGGTA TTCCGAAGGGCATGCCTGTTCGAGCGTCATTTTCACCCCTCAAGCCCCCGGCTTGGTGTTGGACGGTCTGGTCC GGGGACCTCAAACCCCCTGGACCCCTCCCAAAGACAATGACGGCGGGCTGTTGAACCCCCGGTACACTGAGC ATCTTCACGGAGCACGTACCGGTCTCAAGGGTCGACGGCACCCGGT

>56.1.4b(1) cynrid *Mollisia* A (1)(6) consensus

GCAGAGCTTAACGGAGACCTTGCACCCGAGAGGGGGGAGGCGACTATAAAAGAACTCCCAGCGCAAGTCAG CTTGGCTGGCAACACAATCGAATTGCGGGGACGCTTTAAAGCCTACCGGTACCAACTGCTCCTCTGGGGCAGG GCCAGCTACGCTGGAGGTCACAACCCGGTGAGATATTACAATAAGCAATCCGCAGCGGCTCAGCCGTCCACA GACTAAGTGGTTGTGGGTAGGGATCTCCTACTTAAGATATAGTCGGGCCCTGTGATAAGTCATAGGGGCTTCT GCGTCCGTAGGTGAACCTGCGGAAGGATCATTAATAAAAGGATACCTTCGGGTATACCCCATCCGTGTCTACA TACTCTTGTTGCTTTGGCAGGCCGTGGTCTCCCACTGTGGGCTCTGCCTGCATGTGCCTGCCAGAGGACCAAA CTCTGAATGTTAGTGATGTCTGAGTACTATATAATAGTTAAAACTTTCAACAACGGATCTCTTGGTTCTGGCAT CGATGAAGAACGCAGCGAAATGCGATAAGTAATGTGAATTGCAGAATTCAGTGAATCATCGAATCTTTGAAC GCACATTGCACCCGGTGGTATTCCGCCGGGTATGCCTGTTCGAGCGTCATTATAACCGCTCAAGCTTTGTCTTG GTGTCGGGGTTGCGAATTTTGCAGCCTCAGAGTCCAGTAGCGTCACCTGTAGGTCCTAAGCGTAGTAATTTCT CCTCGCTACAGAACCTGCCGGTGGATAGTGT

>56. cynrid Helotiales(MollisiaA?) 1.4b(3)/1.5c(4)/2.4a(7) consensus

TCTACATACTCTTGTTGCTTTGGCAGGCCGTGGGTCTCCCACTGTGGGCTCTGCCTGCATGTGCCTGCCAGAGG ACCAAACTCTGAATGTTAGTGATGTCTGAGTACTATATAATAGTTAAAACTTTCAACAACGGATCTCTTGGTTC TGGCATCGATGAAGAACGCAGCGAAATGCGATAAGTAATGTGAATTGCAGAATTCAGTGAATCATCGAATCT TTGAACGCACATTGCACCCGGTGGTATTCCGCCGGGTATGCCTGTTCGAGCGTCATTATAACCGCTCAAGCTTT GTCTTGGTGTCGGGGTTGCGAATTTTGCAGCCTCAGAGTCCAGTAGCGTCACCTGTAGGTCCTAAGCGTAGTA ATTTCTCCTCGCTACAGAACCTGCCGGTGGATAGTGTAAATCCAGTTAAGTCTGTGTgtcctgctattaaacccccaaa

tttttaaggtt

>57.3.1c(11) cyngra Acephala (Phialocephala A) 2.4b(15)/3.1c(11) consensus

GGCAACCGGTAGACCCCACCCGTGTCTCTCTACTCTTGTTGCTTTGGCAGGCCGTGGCCTCCACCGCGGGCTCT GCCTGCGTGTGCCTGCCAGAGGACCAAACTCTGAATTTTAGTGATGTCTGAGTACTATATAATAGTTAAAACTT TCAACAACGGATCTCTTGGTTCTGGCATCGATGAAGAACGCAGCGAAATGCGATAAGTAATGTGAATTGCAG AATTCAGTGAATCATCGAATCTTTGAACGCACATTGCGCCCGGTGGTATTCCGCCGGGCATGCCTGTTCGAGC GTCATTATAACCACTCAAGCCTGGCTTGGTATTGGGGTTCGCGGTTCCGCGGCCCCTAAAATCAGTGGCGGTG CCGGTGGGCTCTAAGCGTAGTAAATCTCCTCGCTATAGGGTCCCCTCGGTTGCCTGCCAGAACCCCCCATTTTT TAGGTTGACCTCGGATCAG

>222.1.1g(6) cynhyb *Phialocephala* C

ACCAGCGGAGGGATCATTACAGGACTCGCAAGACTCCTTACACCCTGTGAACCTTACAGTATTACGCGTTGCT TCGGCGGGTCCGCCCGCCGGCAGCATACCACATTCTGTTTCTCTGCGTTGGCATCTCGAGAAAAAAGCAAATA AGTTAAAACTTTCAACAACGGATCTCTTGGTTCTGGCATCGATGAAGAACGCAGCGAAATGCGATAAGTAATG CGAATTGCAGAATTCCGTGAGTCATCGAATCTTTGAACGCACATTGCGCCCGCCAGTATTCTGGCGGGCATGC CTGTTCGAGCGTCATTGCAACCCCTCAGGCCCTGCCTGGCGTTGGAGGACTGCGCTACGCAGCCTCCCAAAGC AAGCGGCGGCGGCGCCCCAAATCCGAACGCAGTAGTTACCTCTCGTTCTGGGTGCTGGGACGGCGTCCGGCC GGAAAACCCCCATCTATATGGTG

>56.1.6b(20) cynrid *Phialocephala* A

CCCACCCGTGTCTATCTACTCTTGTTGCTTTGGCAGGCCGTGGCCTCCACCGCGGGCTCTGCCTGCGTGTGCCT GCCAGAGGACCAAACTCTGAATTTTAGTGATGTCTGAGTACTATATAATAGTTAAAAACTTTCAACAACGGATC TCTTGGTTCTGGCATCGATGAAGAACGCAGCGAAATGCGATAAGTAATGTGAATTGCAGAATTCAGTGAATCA TCGAATCTTTGAACGCACATTGCGCCCGGTGGTATTCCGCCGGGCATGCCTGTTCGAGCGTCATTATAACCACT CAAGCCTGGCTTGGTATTGGGGTTCGCGGTTTCCGCGGCCCCTAAAATCAGTGGCGGTG

>73.1.5b(14) cynpap *Phialocephala* B

CCTGCGGAAGGATCATTAAAAAGGATACCGGGAAACCGGTAGACCCCACCCGTGTCTATCTACTCTCGTTGCT TTGGCAGGCCGTGGCCTCCACCGCGGGCTCTGCCTGCGTGTGCCTGCCAGAGGACCAAACTCTGAATTTTGGT GATGTCTGAGTACTATATAATAGTTAAAAACTTTCAACAACGGATCTCTTGGTTCTGGCATCGATGAAGAACGC AGCGAAATGCGATAAGTAATGTGAATTGCAGAATTCAGTGAATCATCGAATCTTTGAACGCACATTGCGCCCG GTGGTATTCCGCCGGGCATGCCTGTTCGAGCGTCATTATAACCACTCAAGCCTGGCTTGGTATTGGGGTTCGC GGTTTCCGCGGCCCCTAAAATTAGTGGCGGTGCCGGTGGGCTCTAAGCGTAGTAAATCTCCTCGCTATAGGGT CCCTCCGGTTGCCTGCCAGAACCCCCCATTTTTTCA

>71.1.1b(2) cyngib *Arcopilus* A1

AGCGGAGGGATCATTACAGAGTTGCAAAACTCCCTAAACCATTGTGGACGCTACCTTTAACGTTGCTTCGGCG GGCGGCCCGCTCCCCTGGAAAGCCCCTGTGGCCGCCCGGGGCTGCGAGCCCCCGGCCCCCCTCGCGGGGGC GCCCGCCGGAGGATACCCAACTCTTGATTATTTTAGGCCTCTCTGAGTCTTCTGTACTGAATAAGTCAAAACTT TCAACAACGGATCTCTTGGTTCTGGCATCGATGAAGAACGCAGCGAAATGCGATAAGTAATGTGAATTGCAG AATTCAGTGAATCATCGAATCTTTGAACGCACATTGCGCCCGCCAGTATTCTGGCGGGCATGCCTGTTCGAGC GTCATTTCAACCATCAAGCCCCGGGCTTGTGTTGGGGACCTGCGGCTGCCGCAGGCCCTGAAATCCAGTGGCG GGTTCGCTGTCACCCCGAGCGTAGTAGCAATATCTCGCTCAGGGCGTGCTGCGGGCGCCGGCCGTTAAAAGC TGCCTTCTGGCAACACCCAAGGTTGACCTCGGATC

>222.1.1g cynhyb *Arcopilus* A2 (2)(7)(8) consensus

CAGCGGAGGGATCATTACAGAGTTGCAAAACTCCCTAAACCATTGTGGACGCTACCTTTTAACGTTGCTTCGGC GGGCGGCCCGCTCCCCTGGAAAGCCCCTGTGGCCGCCCGGGGCTGCGAGCCCCCCGGCCCCCCTCGCGGGG GCGCCCGCCGGAGGATACCCAACTCTTGATTATTTTAGGCCTCTCTGAGTCTTCTGTACTGAATAAGTCAAAAC

TTTCAACAACGGATCTCTTGGTTCTGGCATCGATGAAGAACGCAGCGAAATGCGATAAGTAATGTGAATTGCA GAATTCAGTGAATCATCGAATCTTTGAACGCACATTGCGCCCGCCAGTATTCTGGCGGGCATGCCTGTTCGAG CGTCATTTCAACCATCAAGCCCCAGGCTTGTGTTGGGGACCTGCGGCTGCCGCAGGCCCTGAAATCCAGTGGC GGGTTCGCTGTCACCCCGAGCGTAGTAGCAATATCTCGCTCAGGGCGTGCTGCGGGCGCCGGCCGTTAAAAG CTGCCTTCTGGCAACACCCAAGGTTGAC

>56.1.6b(3) cynrid *Arcopilus* B

CCTTCAAACGTTGCTTCGGCGGGCGGCCCGCTCCCCTGGAAAGCCCCTGTGGCCGCCCGGGGCTGCAGGCCC CCCGGCCCCCCTCGCGGGGGCGCCCGCCGGAGGATACCCAACTCTTGATAATTTTAGGCCTCTCTGAGTCTTCT GTACTGAATAAGTCAAAACTTTCAACAACGGATCTCTTGGTTCTGGCATCGATGAAGAACGCAGCGAAATGCG ATAAGTAATGTGAATTGCAGAATTCAGTGAATCATCGAATCTTTGAACGCACATTGCGCCCGCCAGTATTCTG GCGGGCATGCCTGTTCGAGCGTCATTTCAACCATCAAGCCCCAGGCTTGTGTTGGGGACCTGCGGCTGCCCGC AGGCCCTGAAAACCAGTGGCGGGCTCGCTGTCACCCCGAGCGTAGTAGCAATCACCTCGCTCAGGGCGTGCC GCGGGCGCCG

>57.2.2e(9) cyngra *Dichotomopilus*

CCAAACCATCGTGAACGTTACCTATATCGTTGCTTCGGCGGGTGGCTCGGCCTCGGCCCTGCCCTAGGCCCCTC TCGGGGGCCCGCCGGAGGTCTACCAAACTCTTGAACTTATATGGCCTCTCTGAGTCTTCTGTACTGAATAAGTC AAAACTTTCAACAACGGATCTCTTGGTTCTGGCATCGATGAAGAACGCAGCGAAATGCGATAAGTAATGTGAA TTGCAGAATTCAGTGAATCATCGAATCTTTGAACGCACATTGCGCCCGCCAGTATTCTGGCGGGCATGCCTGTC CGAGCGTCATTTCAACCATCAAGCCCCCGGCTTGTGTTGGGGACCTGCGGCACACCCGCAGGCCCTGAAAACC AGTGGCGGGCTCGCTGTCCACACCGAGCGTAGTAGCATATCTTTGTCTCGCTCAGGGCGTGCGGCGGGCCCC GGCCGTGAAACCCACCTTCTCGAAGGTACCCAAAGGTTGACCTCG

>57.2.4b(13) cyngra *Chaetomium* A

ACGTTGCTTCGGCGGGCGGCCCGCTCCCCTGGCGCCCATCTGCGGCGCCCGGGGCGCAGGCCCCCAGGCCCC CCCCGCGGGGGCGCCCGCCGGAGGTACCCAACTCTTGACAATTTTAGGCCTCTCTGAGTCTTCTGTACTGAAT AAGTCAAAACTTTCAACAACGGATCTCTTGGTTCTGGCATCGATGAAGAACGCAGCGAAATGCGATAAGTAAT GTGAATTGCAGAATTCAGTGAATCATCGAATCTTTGAACGCACATTGCGCCCGCCAGTATTCTGGCGGGCATG CCTGTTCGAGCGTCATTTCAACCATCAAGCCCCCGGCTTGTGTTGGGGACCTGCGGCTGCCCGCAGGCCCTGA AAACCAGTGGCGGGCTCGCTGTCACCCCGAGCGTAGTAGCAATCACCTCGCTCAGGGCGTGCCGCGGGCGCC GGCCGTTAAAGCTGCCTTC

>217.1.1f(3) cynfle *Chaetomium* B1

GAACCAGCGGAGGGATCATTACAGAGTTGCAAAACTCCCTAAACCATTGTGAACGTTACCTAAACCGTTGCTT CGGCGGGCGGCGCCGGGGTTTACCCCCCGGGCGCCCCTGGGCCCCACCGCGGGCGCCCGCCGGAGGTCACC AAACTCTTGATAATTTATGGCCTCTCTGAGTCTTCTGTACTGAATAAGTCAAAACTTTCAACAACGGATCTCTTG GTTCTGGCATCGATGAAGAACGCAGCGAAATGCGATAAGTAATGTGAATTGCAGAATTCAGTGAATCATCGA ATCTTTGAACGCACATTGCGCCCGCCAGTATTCTGGCGGGCATGCCTGTTCGAGCGTCATTTCAACCATCAAGC CCCGGGCTTGTGTTGGGGACCTGCGGCTGCCGCAGGCCCTGAAAAGCAGTGGCGGGCTCGCTGTCACACCGA GCGTAGTAGCATACATCTCGCTCTGGGCGTGCTGCGGGTTCCGGCCGTTAAACCACCTTTTAACCCAAGGTTG ACCTCGGATCAGGTAGGAA

>217.1.5b cynfle *Chaetomium* B2 (6)(9)(10) consensus

TTGGTGAACCAGCGGAGGGATCATTACAGAGTTGCAAAACTCCCTAAACCATTGTGAACGTTACCTAAACCGT TGCTTCGGCGGGCGGCCCCGGGGTTTACCCCCCGGGCGCCCCTGGGCCCCACCGCGGGCGCCCGCCGGAGGT CACCAAACTCTTGATAATTTATGGCCTCTCTGAGTCTTCTGTACTGAATAAGTCAAAACTTTCAACAACGGATCT

CTTGGTTCTGGCATCGATGAAGAACGCAGCGAAATGCGATAAGTAATGTGAATTGCAGAATTCAGTGAATCAT CGAATCTTTGAACGCACATTGCGCCCGCCAGTATTCTGGCGGGCATGCCTGTTCGAGCGTCATTTCAACCATCA AGCCCCGGGCTTGTGTTGGGGACCTGCGGCTGCCGCAGGCCCTGAAAAGCAGTGGCGGGCTCGCTGTCACAC CGAGCGTAGTAGCATACATCTCGCTCTGGGCGTGCTGCGGGTTCCGGCCGTTAAACCACCTTTTAACCCAAGG TTGACCTCG

>217.1 cynfle *Chrysanthotrichum* 2b(7)(16)(18)/4b(1) consensus

GTGAACCAGCGGAGGGATCATTAAAGAGTTGCAAAACTCCCAAACCATTGTGGACCTACCTTACAACCGTTGC TTCGGCGGGCGGCGCCGCCCCGGGGGGAGACCCCCGCGGCGCGCCCCTCGGCCCCTCACCGGGCGCCCGCC GGAGGTACCTAACCCTTTACGCATGTATGGCCTCTCTGAGTCTTCTGTACTGAATAAGTCAAAACTTTCAACAA CGGATCTCTTGGTTCTGGCATCGATGAAGAACGCAGCGAAATGCGATAAGTAATGTGAATTGCAGAATTCAGT GAATCATCGAATCTTTGAACGCACATTGCGCCCGCCAGCATTCTGGCGGGCATGCCTGTCCGAGCGTCATTTC AACCATCAAGCCCCCGGGCTTGTGCTGGGGACCTGCGGCTGCCCGCAGGCCCTGAAAACCAGTGGCGGGCTC GCTGTCACCCCGAGCGTAGTAGCATAACCTCGCTTAGGGCGTGCCGCGGGCTCCCGCCGTAAAACCCCCAAAA CACCAAAGGTTGACCTCGGATCAG

>56.2.1c(1) cynrid *Colletotrichum*

CGGAGGGATCATTATCGAGTTACCACTCTATAACCCTTTGTGAACATACCTACATGTTGCTTCGGCGGTCGGCC CCCCGGGCCCCCGGCCCCGCTCACGCGGGGCGTCCGCCGGAGGATAACCAAACTCTGATTTAACGACGTTTCT TCTGAGTGGCACAAGCAAATAATCAAAACTTTTAACAACGGATCTCTTGGTTCTGGCATCGATGAAGAACGCA GCGAAATGCGATAAGTAATGTGAATTGCAGAATTCAGTGAATCATCGAATCTTTGAACGCACATTGCGCCCGC CAGCATTCTGGCGGGCATGCCTGTTCGAGCGTCATTTCAACCCTCAAGCACTGCTTGGTGTTGGGGCTCTACG GTTGACGTAGGCCCCCAAAACTAGTGGCGGACCCTCTCGGAGCCTCCTTTGCGTAGTAACTTTTGTCTCGCACT GGGATTCGGAGGGATTCTAGCCGTTAAACCCCCAATTTTCTAAAGGT

>222.1.1k(15) cynhyb *Pestalotiopsis* A

AGGGATCATTATAGAGTTTTCTAAACTCCCAACCCATGTGAACTTACCATTGTTGCCTCGGCAGAAGCTGCTCG GTGCACCCTACCTTGGAACGGCCTACCCTGTAGCGCCGTTACCCTGGAACGGCTTACCCTGTAACGGCTGCCG GTGGACTACCAAACTCTTGTTATTTTATTGTAATCTGAGCGTCTTATTTTAATAAGTCAAAACTTTCAACAACGG ATCTCTTGGTTCTGGCATCGATGAAGAACGCAGCGAAATGCGATAAGTAATGTGAATTGCAGAATTCAGTGAA TCATCGAATCTTTGAACGCACATTGCGCCCATTAGTATTCTAGTGGGCATGCCTGTTCGAGCGTCATTTCAACC CTTAAGCCTAGCTTAGTGTTGGGAGCCTACTGCTTTTGCTAGCTGTAGCTCCTGAAATACAACGGCGGATCTGC GATATCCTCTGAGCGTAGTAAATTTTTATCTCGCTTTTGACTGGAGTTGCAGCGTCTTTGGCCGCTAAATCCCCC AAT

>56.2.2c(4) cynrid/57.3.1c(7) cyngra *Pestalotiopsis* B

GGAGGATCATTATAGAGTTTTCTAAACTCCCAACCCATGTGAACTTACCATTGTTGCCTCGGCAGAAGCTACCT GGTGCACCTTACCCTGGAACGGCCTACCCTGTAGCGCCTTACCCTGGAACGGCTTACCCTGTAGCGGCTGCCG GTGGACTACCAAACTCTTGTTATTTTATTGTAATCTGAGCGTCTTATTTTAATAAGTCAAAACTTTCAACAACGG ATCTCTTGGTTCTGGCATCGATGAAGAACGCAGCGAAATGCGATAAGTAATGTGAATTGCAGAATTCAGTGAA TCATCGAATCTTTGAACGCACATTGCGCCCATTAGTATTCTAGTGGGCATGCCTGTTCGAGCGTCATTTCAACC CTTAAGCCTAGCTTAGTGTTGGGAGCCTACTGCTTTTGCTAGCGGTAGCTCCTGAAATACAACGGCGGATCTG CGATATCCTCTGAGCGTAGTAATTTTTATCTCGCTTTTGACTGGAGTTGCAGCGTCTTTAGCCGCTAAACCCCCC AATTTTTAATGGTTGACC

>222.1. cynhyb *Coniella* A consensus 1b(4)/3d(27)(28)/4c(34)(36)(38)/6d(1)

GGTGAACCAGCGGAGGGATCATTGCTGGAACTGTGTCTGCAAAGACACAACCCCAGATACCCTTTGTGAACTT ATTCTTATATCGTTGCCTCGGCGCTGAGCTGGGGGCTTCTTTTCCAAGGAGCTCTCCCATCCTCTCGGATGGAG CAAGCCCGCCGGCGGCCCTTCTAAACTCTTGTTTTTATTACGTATCTCTTCTGAGTTATTCAAAACAAAATGAAT CAAAACTTTCAACAACGGATCTCTTGGTTCTGGCATCGATGAAGAACGCAGCGAAATGCGATAAGTAATGTGA ATTGCAGAATTCAGTGAATCATCGAATCTTTGAACGCACATTGCGCCCGCTGGAATTCCAGCGGGCATGCCTG TTCGAGCGTCATTTCACCCCTCAAGCCTAGCTTGGTGTTGGAGCACTACCGCTTTCCCAAGCGGTAGGCTCTGA AATTCAGTGGCGGGCTCGCTAAGACTCTGAGCGTAGTAGTTTATCACCTCGCTTTGGAAGGATTAGCGGTGCT CTTGCCGTAAAACCCCCCAACTTCTGAATTTTGACCTCGGATCAGGT

>201.5.wp(25) cynfas *Coniella* B

TACCCTATGTGGAACTTATACCTTTTAATCGTTGCCTCGGCGTTAAGCTGGGGGCTTCTAGAAGCCCTCCCGTA TTCTTTCGAGAACACGGAGCAAGCCCGCCGGCGGCCTATTAAACTCTGTTTTTATTACGTATCTCTTCTGAGTT ATTCAAAACAAAATGAATCAAAACTTTCAACAACGGATCTCTTGGTTCTGGCATCGATGAAGAACGCAGCGAA ATGCGATAAGTAATGTGAATTGCAGAATTCAGTGAATCATCGAATCTTTGAACGCACATTGCGCCCGCTGGTA TTCCAGCGGGCATGCCTGTTCGAGCGTCATTTCACCCCTCAAGCCTAGCTTGGTGTTGGAGCACTACCGTGTTA CAGCGGTAGGCTCTGAAATTCAGTGGCGGGCTCGCTAAGACTCTGAGCGTAGTAGTTAATCACCTCGCTTTGG AAGAATTAGGCGGTGCTCTTGCCGTAAAACCCCCAACTTCTGAATTTGACCTCGATC

>222.1.1b cynhyb *Coniochaeta* (3)(4) consensus

GAACCAGCGGAGGGATCATTATTAGAAGCCGAAAGGCTACTTAAAACCATCGCGAACTCGTCCAAGTTGCTTC GGCGGCGCGGCCTCCCTCACGGGGGCGCCGCAGCCCCGCCTCTCCGGAGGTGTGGGGCGCCCGCCGGAGGT ACGAAACTCTGTATTATAGTGGCATCTCTGAGTAAAAAACAAATAAGTTAAAACTTTCAACAACGGATCTCTTG GTTCTGGCATCGATGAAGAACGCAGCGAAATGCGATAAGTAATGTGAATTGCAGAATTCAGTGAATCATCGA ATCTTTGAACGCACATTGCGCCCGCTAGTACTCTAGCGGGCATGCCTGTTCGAGCGTCATTTCAACCCTCAAGC CCTGCTTGGTGTTGGGGCCCTACGGCTGCCGTAGGCCCTGAAAGGAAGTGGCGGGCTCGCTACAACTCCGAG CGTAGTAATTCATTATCTCGCTAGGGACGTTGCGGCGCGCTCCTGCCGTTAAAGACCATCTTTAACTCAA

>221.1.4b(16) cynuni *Pseudophialophora*

AGGGATCATTATCGAGTTGCAAAACTCCAACCCCATGTGAACATACCTCAGTCGTTGCTTCGGCGGTTTAGCCC CTAAAAAGGGCCCAAAGCCGCCGGAGGTTCCAAACTCTTGTTTTTATCAGTGTATCTTCTGAGCTTTAAAACAA ATAATCAAAACTTTCAACAACGGATCTCTTGGTTCTGGCATCGATGAAGAACGCAGCGAAATGCGATAAGTAA TGTGAATTGCAGAATTCAGTGAATCATCGAATCTTTGAACGCACATTGCGCCCGCCGGTATTCCGGCGGGCAT GCCTGTTCGAGCGTCATTTCAACCCTCAAGCCCAGCTTGGTGTTGGGGCGCCCGGCCGCCTGGCGGTCCGGG GCCCTCAAGTGTATCGGCGGTCTCGTCGGGACTCTGAGCGCAGTAACTCGCGGTAAAACGCGCCTCGCTTGGT CTGTCTCCGGCGGGCTCCGGCCGCTAAACCCCC

>217.1.1f(2) cynfle *Fusarium*

AGGTCTCCGTTGGTGAACCAGCGGAGGGATCATTACCGAGTTTACAACTCCCAAACCCCTGTGAACATACCAC TTGTTGCCTCGGCGGATCAGCCCGCTCCCGGTAAAACGGGACGGCCCGCCAGAGGACCCCTAAACTCTGTTTC TATATGTAACTTCTGAGTAAAACCATAAATAAATCAAAACTTTCAACAACGGATCTCTTGGTTCTGGCATCGAT GAAGAACGCAGCAAAATGCGATAAGTAATGTGAATTGCAGAATTCAGTGAATCATCGAATCTTTGAACGCAC ATTGCGCCCGCCAGTATTCTGGCGGGCATGCCTGTTCGAGCGTCATTTCAACCCTCAAGCACAGCTTGGTGTT GGGACTCGCGTTAATTCGCGTTCCCCAAATTGATTGGCGGTCACGTCGAGCTTCCATAGCGTAGTAGTAAAAC CCTCGTTACTGGTAATCGTCGCGGCCACGCCGTTAAACCCCAACTTCTGAATGTTGACCTCGGATCAG

>56. cynrid *Fusarium* 1.6b(16)/2.1c(8)(9)/2.4a(6) consensus

GGATCATTACCGAGTTTACAACTCCCAAACCCCTGTGAACATACCACTTGTTGCCTCGGCGGATCAGCCCGCTC CCGGTAAAACGGGACGGCCCGCCAGAGGACCCCTAAACTCTGTTTCTATATGTAACTTCTGAGTAAAACCATA AATAAATCAAAACTTTCAACAACGGATCTCTTGGTTCTGGCATCGATGAAGAACGCAGCAAAATGCGATAAGT AATGTGAATTGCAGAATTCAGTGAATCATCGAATCTTTGAACGCACATTGCGCCCGCCAGTATTCTGGCGGGC ATGCCTGTTCGAGCGTCATTTCAACCCTCAAGCACAGCTTGGTGTTGGGACTCGCGTTAATTCGCGTTCCCCAA ATTGATTGGCGGTCACGTCGAGCTTCCATAGCGTAGTAGTAAAACCCTCGTTACTGGTAATCGTCGCGGCCAC GCCGTTAAACCCCAACTTCTGAATGTTGACCTCGG

>56.2.2c(2) cynrid/57.2.4b(5) cyngra *Myrothecium* consensus

AGCGGAGGGATCATTACCGAGTTTACAACTCCCAAACCCAATGTGAACATACCCCAATGTTGCCTCGGCGGGA CCGCCCCGGCGCCCTCACCGGCCCGGAACCAGGCGCCCGCCGCAGGACCCAAACCTCTGATTTACTTATGGAT TCTCCTCTGAGTGGATTTTACAAATAAATCAAAACTTTCAACAACGGATCTCTTGGCTCTGGCATCGATGAAGA ACGCAGCGAAATGCGATAAGTAATGTGAATTGCAGAATTCAGTGAATCATCGAATCTTTGAACGCACATTGCG CCCGCCAGCATTCTGGCGGGCATGCCTGTCCGAGCGTCATTTCAACCCTCAGGCTCCCGCGCCTGGCGTTGGG GATCGGCCTTCACCGGCCGGCCCCGAAATACAGTGGCGGCCCCGCCCGTGTACCTCTGCGTAGTAGCACAACC TCGCAGCTGGGAGCGGCGGCGGCCACGCCGGAAAACCCCCGACTTCTGAAAGTTGACCTCGGA

>56.1.6b(22) cynrid *Metarhizium* A1

TACCTTTAATTGTTGCTTCGGCGGGACTTCGCGCCCGCCGGGGACCCAAACCTTCTGAATTTTTAATAAGGATC TTCTGAGTGGTTAAAAAAATGAATCAAAACTTTCAACAACGGATCTCTTGGTTCTGGCATCGATGAAGAACGC AGCGAAATGCGATAAGTAATGTGAATTGCAGAATTCAGTGAATCATCGAATCTTTGAACGCACATTGCGCCCG TCAGTATTCTGGCGGGCATGCCTGTTCGAGCGTCATTACGCCCCTCAAGTCCCCTGTGGACTTGGTGTTGGGG ATCGGCGAGGCTGGTTTTCCAGCACAGCCGTCCCTTAAATGAATTGGCGGTCTCGCTGTGGCCCTCCTCTGCG CAGTAGTAAAACACTCGCAACAGGAGCCCGGCGCGGT

>73.1.5b(7) cynpap *Metarhizium* A2

CAGCGGAGGGATCATTACCGAGTTATCCAACTCCCAACCCCTGTGAATTATACCTTTAATTGTTGCTTCGGCGG GACTTCGCGCCCGCCGGGGACCCAAACCTTCTGAATTTTTAATAAGTATCTTCTGAGTGGTTAAAAAAATGAAT CAAAACTTTCAACAACGGATCTCTTGGTTCTGGCATCGATGAAGAACGCAGCGAAATGCGATAAGTAATGTGA ATTGCAGAATTCAGTGAATCATCGAATCTTTGAACGCACATTGCGCCCGTCAGTATTCTGGCGGGCATGCCTGT TCGAGCGTCATTACGCCCCTCAAGTCCCCTGTGGACTTGGTGTTGGGGATCGGCGAGGCTGGTTTTCCAGCAC AGCCGTCCCTTAAATTAATTGGCGGTCTCGCCGTGGCCCTCCTCTGCGCAGTAGTAAAACACTCGCAACAGGA GCCCGGCGCGGTCCACTGCCGTAAAACCCCCC

>58.1.1d(4) cynrid Sordariomycetes A

AGGGATCATTACCGAGTTTACAACTCCCAAACCCCTGTGAACATACCTATCGTTGCTTCGGCGGGATCGCCCCG GTGCCCTCGGGCCCGGATCCAGGCGCCCGCCGGAGGACCCAAACTCTTGTTTCTATGAGTATCTTCTGAGTAA CAAGCAAATAAATTAAAACTTTCAACAACGGATCTCTTGGTTCTGGCATCGATGAAGAACGCAGCGAAATGCG ATAAGTAATGTGAATTGCAGAATTCCGTGAATCATCGAATCTTTGAACGCACATTGCGCCCGCCAGTATTCTGG CGGGCATGCCTGTTCGAGCGTCATTTCAACCCTCAAGCCCCCGGGCTTGGTGTTGGGGCTCGGCCCGTCCCCT CGCGGCGCGCCGTCTCCGAAATCTAGTGGCGGTCTCGCTGTAGCCTCCTCTGCGTAGTAGCTAACACCTCGCA ACGGGAACGCAGCGCGGCCACGCCGTTAAACCCCCCACTTCTGAAGGTTGACC

>222.1.1k(12) cynhyb SordariomycetesB

GGATCATTAAAGAGTGTAAAAACTCCTTAAAACCATCGTGAACTCATCCCGTCAGCGTTGCTTCGGCGGGCGG CCCCAGGGGGGGTCGCGGCCCGGAACCGTTACCTGCCTCTCGGCGGGTCTCGGGGTACGGGCGCCCGCCGG AGGTATCAAAACTCTTCTGTAACACAGCGGCATCTCTGAGTAATACAAGCAAATAAATCAAAACTTTCAACAAC

GGATCTCTTGGTTCTGGCATCGATGAAGAACGCAGCGAAATGCGATAAGTAATGTGAATTGCAGAATTCAGT GAATCATCGAATCTTTGAACGCACATTGCGCCCGCTAGTATTCTAGCGGGCATGCCTGTTCGAGCGTCATTTCA ACCCTCAAGCACTGCTTGGTGTTGGGGCCCTACGGCTGCCGTAGGCCCTGAAAACGAGTGGCGGGCTCGCTG CAACTCCGAGCGTAGTAGAATCATACCACCTCGCTAGGGAGGCTGCGGCGGCGCTCCGGCCGTTAAAGACCC CATCTTTAACCC

>57.3.1c(10) cyngra SordariumycetesC (4)(5)(8)(10) consensus

ACTCCCTAAACACTTGTGAACCTACCCGTATCGTTGCTTCGGTGGGCGGCCCCAGGGCGGGGCCGTAGCCTTT ACAGGCGCCCACCGAAGGTTATAAACGCTATATTTTATTGTCGCTCTGAGTAAACTTTTAATAAGTTAAAACTT TCAACAACGGATCTCTTGGTTCTGGCATCGATGAAGAACGCAGCGAAATGCGATAAGTAATGTGAATTGCAG AATTCAGTGAATCATCGAATCTTTGAACGCACATTGCACCTACCAGTATTCTGGTAGGTATGCCTGTTCGAGCG TCATTTCAACCCTCAAGCTTTGCTTGGTGTTGGGGTCCTACGCCTCGCGCGTAGGCCCTTAAGACTAGTGGCG GACCTTTTGTGATCCCGAGCGTAGTAATTATTACCGCTTTGGAGAGCTGGAGGGACCTAGCCGTTAAACCCCA TATTCTCAAGGTT

>56.1.6b(23) cynrid Hypocreales

ACCCGAACGTTGCCTCGGCGGGACCGCCCCGGCGCCCACAGCGGCCCGGAACCAGGCGCCCGCCGGAGGAC CCAAACTCTTGCTTTAAACAGTGGCATACTCTCTGAGTCTCACAAACAAAAAATAAATCAAAACTTTCAACAAC GGATCTCTTGGCTCTGGCATCGATGAAGAACGCAGCGAAATGCGATAAGTAATGTGAATTGCAGAATTCAGT GAATCATCGAATCTTTGAACGCACATTGCGCCCGCCAGCATTCTGGCGGGCATGCCTGTCCGAGCGTCATTTC AACCCTCAGGGAGCCCCCTCGCGGGGGGGACCTGGTGTTGGGGGCCGGCCGCCCAGCGCGCGCCGCCCCCG AAATGCAGTGGCGACCTCGCCGCAGCCTCCCCTGCGTAGTAGCACAACCTCGCACCGGAGCGCGGAGACGGT CACGCCGTAAAACGCCCAACTTTCAAGAGTT

>57.3.1c(3) cyngra *Acremonium persicinum*

GTTGCTTCGGCGGGACCGCCCCGGGCGCCTTCTTGGTGCCCCGGAATCAGGCGCCCGCCGGGGACACCAAAC TCTTGATTGTTATAGTGGCATTCTCTGAGTAAAACATACAAATAAGTCAAAACTTTCAACAACGGATCTCTTGG CTCTGGCATCGATGAAGAACGCAGCGAAATGCGATAAGTAATGCGAATTGCAGAATTCAGTGAATCATCGAA TCTTTGAACGCACATTGCGCCCGCTAGTATTCTGGCGGGCATGCCTGTCTGAGCGTCATTTCAACCCTCGCCCC CGGCTTTTGCTGGGAGCGGTGTTGGGGATCGGCCGCCCGTCATCTGGGAGGCCGGCCCCGAAATAGAGTGG CGACCACGCCGTGTGCTCCTCTGCGTAGTAGTAAATCACCTCGCAGGCGGACAGCGGTGCGGCCTGCCGTAA AACCCCCAACTC

>56.2.2c(1) cynrid Neocosmospora Fusarium neocosmosporiellum

GGATCATTACCGAGTTATACAACTCATCAACCCCTGTGAACTTACCTACAACGTTGCTTCGGCGGGAACAGAC GGCCCCGTAAAACGGGCCGCCCCCGCCAGAGGACCCCTAACTCTGTTTTTATAATGTTTTTCTGAGTAAACAAG CAAATAAATTAAAACTTTCAACAACGGATCTCTTGGCTCTGGCATCGATGAAGAACGCAGCGAAATGCGATAA GTAATGTGAATTGCAGAATTCAGTGAATCATCGAATCTTTGAACGCACATTGCGCCCGCCAGTATTCTGGCGG GCATGCCTGTTCGAGCGTCATTACAACCCTCGGGCCTCCGGGCCTGGCGTTGGGGATCGGCGGAGCCCCCCG TGGGCACACGCCGTCCCCCAAATACAGTGGCGGTCCCGCCGCAGCTTCCATTGCGTAGTAGCTAACACCTCGC AACTGGAGAGCGGCGCGGCCACGCCGTAAAACACCCAACTTTCTGAATGTTGACCTCGAATC

>56.1.1b(2) cynrid *Purpureocillium*

CGGAGGGATCATTACCGAGTTATACAACTCCCAAACCCACTGTGAACCTTACCTCAGTTGCCTCGGCGGGAAC GCCCCGGCCGCCTGCCCCCGCGCCGGCGCCGGACCCAGGCGCCCGCCGCAGGGACCCCAAACTCTCTTGCATT ACGCCCAGCGGGCGGAATTTCTTCTCTGAGTTGCACAAGCAAAAACAAATGAATCAAAACTTTCAACAACGGA TCTCTTGGTTCTGGCATCGATGAAGAACGCAGCGAAATGCGATAAGTAATGTGAATTGCAGAATTCAGTGAAT

CATCGAATCTTTGAACGCACATTGCGCCCGCCAGCATTCTGGCGGGCATGCCTGTTCGAGCGTCATTTCAACCC TCGAGCCCCCCCGGGGGCCTCGGTGTTGGGGGACGGCACACCAGCCGCCCCCGAAATGCAGTGGCGACCCC GCCGCAGCCTCCCCTGCGTAGTAGCACACACCTCGCACCGGAGCGCGGAGGCGGTCACGCCGTAAAACGCCC AACTTTCTTAGAGTTGACCT

>217.1.4c(4) cynfle *Penicillifer*

TTGGTGAACCAGCGGAGGGATCATTACCGAGTTTACAACTCCCAAACCCCTGTGAACCATACCATATCGTTGC CTCGGCGGCGCCCCCTCGGGGGACCGCCAGAGGACCCAAACTCTCTGCCTTTGATAAGTTTGTCTGAGTGTTG CTGCAAGCAAAATAAATCAAAACTTTCAACAACGGATCTCTTGGCTCTGGCATCGATGAAGAACGCAGCGAAA TGCGATACGTAATGTGAATTGCAGAATCCAGTGAATCATCGAATCTTTGAACGCACATTGCGCCCGCCAGTAT TCTGGCGGGCATGCCTGTCCGAGCGTCATTTCAACCCTCAAGCCCCCTCGGGTGCTTGGTGTTGGAGACCGGC GAATCCGGCCCCTCTCCGGGGTCCCGGGCGCCGCCTCCCAAATCCAGTGGCGGTCTCGCCGCAGCCTCCCCTG CGTAGTAGCATCTTATTTCGCATGGGAGAGCGGCGCGGCCACGCCGTTAAACACCCCACTTTAACGAACGGTT GACCTCGGATCAG

>222.1.1b(28) cynhyb *Trichoderma longibrachiatum* A

AGGGATCATTACCGAGTTTACAACTCCCACACCCCAATGTGAACGTTACCAATCTGTTGCCTCGGCGGGATTCT CTTGCCCCGGGCGCGTCGCAGCCCCGGATCCCATGGCGCCCGCCGGAGGACCAACTCCAAACTCTTTTTTCTCT CCGTCGCGGCTCCCGTCGCGGCTCTGTTTTATTTTTGCTCTGAGCCTTTCTCGGCGACCCTAGCGGGCGTCTCG AAAATGAATCAAAACTTTCAACAACGGATCTCTTGGTTCTGGCATCGATGAAGAACGCAGCGAAATGCGATAA GTAATGTGAATTGCAGAATTCAGTGAATCATCGAATCTTTGAACGCACATTGCGCCCGCCAGTATTCTGGCGG GCATGCCTGTCCGAGCGTCATTTCAACCCTCGAACCCCTCCGGGGGGTCGGCGTTGGGGATCGGCCCCTCACC GGGCCGCCCCCGAAATACAGTGGCGGTCTCGCCGCAGCCTCTCCTGCGCAGTAGTTTGCACACTCGCACCGGG AGCGCGGCGCGGCCACAGCCGTAAAACACCCCAAACTTCTGAAATGTTGACCTCG

>77.1.2c(1) cynspe *Trichoderma longibrachiatum*/*orientale* A 2b(5)(13)/2c(1)

TGGTGAACCAGCGGAAGGGATCATTACCGAGTTTACAACTCCCAAACCCCAATGTGAACGTTACCAATCTGTT GCCTCGGCGGGATTCTCTTGCCCCGGGCGCGTCGCAGCCCCGGATCCCATGGCGCCCGCCGGAGGACCAACT CCAAACTCTTTTCTCTCTCCGTCGCGGCTCCCGTCGCGGCTCTGTTTTATTTTTGCTCTGAGCCTTTCTCGGCGAC CCTAGCGGGCGTCTCGAAAATGAATCAAAACTTTCAACAACGGATCTCTTGGTTCTGGCATCGATGAAGAACG CAGCGAAATGCGATAAGTAATGTGAATTGCAGAATTCAGTGAATCATCGAATCTTTGAACGCACATTGCGCCC GCCAGTATTCTGGCGGGCATGCCTGTCCGAGCGTCATTTCAACCCTCGAACCCCTCCGGGGGGTCGGCGTTGG GGATCGGCCCCTCACCGGGCCGCCCCCGAAATACAGTGGCGGTCTCGCCGCAGCCTCTCATGCGCAGTAGTTT GCACACTCGCACCGGGAGCGCGGCGCGGCCACAGCCGTAAAACACCCCAAATTTTCTGAAATGTTGACCTCG GATCAGGTAG

>71.1.1b(1)/67.1.1a(12)(13) cyngib *Trichoderma* B consensus

GGTGAACCAGCGGAGGGATCATTACCGAGTTTACAACTCCCAAACCCAATGTGAACGTTACCAAACTGTTGCC TCGGCGGGATCTCTGCCCCGGGTGCGTCGCAGCCCCGGACCAAGGCGCCCGCCGGAGGACCAACCAAAACTC TTATTGTATACCCCCTCGCGGGTTTTTTTATAATCTGAGCCTTCTCGGCGCCTCTCGTAGGCGTTTCGAAAATGA ATCAAAACTTTCAACAACGGATCTCTTGGTTCTGGCATCGATGAAGAACGCAGCGAAATGCGATAAGTAATGT GAATTGCAGAATTCAGTGAATCATCGAATCTTTGAACGCACATTGCGCCCGCCAGTATTCTGGCGGGCATGCC TGTCCGAGCGTCATTTCAACCCTCGAACCCCTCCGGGGGGTCGGCGTTGGGGATCGGCCCTCCCTTAGCGGGT GGCCGTCTCCGAAATACAGTGGCGGTCTCGCCGCAGCCTCTCCTGCGCAGTAGTTTGCACACTCGCATCGGGA GCGCGGCGCGTCCACAGCCGTTAAACACCCAACTTCTGAAATGTTGACCTCGGATCAGG

>217.1.1c(12)/2b(9)cynfle/221.1.1b(52)/4b(36)cynuni *Trichoderma* C1 consensus

CCGTTGGTGAACCAGCGGAGGGATCATTACCGAGTTTACAACTCCCAAACCCAATGTGAACCATACCAAACTG TTGCCTCGGCGGGGTCACGCCCCGGGTGCGTCGCAGCCCCGGAACCAGGCGCCCGCCGGAGGGACCAACCA AACTCTTTCTGTAGTCCCCTCGCGGACGTTATTTCTTACAGCTCTGAGCAAAAATTCAAAATGAATCAAAACTTT CAACAACGGATCTCTTGGTTCTGGCATCGATGAAGAACGCAGCGAAATGCGATAAGTAATGTGAATTGCAGA ATTCAGTGAATCATCGAATCTTTGAACGCACATTGCGCCCGCCAGTATTCTGGCGGGCATGCCTGTCCGAGCG TCATTTCAACCCTCGAACCCCTCCGGGGGGTCGGCGTTGGGGATCGGGAACCCCTAAGACGGGATCCCGGCC CCGAAATACAGTGGCGGTCTCGCCGCAGCCTCTCCTGCGCAGTAGTTTGCACAACTCGCACCGGGAGCGCGG CGCGTCCACGTCCGTAAAACACCCAACTTCTGAAATGTT

>222.1 cynhyb *Trichoderma* C2 1b(16)/1k(5)

AGGGATCATTACCGAGTTTACAACTCCCAAACCCAATGTGAACCATACCAAACTGTTGCCTCGGCGGGGTCAC GCCCCGGGTGCGTCGCAGCCCCGGAACCAGGCGCCCGCCGGAGGGACCAACCAAACTCTTTCTGTAGTCCCC TCGCGGACGTTATTTTTACAGCTCTGAGCAAAAATTCAAAATGAATCAAAACTTTCAACAACGGATCTCTTGGT TCTGGCATCGATGAAGAACGCAGCGAAATGCGATAAGTAATGTGAATTGCAGAATTCAGTGAATCATCGAAT CTTTGAACGCACATTGCGCCCGCCAGTATTCTGGCGGGCATGCCTGTCCGAGCGTCATTTCAACCCTCGAACCC CTCCGGGGGGTCGGCGTTGGGGATCGGGAACCCCTAAGACGGGATCCCGGCCCCGAAATACAGTGGCGGTC TCGCCGCAGCCTCTCATGCGCAGTAGTTTGCACAACTCGCACCGGGAGCGCGGCGCGTCCACGTCCGTAAAAC ACCCAACTTCT

>201 cynfas *Trichoderma* D 2.wp(1)/5.wp(44)

GCGGAGGGATCATTACCGAGTTTACAACTCCCAAACCCAATGTGAACCATACCAAACTGTTGCCTCGGCGGGG TCACGCCCCGGGTGCGTCGCAGCCCCGGAACCAGGCGCCCGCCGGAGGGACCAACCAAACTCTTTTCTGTAG TCCCCTCGCGGACGTTATTTCTTACAGCTCTGAGCAAAAATTCAAAATGAATCAAAACTTTCAACAACGGATCT CTTGGTTCTGGCATCGATGAAGAACGCAGCGAAATGCGATAAGTAATGTGAATTGCAGAATTCAGTGAATCAT CGAATCTTTGAACGCACATTGCGCCCGCCAGTATTCTGGCGGGCATGCCTGTCCGAGCGTCATTTCAACCCTCG AACCCCTCCGGGGGGTCGGCGTTGGGGACCTCGGGAGCCCCTAAGACGGGATCCCGGCCCCGAAATACAGT GGCGGTCTCGCCGCAGCCTCTCCTGCGCAGTAGTTTGCACAACTCGCACCGGGAGCGCGGCGCGTCCACGTC CGTAAAACACCCAACTTCTGAAATGTTGACCTCGGATCA

>222.1.3d cynhyb *Neopestalotiopsis* A1 consensus (6)(44)

GGTGAACCAGCGGAGGGATCATTATAGAGTTTTCTAAACTCCCAACCCATGTGAACTTACCTTTTGTTGCCTCG GCAGAAGTTATAGGTCTTCTTATAGCTGCTGCCGGTGGACCATTAAACTCTTGTTATTTTATGTAATCTGAGCG TCTTATTTTAATAAGTCAAAACTTTCAACAACGGATCTCTTGGTTCTGGCATCGATGAAGAACGCAGCGAAATG CGATAAGTAATGTGAATTGCAGAATTCAGTGAATCATCGAATCTTTGAACGCACATTGCGCCCATTAGTATTCT AGTGGGCATGCCTGTTCGAGCGTCATTTCAACCCTTAAGCCTAGCTTAGTGTTGGGAATCTACTTCTTTTATTA GTTGTAGTTCCTGAAATACAACGGCGGATTTGTAGTATCCTCTGAGCGTAGTAATTTTTTTTCTCGCTTTTGTTA GGTGCTATAACTCCCAGCCGCTAAACCCCCAATTTTTTGTGGTTGACCTCGGATCAG

>77.1.2d cynspe *Neopestalotiopsis* A2 consensus (1)(3)

GGTGAACCAGCGGAGGGATCATTATAGAGTTTTCTAAACTCCCAACCCATGTGAACTTACCTTTTGTTGCCTCG GCAGAAGTTATAGGTCTTCTTATAGCTGCTGCCGGTGGACCATTAAACTCTTGTTATTTTATGTAATCTGAGCG TCTTATTTTAATAAGTCAAAACTTTCAACAACGGATCTCTTGGTTCTGGCATCGATGAAGAACGCAGCGAAATG CGATAAGTAATGTGAATTGCAGAATTCAGTGAATCATCGAATCTTTGAACGCACATTGCGCCCATTAGTATTCT AGTGGGCATGCCTGTTCGAGCGTCATTTCAACCCTTAAGCCTAGCTTAGTGTTGGGAATCTACTTCTCTTAGGA GTTGTAGTTCCTGAAATACAACGGCGGATTTGTAGTATCCTCTGAGCGTAGTAATTTTTTTCTCGCTTTTGTTAG GTGCTATAACTCCCAGCCGCTAAACCCCCAATTTTTTGTGGTTGACCTCGGATC

>57.2.2e cyngra *Nigrospora* 1 (4)(5) consensus

GTGAACTTATCTCTTTGTTGCCTCGGCGCAAGCTACCCGGGACCCAGCGCCCCGGGCGGCCCGCCGGCGGAC AAACCAAACTCTTGTTATTCAATGGATTATCTGAGCGTCTTATTTAATAAGTCAAAACTTTCAACAACGGATCTC TTGGTTCTGGCATCGATGAAGAACGCAGCGAAATGCGATAAGTAATGTGAATTGCAGAATTCAGTGAATCATC GAATCTTTGAACGCACATTGCGCCCATCAGTATTCTGGTGGGCATGCCTGTTCGAGCGTCATTTCAACCCCTAA GCACAGCTTACTGTTGGGACTCTACGGCCTCCGTAGTTCCCCAAAGCCATTGGCGGAGTGGCAGTAGTCCTCT GAGCGTAGTAATTCTTTATCTCGCTTTTGTTAGGTGCTGCCCCCCCGGCCGTTAAACCCCCCAATTTTTTCTGGT

TGACCTCGGATCAG

>56.2.2c(7) cynrid *Nigrospora* 1

CCCAGTGAACTTATCTCTTTGTTGCCTCGGCGCAAGCTACCCGGGCACCCAGCGCCCCGGGCGGCCCGCCGGC GGACAAACCAAACTCTTGTTATTCAATGGATTATCATGAGCGTCTTATTTAATAAGTCAAAACTTTCAACAACG GATCTCTTGGTTCTGGCATCGATGAAGAACGCAGCGAAATGCGATAAGTAATGTGAATTGCAGAATTCAGTG AATCATCGAATCTTTGAACGCACATTGCGCCCATCAGTATTCTGGTGGGCATGCCTGTTCGAGCGTCATTTCAA CCCCTAAGCACAGCTTACTGTTGGGACTCTACGGCCTCGCGTA

>56.1.2b(9) cynrid *Nigrospora* 2

TCTTAGTTGATTATCTGAGTGTCTTATTTAATAAGTCAAAACTTTCAACAACGGATCTCTTGGTTCTGGCATCGA TGAAGAACGCAGCGAAATGCGATAAGTAATGTGAATTGCAGAATTCAGTGAATCATCGAATCTTTGAACGCA CATTGCGCCCATTAGTATTCTAGTGGGCATGCCTGTTCGAGCGTCATTTCAACCCCTAAGCACAGCTTATTGTT GGGAACCTACGGCTTCGTAGTTCCTCAAAGACATTGGCGGAGTGGCAGTGGTCCTCTGAGCGTAGTAATCTTT TATCTCGCTTCTGTTAGGTGCTGCCCCCCCGGCCGTAAAACCCCCAA

> 77.1.2b(3) cynspe *Xylaria*

GAACCAGCGGAGGGATCATTAAAGAGTTTTTACAACTCCCAAACCCCTGTGAACATACCTTCTGTTGCCTCGGC AGGCCCTGGCCTACCCTGTAGCGCCCCTACACTGTAGGGCTTGCTTTAGGGGGAGCGTTGGGGGCACCTGCC GGCGGCTCGCAAAACTCTGTTTAGCATTGAATTCTGAACATATAACTAAATAAGTTAAAACTTTCAACAACGGA TCTCTTGGTTCTGGCATCGATGAAGAACGCAGCGAAATGCGATAAGTAATGTGAATTGCAGAATTCAGTGAAT CATCGAATCTTTGAACGCACATTGCGCCCATTAGTATTCTAGTGGGCATGCCTGTTCGAGCGTCATTTCAACCC TTAAGCCCCTGTTGCTTAGCGTTGGGAGCCTACGGCAGCGTAGCTCCCTAAAGTTAGTGGCGTGGTCGGTTCA CACTCCAGACGTAGTAGATTTTCACCTCGCCTGTAGATGGATCGGTCCCCTGCCGTAAAACACCCCAATTTTTA AAGGTTGACCT

> 77.1.2b(12) cynspe *Xylaria feejeensis*

GTGAACCAGCGGAGGGATCATTAAAGAGTTTATAACTCCCAAACCCCATGTGAACATACCTAACGTTGCCTCG GCGGGTCGTACCTACCCTGTAGTGCACTTACCTGTAAGTGCCTACCCGGTAGGCACGGGTAAGCCCGCCGGC GCCCCATTAAACTCTGTTTAATTACTGGATATCTGAATTACAACTAAATAAGTTAAAACTTTCAACAACGGATCT CTTGGTTCTGGCATCGATGAAGAACGCAGCGAAATGCGATAAGTAATGTGAATTGCAGAATTCAGTGAATCAT CGAATCTTTGAACGCACATTGCGCCCATTAGTATTCTAGTGGGCATGCCTGTTCGAGCGTCATTTCAACCCTTA AGCCTTCTGTTGCTTAGCGTTGGGGGCCTACCGTATGGCGGTAGCCCCTTAAAATTAGTGGCGGAGTCGGTTC ACACTCTAGACGTAGTAAATATTATCTCGCCTATTAGTTGGACCGGTCCCCTGCCGTAAAACCCCTAATTTAT

> 77.1.2d(4) *cynspe Xylaria curta*

AAAGAGTTCTATAACTCCCAAACCCATGTGAACATACCGATACGCTTGCCTCGGCAGGATCGCGTCTACCCTGT AGCACCCCTACCCTGTAAGACCCTACCCTGGTAGGACTTCCCTGGTAGGACCTACCCGGCAGACGCGGGTAAG CCTGCCGGCGGCCCACGAAAACTCTGTTTTAGCATTGTACTTCTGAATACTATAACTAAATCAGTTAAAACTTTC AACAACGGATCTCTTGGTTCTGGCATCGATGAAGAACGCAGCGAAATGCGATAAGTAATGTGAATTGCAGAA

TTCAGTGAATCATCGAATCTTTGAACGCACATTGCGCCCATTAGTATTCTAGTGGGCATGCCTGTTCGAGCGTC ATTTCAACCCTTAAGCCTCTGTTGCTTAGTGTTGGGAGCCTACAGTACTGTAGCTCCTCAAAGTTAGTGGCGGA GTCGGCTCACAACTCTAGACGTAGTAATTTTTTTTCCCTCGCCTGTGTGTTGTGCCGGTCCCCTGCCGTAAAACC CCCTAATTTT

>201.2.wp(9) cynfas *Anthostomella*

GTTGGTGAACCAGCGGAGGGATCATTAGAGAGTATAAAAACTCCCAACCCATGTGAACATACCTACGTTGCCT CGGCGGGGGGCCTAAGAACCCCCCGCCGCTGGCCCAGCAAACTCTGTTTTATCTTGAAATTCTGAATATTAAA GCAAATAAGTTAAAACTTTCAACAACGGATCTCTTGGTTCTGGCATCGATGAAGAACGCAGCGAAATGCGATA AGTAATGTGAATTGCAGAATTCAGTGAATCATCGAATCTTTGAACGCACATTGCGCCCATTAGTATTCTAGTGG GCATGCCTGTTCGAGCGTCATTAAATCCATTAAGCCCTGTCGCTTAGCGTTGGGAGCCTGCGGCTGCAGCTCC TCAAAATTAGTGGCGGAGTCAGCGTATACTCTAGGCGTAGTAATTAACCACTCGCTTCTGTGTTGCGCCGGCG CCTGCCGTAAAACCCCCCATTTCTAAAGGTTTGACCTCGGATCAG

>58.1.1d cynrid fungusA (1)(3) consensus

GGTGAACCTGCGGAAGGATCATTACCGAGAACCGGCGCCCTCGTGGCCCGACCCTCAAACCACCGCGTACCG AACTTGAACGTTGCCTCGGCGGGGCCCTCCAGGGGGACCCGCCGCAGGACGCAAACAAGACCGTTTTGTGTG ACGAACGTCTGATATTCTTCTTCGCGAATGCGATAAAGTACAACTTTTAACAATGGATCTCTTGGCTCCGGCAT CGATGAAGAACGCAGCGAAATGCGATAACTAGTGTGAATTGCAGATTTCAGTGAATCATCGAGTCTTTGAAC GCACATTGCGCCCCCTGGTATTCCTAGGGGCATGCCTATTCGAGCGTCGTTTACTCCCTCAAGCGCAAGCTTGG TGTTGGGGATCGCCCCTCCCTTCGCGGGAGGCGGCGGCCCTTGAATCCATCGGCGGTGCCGGTGCGGCCTGG AGCGCAGCAGCGATGCAGCTCGTGGGGTCCGCTCGACGCCTGCCGTGCAAGCGTGGCTTCTCTCGCAGAACC

ACATTCCAAACGTCGACCTCGA

>77.1.2b(8) cynspe fungusB

AGGTGTGGCTCATACCTTACCCTTGCTTATACGCACCTTTTTGTTGTTTCCTCGGCGGGTCCGCTCGCCGATAG GAAGCCTATCAAACCCTTTGCATCAGCATGAAAACTTCTGAACAAACCCTAAATATTTACAACTTTCAACAATG GATCTCTTGGTTCWGGCATCGATGAAGAACGCAGCGAAATGCGATAAGTAGTGTGAATTGCAGAATTCAGTG AATCATCGAATCTTTGAACGCACATTGCGCCCCTTGGTATTCCATGGGGCATGCCTGTTCGAGCGTCATTTACA CCCTCAAGCTTTGCTTGGTGTTGGGCGTCTGTCCTGCTGTTTCAGCGTGGACTTGCCCTAAAGGTATTGGCAGC GGCTCAAGCCCGGCTTCTCGCGCAGCAGTTCGCGCTTCTTGAGGCATAAGGGGGGCCTGCGTCCATAAAGTCC ACTTCTTGACTTGACC

>217.1.1c(37) cynfle fungusC

CGGAAGGATCATTATCCATCTCAAACCAGGTGCGGTCGCGGCCCCCTTAACCGGGTGGTTCGCGCCGCATTCC TGCATCCTTTTTTTACGAGCACCTTTCGTTCTCCTTCGGCGGGGCAACCTGCCGCTGGAACTTAACAAAACCTTT TTTTGCATCTAGCATTACCTGTTCTGATACAAACAATCGTTACAACTTTCAACAATGGATCTCTTGGCTCTGGCA TCGATGAAGAACGCAGCGAAATGCGATAAGTAGTGTGAATTGCAGAATTCAGTGAATCATCGAATCTTTGAA CGCACATTGCGCCCCTTGGTATTCCATGGGGCATGCCTGTTCGAGCGTCATCTACACCCTCAAGCTCTGCTTGG TGTTGGGCGTCTGTCCCGCCTTTGCGCGCGGACTCGCCCCAAATTCATTGGCAGCGGTCTTTGCCTCCTCTCGC GCAGCACATTTGCGTCTGCGAGGGGGCGTGGCCCGCGTCCACGAAGCAACATTACCGTCTTTGACCTCGGATC AGGTAGGGA

>222.1.3d(14) cynhyb fungus spegazzinia

CCTGCGGAAGGATCATTACCTGAAGAGTCCAACGGGGGAGGCCAAACACCTCCCCCCGCCCGATTCTCCACCC TTCCTTCACACGTACCTCCTGTTCTTCCTCGGCGGGGCAACCCGCCGGCGGAACATCCACAAACCCTTTCGCAT CTAAGCAGTACTGTTCTGATAAACCAAAATCGTTACAATCTTCAACAATGGATCTCTTGGCTCTGGCATCGATG

AAGAACGCAGCGAAATGCGATAAGTAGTGTGAATTGCAGAATCCAGTGAATCATCGAATCTTTGAACGCACA TTGCGCCCCTCGGTATTCCGTGGGGCATGCCTGTTCGAGCGTCATTAACAACTCAAGCTCTGCTTGGTGTTGG GCGTCTGTCCGCGCCTCCGCGCGTGGACTCGCCCCAAAGACATTGGCAGCCTAGGCTGATGACCCGCCCGCA GCACATTGCGTCGGCGAGTGCGCAGCGGCCTTCGCGTCCACGAAGCTCTTCTTCCACAGATTTGACCTCG

>222.7.wp(2) cynhyb fungus (spegazzinia?)

TTCTCCACCCTTCCTTCACACGTACCTCCTGTTCTTCCTCGGCGGGGCAACCCGCCGGCGGAACATCCACAAAC CCTTTTGCATCTAAGCAGTACTGGTTCTGATAAACCAAAATCGTTACAATCTTCAACAATGGATCTCTTGGCTCT GGCATCGATGAAGAACGCAGCGAAATGCGATAAGTAGTGTGAATTGCAGAATCCAGTGAATCATCGAATCTT TGAACGCACATTGCGCCCCTCGGTATTCCGTGGGGCATGCCTGTTCGAGCGTCATTAACAACTCAAGCTCTGCT TGGTGTTGGGCGTCTGTCCGCGCCCCCGCGCGTGGACTCGCCCCAAAGACATTGGCAGCCTAGGCTGATGAC CCGCCCGCAGCACATTGCGTCGGCGAGTGCGCAGCGGCCTTCGCGTCCACGAAGCTCTTCTTCCACAGATTTG ACCTCGGATCAGG

>221.1.1b(31) cynuni fungus (spegazzinia?)

AACCTGCGGGAAGGATCATTACCTGAAGAGTCCAACGGGGGGAGGCCAAACACCTCCCCCCGCCCGATTCTC CACCCTTCCTTCACACGTACCTCCTGTTCTTCCTCGGCGGGGCAACCCGCCGGCGGAACATCCACAAACCCTTT TGCATCTAAGCAGTACCGTTCTGATAAACCAAAATCGTTACAATCTTCAACAATGGATCTCTTGGCTCTGGCAT CGATGAAGAACGCAGCGAAATGCGATAAGTAGTGTGAATTGCAGAATCCAGTGAATCATCGAATCTTTGAAC GCACATTGCGCCCCTCGGTATTCCGTGGGGCATGCCTGTTCGAGCGTCATTAAAACCTCAAGCTCTGCTTGGT GTTGGGCGTCTGTCCGCGCCTCCGCGCGTGGACTCGCCCCAAAGACATTGGCAGCCTAGGCTGATGACCCGC CCGCAGCACATTGCGTCGGCGAGTGCGCAGCGGCCTTCGCGTCCACGAAGCTCTTCTTCCACAGATTTGACCT CGGATCAGGTAG

>57.2.2e(8) cyngra fungusD

CCAACCCTATGTTTATTGAACCTCTGTTGCTTCGGCGGGCCCGTCTCACGACCGCCGGAGGAGTCGCCGCGAG GCGCCCCTCTGGCCCGCGCCCGTCGATGGCCAACCCCCTTCAAACTCTGTGTCAACCGTGTCATGATGTCTAAG TCTATGATTTAATTAAAGCAAAAACTTTCAACAACGGATCTCTTGGTTCTGGCATCGATGAAGAACGCAGCGA AATGCGATAAGTAATGCGAATTGCAGAATTCCAGTGAGTCATCGAATCTTTGAACGCACATTGCGCCCTTTGG TATTCCGAAGGGCATGCCTGTTCGAGCGTCATTATCACCCCCTCAAGCCCCGTGCTTGGTGTTGGACGGCCGG TCGAGCGATCGACCCCTCCGAAAGACAATGACGGCGGGCTGTGGTTCCCCCGGTACACTGAGCTTCTCACCGA GCACGTACCGGAGCGAGGGCACCCGGCACCCGGTCCC

>222.1.3d(52) cynhyb fungusE

TGCGGAGGGATCATTACTGAGTTCTGGGTCTCCAAGAACCCGACCTCCAACCCTTTGTTGTCCGACTCTGTTGC CTCGGGGGTGACCCTGACCCGCCCGCGGGTTTTTGGGGCCCCCGGTGGACATATATAAACTCTGCTTCAATTT GTCGTCTGAGTAAATTGATCAAATCAAATCAAAACTTTCAACAACGGATCTCTTGGTTCTGGCATCGATGAAGA ACGCAGCGAAATGCGATAAGTAATGTGAATTGCAGAATTCAGTGAATCATCGAATCTTTGAACGCACATTGCG CCCTTTGGTATTCCGAAGGGCATGCCTGTTCGAGCGTCATTACACCACTCAAGCCTCGCTTGGTATTGGAGGA CGCGGTTCGCCGCGCCTCTTGAATCTTTCGGCTGAGCATATCGTCTCTTAGCGTTGTGATACATATTCGCTTGT GAGGTCGGTAGTGTTTGCGCCGTTAAACCCCCAAATTTTAAAGGTTGACCTCG

>222.1.3d(11)red cynhyb fungusE

GGTGAACCTGCGGAGGGATCATTACTGAGTTCTGGGTCTCCAAGACCCGACCTCCAACCCTTTGTTGTCCGAC TCTGTTGCCTCGGGGGTGACCCTGACCCGCCCGCGGGTTTTTTGGGGCCCCCGGTGGACATATATAAACTCTG CTTCAATTTGTCGTCTGAGTAAATTGATTAAATCAAATCAAAACTTTCAACAACGGATCTCTTGGTTCTGGCATC GATGAAGAACGCAGCGAAATGCGATAAGTAATGTGAATTGCAGAATTCAGTGAATCATCGAATCTTTGAACG

CACATTGCGCCCTTTGGTATTCCGAAGGGCATGCCTGTTCGAGCGTCATTACACCACTCAAGCCTCGCTTGGTA TTGGAGGACGCGGTTCGCCGCGCCTCTTGAATCTTTCGGCTGAGCATATCGTCTCTTAGCGTTGTGATACATAT TCGCTTGTGAGGTCGGTAGTGTTTGCGCCGTTAAACCCCCAAAT

>222.1.3d(50) cynhyb fungusE

GAGGGATCATTACTGAGTTCTGGGTCTCCAAGACCCGACCTCCAACCCTTTGTTGTCCGACTCTGTTGCCTCGG GGGTGACCCTGACCCGCCCGCGGGTTTTTTGGGGCCCCCGGTGGACATATATAAACTCTGCTTCAATTTGTCG TCTGAGTAAATTGATTAAATCAAATCAAAACTTTCAACAACGGATCTCTTGGTTCTGGCATCGATGAAGAACGC AGCGAAATGCGATAAGTAATGTGAATTGCAGAATTCAGTGAATCATCGAATCTTTGAACGCACATTGCGCCCT TTGGTATTCCGAAGGGCATGCCTGTTCGAGCGTCATTACACCACTCAAGCCTCGCTTGGTATTGGAGGACGCG GTTCGCCGCGCCTCTTGAATCTTTCGGCTGAGCATATCGTCTCTTAGCGTTGTGATACATATTCGCTTGTGAGG TCGGTAGTGTTTGCGCCGTTAAACCCCCAAATTTTAAAGGTTGACCTCGGATCAGGTA

>222.1.3d(51) cynhyb fungusE

AGGGATCATTACTGAGTTCTGGGTCTCCAAGACCCGACCTCCAACCCTTTGTTGTCCGACTCTGTTGCCTCGGG GGTGACCCTGACCCGCTCGCGGGTTTTTGGGGCCCCCGGTGGACATATATAAAACTCTGCTTCAATTTGTCGTC TGAGTAAATTGATTAAATCAAATCAAAACTTTCAACAACGGATCTCTTGGTTCTGGCATCGATGAAGAACGCA GCGAAATGCGATAAGTAATGTGAATTGCAGAATTCAGTGAATCATCGAATCTTTGAACGCACATTGCGCCCTT TGGTATTCCGAAGGGCATGCCTGTTCGAGCGTCATTACACCACTCAAGCCTCGCTTGGTATTGGAGGACGCGG TTCGCCGCGCCTCTTGAATCTTTCGGCTGAGCATATCGTCTCTTAGCGTTGTGATACATATTCGCTTGTGAGGTC GGTAGTGTTTGCGCCGTTAAACCCCCAAATTTTAAAGGTTGACC

>220.1.4b(5) cynspe fungusF

AACCCATGTGAACTTACTGTTTGTTGCCTCGGCGGGTAGGCGTGCGTAGCGAAGCTACCCTGCAGTGGCGGA GAGCCTTCCCTGTAACCGGGGGAGGCTACCCGGCACTGCTCGGGCCGGCCCACCCTACCGCGCCGCTCCAGC CCGCCGACGGCCCAACCCAACTCTGTTTGTATAGTGGCACTCTGAGTGATTAAACAAATAATCAAAACTTTCAA CAACGGATCTCTTGGTTCTGGCATCGATGAAGAACGCAGCGAAATGCGATAAGTAATGTGAATTGCAGAATTC AGTGAATCATCGAATCTTTGAACGCACATTGCGCCCATTAGTATTCTAGTGGGCATGCCTGTTCGAGCGTCATT ACGACCATTAAGCCTAGTTGCTTAGCATTGGGAATCCTACGTCCGTAGTTCCTCAAAGACATTGGCGGAGCCG AGTCAGTCTCTAAGCGTAGTAATTTTTTTCTCGCTTCTGTAGCTGGCTTGTCCCTCGCCATAAAACCCCCAACTT TATAGGT

>56.1.1b(1) cynrid Diversimediispora

AGGGATCATTACAGAGTTATCTAACTCCCAAACCCATGTGAACTTACCTTTGTTGCCTCGGCAGAAGCTACCCT GTAGCGACTGCCGGTGGACTACTAAACTCTTGTTATTTTTAAGTAATCTGAGCGTCTTATTTTAATAAGTCAAA ACTTTCAACAACGGATCTCTTGGTTCTGGCATCGATGAAGAACGCAGCGAAATGCGATAAGTAATGTGAATTG CAGAATTCAGTGAATCATCGAATCTTTGAACGCACATTGCGCCCATTAGTATTCTAGTGGGCATGCCTGTTCGA GCGTCATTTCAACCCTTAAGCCTAGCTTAGTATTGGGAATTGGCTTGTCATGAGCCATTCTTCAAATTCAACGG CGGATTTATAGCAATCTCTGAACGTAGTAATCTTATTCTCGTTTTTGCGATACTATAAACCTCAGCCGCTAAACC CCCAATTTTTT

>77.1.1b(1) cynspe fungusH

AGGGATCATTACAGAGTTGCAAAACTCCAAACCCATGTGAACATACCTCGTGTTGCCTCGGCGGCGAGCGTAG TGCTCGGGTGGTGCCTACCCGGTACTTACCCTTGAGCTACCCTGGAGCTCCCTACCCGGGAGCTGCACGGTAC CTACCCTGGAGGACTTGCGTATCCGCCGAAGGACCACTTAAACTCTTGTTTCTGTGTGGCACATCTGAGTTATT ATATTAATAAGTCAAAACTTTCAACAACGGATCTCTTGGTTCTGGCATCGATGAAGAACGCAGCGAAATGCGA TAAGTAATGTGAATTGCAGAATTCAGTGAATCATCGAATCTTTGAACGCACATTGCGCCCATTAGTATTCTAGT

GGGCATGCCTGTTCGAGCGTCATTAAGTCCATTAAGCCTAGTTGCTTAGCGTTGGGAGCATTACCGCACGGTA ACTCCTTAAAGTCAGTGGCAGGGTTACGGTACACTCTAAGCGTAGTAGATTTCTTTCTCGCTTCTGTTGTGGCC GCGGCCGTTGCCATAAACCTATAATTTTTTATTGGT

>222.7.wp(3) cynhyb fungusI

ACCAGCGGAGGGATCATTATAGAGTTTTCTAAACTCCCAACCCATGTGAACTTACCTTTTGTTGCCTCGGCAGA AGTTATAGGACTTCTTATAGCTGCTGCCGGTGGACCACTAAACTCTTGTTATTTTATGTAATCTGAGCGTCTTAT TTTAATAAGTCAAAACTTTCAACAACGGATCTCTTGGTTCTGGCATCGATGAAGAACGCAGCGAAATGCGATA AGTAATGTGAATTGCAGAATTCAGTGAATCATCGAATCTTTGAACGCACATTGCGCCCATTAGTATTCTAGTGG GCATGCCTGTTCGAGCGTCATTTCAACCCTTAAGCCTAGCTTAGTGTTGGGAATCTACTTCTCCTAGGAGTCGT AGTTCCTGAAATACAACGGCGGATTTATAGCATCCTCTGAGCGTAGTAAATTTTTTCTCGCTTCTGTCAGGTGC TGTAACTCCCAGCCGCTAAACCCCCCAATTTTTTGTGGTTGACCTCGGATCAGG

>221.1.1b(40) cynuni fungusZ

TGCGGAGGGATCATTACTGAGTTCTGGGTCTCCAAGACCCGACCTCCAACCCTTTTGTTGTCCGACTCTGTTGC CTCGGGGGTGACCCTGACCCGCCCGCGGGTTTTTGGGGCCCCCGGTGGACATATATAAACTCTGCTTCAATTT GTCGTCTGAGTAAATTGATTAAATCAAATCAAAACTTTCAACAACGGATCTCTTGGTTCTGGCATCGATGAAGA ACGCAGCGAAATGCGATAAGTAATGTGAATTGCAGAATTCAGTGAATCATCGAATCTTTGAACGCACATTGCG CCCTTTGGTATTCCGAAGGGCATGCCTGTTCGAGCGTCATTACACCACTCAAGCCTCGCTTGGTATTGGAGGA CGCGGTTCGCCGCGCCTCTTGAATCTTTCGGCTGAGCATATCGTCTCTTAGCGTTGTGATACATATTCGCTTGT GAGGTCGGTAGTGTTTGCGCCGTTAAACCCCCAAATTTTAAAGGTTGACCTCGGAT
