# Supplementary material for: Fungal Diversity of Selected Habitat Specific Cynorkis Species (Orchidaceae) in the Central Highlands of Madagascar
Source: Microorganisms. 2021 Apr 10;9(4):792. doi: 10.3390/microorganisms9040792 (PMC8069969; doi:10.3390/microorganisms9040792)
Supplement: Supplementary file 1 [file microorganisms-09-00792-s001.zip › Mycorrhizal fungi list.docx]

***Rhizoctonia* OTUs**

>OTUser3 *C. graminea*

GTCTGTGCTGGTGGGCAACACATGTGCACGTCAGCCGCAAATCCACACACCCTGTGCCACCTTTGACTTTGGGATCCACTGGTCTTTTGACTTGCTAGCAGGTTGGCTTGTAGCCATCCTGGATCTCAGAGTACTTTTACATACTCTTGAATGTAATGGAACGTCTCTGTGCTTAATGCGCAAATATACAACTTTCAACAACGGATCTCTTGGCTCTCGCATCGATGAAGAACGCAGCGAAATGCGATAAGTAATGTGAATTGCAGAATTCAGTGAATCATCGAATCTTTGAACGCACCTTGCACCCTTTGGTATTCCGAGGGGTACACCCGTTTGAGTGTCATTGTAATCTCACTCCGACGACTTTTTTGTTGTCCGGAGGTGGACTTGGACCTTGCCGTGTCATTATGGCTGGTCTGAAATGCATGAGTGCACCCTGCTGTAGCAGCGTCTTCACTGTGATAAGTATCTTCACTGATTCAAGTTCCCTTGGGACACGCTCGCCTGGAGGGCTCTGTGCTTCCAACCGTCTGCATAGACAAACC

>OTUser1 *C. purpurea*

TCTGTGCTGGTGGCAACACATGTGCACGTTGATCGCAAATCCACACACCTGTGCACTTTTGACTTTGGGATCCGCTTGTTGGCTTGTCYGACTTGCAGCTGGTTGGCCTTGTGCCGGCTTGGRTCTCAGAGTAATTTTTACACACTCTTAYTGTAATGGAATGTCTTGTGCATAATGCATATAAATACAACTTTCAACAACGGATCTCTTGGCTCTCGCACCGATGAAGAACGCAGCGAAATGCGATAAGTAATGTGAATTGCAGAATTCAGTGAATCATCGAATCTTTGAACGCACCTTGCACCTTTTGGCATTCCGAAAGGTACACCCGTTTGAGTGTCATTGTAATCTCACTCCTTCAACTTTTGTTGATGGATGTGGACTTGGACCGTGTTGTGTTACTACAACTGGTMTTAAATGTATGAGTACACCCTGCTGTTGCAGCGTCTTCAGTGTGATAAGCATCTTCACTGGATAAAGTTCCCTCGGGACACGCRGGCATTGTGGGCTCTGTGCTACCAACYGTCTTTGGACAATCTTTGACAATTT

>OTUcer2 *C. speciosa*

TGCGGAAGGATCATTATCGAAATCGGAGTTTGGTTGTAGCTGGCCTGCTAAAGGCACGTGCACGCCTTTCTCTTTTCATCCACACACCCCTGTGCACCTGTGAGACGGAGGCCTGTCGTCCCCTTTCAGTAGGGGATCGGGTTTCCGCCTGTCGTCATACAAAATCAATTTGTTTCAACTCGAATGTAATTGACGTGAATGCGTCTTATATACTAAGTTTCAACAACGGATCTCTTGGCTCTCGCATCGATGAAGAACGCAGCGAAATGCGATAAGTAATGTGAATTGCAGAATTCAGTGAATCATCGAATCTTTGAACGCACCTTGCGCTCCTTGGTATTCCTTGGAGCATGCCTGTTTGAGTATCATGAAATTCTCAAAGTAAATCTTTCGTTAACTCGACAGACTTTGCTTTGGACTTGGAGGTCTGCAGACTTCGCGGTCTGCTCCTCTCAAATGCATCAGCTGGATCTCGGTGAGCTTGGTTCCACTCGGCGTGATAAGTATCACTCGCTGAGGACACCCGTACAACAGGTGGCCAGGGTCGCGGATGAACCGCTTCTAACCGGTCTATTGACTTAGACAATACATTTATGATCTGATCTCAA

>OTUcer2 *Cynorkis* sp.

TGAACCTGCGGAAGGATCATTATCGAAATCGGAGTTCGGTTGTAGCTGGCCTGCTAAAGGCACGTGCACGCCTTTCTCTTTTCATCCACACACCCCTGTGCACCTGTGAGACGGAGGCCTGTCGTCCCCTTTCAGTAGGGGATCGGGTTTCCGCCTGTCGTCATACAAAATCAATTTGTTTCAACTCGAATGTAATTGACGTGAATGCGTCTTATATACTAAGTTTCAACAACGGATCTCTTGGCTCTCGCATCGATGAAGAACGCAGCGAAATGCGATAAGTAATGTGAATTGCAGAATTCAGTGAATCATCGAATCTTTGAACGCACCTTGCGCTCCTCGGTATTCCTTGGAGCATGCCTGTTTGAGTATCATGAAATTCTCAAAGTAAATCTTTCGTTGACTCGACAGACTTTGCTTTGGACTTGGAGGTCTGCAGACTTCGCGGTCTGCTCCTCTCAAATGCATCAGCTGGATCTCGGTGAGCTTGGTTCCACTCGGCGTGATAAGTATCACTCGCTGAGGACACCCGTACAACAGGTGGCCAGGGTCGCGGATGAACCGCTTCTAACGGTCTATTGACTTAGACAATACATTTATGATCTGATCTCAAATC

>OTUser3 *C*. x *ranaivosonii*

GTCTGTGCTGGTGGCAACACATGTGCACGTCAGCCGCAAATCCACACACCTGTGCACCTTTGACTTTGGGATCCACTGGTCTTTTGACTTGTAGCAGGTTGGCTTGTGCCATCCTGGATCTCAGAGTACTTTTACATACTCTTGAATGTAATGGAACGTCTCTGTGCTTAATGCGCAAATATACAACTTTCAACAACGGATCTCTTGGCTCTCGCATCGATGAAGAACGCAGCGAAATGCGATAAGTAATGTGAATTGCAGAATTCAGTGAATCATCGAATCTTTGAACGCACCTTGCACCCTTTGGTATTCCGAGGGGTACACCCGTTTGAGTGTCATTGTAATCTCACTCCGACGACTTTTTTGTTGTCYGGAGGTGGACTTGGACCTTGCCGTGTCATTATGGCTGGTCTGAAATGCATGAGTGCACCCTGCTGTAGCAGCGTCTTCAGTGTGATAAGTATCTTCACTGATTCAAGTTCCCTTGGGACACGCTYGCCTGGTGGGCTCTGTGCTTCCAACCATCTGCATAGACAAACCCTTGACAATTTGACC

>OTUser4 *C*. x *ranaivosonii*

GTAGGTGAACCTGCGGAAGGATCATTAACGAATGACAAGTCGGCCTTCTGTGCTGGTGGGTCTCCCACATGTGCACGTCGGTCGCAAATCCACACACCTGTGCACCTTTGACTTTGGGATCCGCTTGTTTATCGAGCAGCTGGTCATTTGCTTGGCCTGGGTCTCAGAGTAATTTTTACACACGCTGAATGTAATGGAATGTCTTTTGTGCTTAACGCGCATTATATACAACTTTCAACAACGGATCTCTTGGCTCTCGCACCGATGAAGAACGCAGCGAAATGCGATAAGTAATGTGAATTGCAGAATTCAGTGAATCATCGAATCTTTGAACGCACCTTGCACCCTTTGGTATTCCGAAGGGTACACCCGTTTGAGCGTCATTGTAATCTCACTTCAACGACTTTATTGTTGCTGGAGGTGGACCTGGACCCTTGCCGTGTCACAAYGGCTGGTCTGAAATGCATGAGTACACCCTGCTGTCGCAGCGTCTCAGTGTGATAAGCATCTTCACTGAACAAGCTCCCTCTCGAGGGACACGTCTGCGTTGTGGGCTCTGGGCTTCCAACCGTCTTGTGCAGAGACAATCTTTGACAATTTGACCTCAAATCGGGTGGGACT

>OTUcer4 *C. purpurea*

TCGGTTGTAGCTGGCCTTTCATTCGGGCATGGTGCACGCCTTCTCTTTCATCCACAACACACCTGTGCACCTGTGAGACGGGGAGGCATTTCTCGCGAGAGAGAGAGAGAGAGAGAGGTCTCCGTCTGCTAATACATAAACTCATATATTGTAATCTGAATGTAATTTGATGTAACGCATCTATGAACTAAGTTTCAACAACGGATCTCTTGGCTCTCGCATCGATGAAGAACGCAGCGAAATGCGATAAGTAATGTGAATTGCAGAATTCAGTGAATCATCGAATCTTTGAACGCACCTTGCGCTCCTTGGTATTCCTTGGAGCATGCCTGTTTGAGTATCATGAAATTATCAAAACAAGTCTTTTGTTCGTTCGAATGGCTTTTCGTTTTGGAGTTGGAGGCCTTGCAGATCCACGTCTGCTCCTCTTAAATGCATCAGCTGGATCTCTGCGAGCTCGGTTCCACTCGGCGTGATAAGTATCGCTCGCTGAGGACGCCTGCAACAAGGTGGCCGGGATCNCAGATGAACCGCTTCCAATCGTCCGTTAACTCGGACAACTACTTTATGATCTGATCTCAA

>OTUtul4 *C. purpurea*

ATCGTCCTTGGGACGTTAAGGTGCTCTGGTCGAGGATAAACGACCCCTCTGACAGAGGTGAATCCGTCCTAGTGTTACCTCGTCACCGAGGCACACGTTAAAGATTGTTCCATGTTGTGAGTCTAACACCAGTTGTAAAAACAAATACAACCGGTAGCGCTGGATCCCTGGGCACGTCATTCGATGAAGACCGTTGCAAATTGCGATAAAGTGATGTGATGCGCAAGTCCACCACTTATACGTGAATCATCGAGTTGTTGAACGCACTGCACCGCGCCCTAACCCGGCTGCGGTATGCCCCTTTGAGCGTCATTGTATCCCTTCGGGAGTCTTTCTTAGACCCGAGTTTGGAGTCCTTGGTCTTTGGATCATGTTCTCTTAGATGCGTCGCGCTTATCGCCTGATGGGTCACTCTAATGCCTGAGCGTGGAGTCCCTCAGAGCTGAGATGCGCTTGACCGAGTGTTGAGCTTGTGTCGCCAAGTCCACGTTCTTTTGGGATGTTGGTACTACAATGCATGACCTCATTGGGGTAGGACAA

>OTUtul3 *C. purpurea*

ATCGTCCTCGGGACGTTAAGGTGCTCTGGTCGAGGATAAAGGACCCCTCTGACAGAGGTGAATCCGTCCTGGTGTTACCTCTTCACCGAGGCACACGTTAAAGATCGTTCSGCGTTGTGAGTCTAACACCAGTTGTAAAAACAATTACAACCGGTAGCGCTGGATCCCTTGGCACGTCATTCGATGAAGACCGTTGCAAATTGCGATAAAGTGATGTGATGCGCAAGTCCACCACTTATACGTGAATCATCGAGTTGTTGAACGCACTGCACCGCGCCCTAACCCGGCTGCGGTATGCCCCTTTGAGCGTCATTGTATCCCTTCGGGAGTCTTTCTTAGACCCGAGTTCGGAGTCCTCGGTCTGCGGACCGTGTTCTCTTAGATGCGTCGTGCTGATCGTCCGATGGGTCACTCTAATGCCTGAGCGTGGAGTCCCTCGGAGTCGAGATGCGCTTGACCGAGTGTTGAGCTCGCGTCGCCAAGTCCGCGTTCCTCTGGGACGTCGGTACTACAACGCATGACCTCATTGGGGTAGGACAA

>OTUtul5 *C. speciosa*

TTTGACGTTCGCTTTTTCCGTTGTCCTCGGGACGTTAACGTGCTCTGGTTGAGGATAAATGACCCCTCTGACCGAGGTAAAACCTGTCGCTCTGTGTTACCTTCCGAGGCACACGTTAAAGATTGTTCCGTGTTGTGAGTCTAACACCAGTTGTATAAACTTTTTACAACCGGTAGCGATGGATCCCTTGGCACGTCATTCGATGAAGACCGTTGCAAATTGCGATAAAGTGATGTGATGCGCAAGTCCACCACTTATACGTGAATCATCGAGTTGTTGAACGCATTGCACCGCGCCCTAAACCGGCTGCGGTATGCCCCTTTGAGCGTCATTGTATTCCTTCGGGAGTCCGTTTACAAGGACCCGAGTTCGGAGTCCTCGGTCCTCTTCTGGATCGTGTTCTCTTAGATGCGTCGCACCGATYGCCTGATGGGTCCTCTAATGCCTAAGCGTGGAGTTCCTTCAGAGTCCGAGACGTGCTTGACCGGGTGTTGAGCTCGCGTCGCCAAGTCTGCCTTAACCAGCAGTACTACAACGCATGACCTCATTGGGGTAGGACAACCCGCTAGACTTAAGCATATTAATCAGCGGAGGAAAAGAAACTAACCAGGATTCCCTCAGTAA

>OTUtul5 *C. fastigiata*

AATCGTCTTTGACGTTCGCTTTTCCGTTGTCCTCGGGACGTTAATGCGCTCTGGTCGAGGATAAACGACCCCTCTGACCGAGGTCAAACCTGTCACTGTGTTACCTCTTTGCTGAGGCACACGTTAAAGATCGTTCCGCGTTGTGAGTCTAACACCAGTTGTATAAACTTTTTACAACCGGTAGCGATGGATCCCTTGGCACGTCATTCGATGAAGACCGTTGCAAATTGCGATAAAGTGATGTGATGCGCAAGTCCACCACTTATACGTGAATCATCGAGTTGTTGAACGCATTGCACCGCGCCCTAATCCGGCTGCGGTATGCCCCTTTGAGCGTCATTGTAATCCTTCGGGAGTCCTTTTAACTAAGGACCCGAGTTCGGAGTCCTCGGTCCTCTGGATCGTGTTCTCTTAGATGCGTCGCACCGATCGCCTGATGGGTCCTCTAATGCCTAAGCGTGGAGTTCCTTCAGAGTCCGAAACGTGCTTGACCGGGTGTTGAGCTCGCGTCACCAAGTCTGCCTAACCAGCAGTACTACAACGCATGACCTCATTGGGGTAGGACAACCCGCTAGACTTAAGCATATTAATCAGCGGAGGAAAAGAAACTAACTAGGATTCCCTAGTAACTGCG

>OTUtul6 *C. x ranaivosonii*

TTGACGTACGCTAAGTCTCCGTCGTCCTCGGGACGTTAAGGCGCTCTGGTCGAGGATAAATGACCCCCTCTGACCGAGGCTAATCCGTCGCGCCTTTCCGTGTTACCGTCCGCGGCACACGTTAAAGATCGTTCCGCGTTGTGAGTCTTTCTGGTTGTAAAAAACACTTTACAACCGGTAGCGATGGATCCCTTGGCACGTCATTCGATGAAGACCGTTGCAAATTGCGATAAAGTGATGTGATGCGCAAGTCCACCACTTATACGTGAATCATCGAGTTGTTGAACGCATTGCACCGCGCCCTAAACCGGCTGCGGTATGCCCCTTTGAGCGTCATTACATCCTTCGGGAGTCTCCTTTGCTGGAGACCCGAGTTCGGAGTCCTCGGTCCTTTGGGATCGTGTTCTCTCAGATGCATCGCGCCGATCGCTTTGATGGGTAACTCTAATGCCTGAGCGTGGAGTCCCTCTGGAGCCGAGACGCGCTTGACCGGGTGGTGAGCCCGTGTCGCCAAGTCCGTTGTCGTTCGCGACGTCGGTACTACAACCTCATGACCTCATTGGGGTAGGACAA

>OTUtul8 *C. speciosa*

GACGTACTGTTTCCGTCGTCCTCGGGACGTTAAGGYGCTCTGGTCGAGGATAAACGACCCCTCTGACCGAGGTAAAGCGGTCCTTGTGTTACCTCTCGAGGCACACGTTAAAGATCGTTCCGCGTTGTGAGTCTAAACCAGTTGTAACTTTTTACAACCGGTAGCGCTGGATCCCTTGGCACGTCATTCGATGAAGACCGTTGCAAATTGCGATAAAGTGATGTGATGCGCAAGTCCACCACTTATACGTGAATCATCGAGTTGTTGAACGCATTGCACCGCGCCCTAATCCGGCTGCGGTATGCCCCTTTGAGCGTCATTGTATCCCTTCGGGAGCCTTTTCGTTAAGGCCCGAGTTCGGAGTCCTCGGTCTTTGGATCGTGTTCTCTTAGATGCGTCGCGCCGATCGCCTGATGGGTCGCTCCAATGCCTAAGCGTGGAGTCCCTCGGAGCYGAGACGCGCTTGACCGGGTGTTGAGCTCGCGTCGCCAAGTCCGCACGTCTCTGACGTCGGTACTACAACGCATGACCTCATTGGGGTAGGACAACCCGCTAGACTTAAGCATATTAATCAGCGGAGGAAAAGAAACTAACCAGGATTCCCTCAGtAActgc

>OTUtul9 *C. flexuosa*

TTCCGTCGTCCTCGGGACGTTAAGGTGCTCTGGTCGAGGATAAACAACCCCTCTGACCGAGGTTAAACGGTCGCTTGCCTGTGTTACCTCGTCCGAGGCACACGTTAAAGATCGTTCCGCATTGTGAGTCTAACACCAGTTGTAACTTTTTACAACCGGCAGCGCTGGATCCCTTGGCACGTCATTCGATGAAGACCGTTGCAAATTGCGATAAAGTGATGTGATGCGCAAGTCCACCACTTATACGTGAATCATCGAGTTGTTGAACGCACTGCACCGCGCCCTAATCCGGCTGCGGTATGCCCCTTTGAGCGTCATTGTATTCCTTCGGGAGTCTTTCCTTGCGGAAGACCCGAGTTCGGAGTCCTCGGTCTTTGGATCGTGTTCTCTCAGATGCGTCGCGCCGATCGCCTGATGGGTACTCTAATGCCTGAGCGTGGAGTCCCTCGGAGCTTGAGACGCGCTTGACCGGCCGTTGGGCTCGCGTCGCCAAGTCCGCGTCCTTTGGGACGGCGGTACTACAATGCATGACCTCATTGGGGTAGGACAACCCGCTAGACTTAAGCATATTAATCAGCGGAGGAAAAGAAACTA

>OTUtul8 *C. flexuosa*

CTTTGACGTGCTGTTTCCGTCGTCCTCGGGACGTTAAGGCGCTCTGGTCGAGGATAAACGACCCCTCTGACAGAGGTAAAGCCGTCCTGTGTTACCTCTTTGCCGAGGCACACGTTAAAGATCGTTCCGCGTTGTGAGTCTAACACCAGTTGTAACACTTTTACAACCGGTAGCGCTGGATCCCTTGGCACGTCATTCGATGAAGACCGTTGCAAATTGCGATAAAGTGATGTGATGCGCAAGTCCACCACTTATACGTGAATCATCGAGTTGTTGAACGCACTGCACCGCGCCCTAATCCGGCTGCGGTATGCCCCTTTGAGCGTCATTGTATCCCTTCGGGAGTCTTTTCGTTAAGACCCGAGTTCGGAGTCCTCGGTCTTTGGATCGTGTTCTCTTAGATGCGTCGCGCCGATCGCCTGATGGGTCACTCTAATGCCTGAGCGTGGAGTCCCTCGGAGCTGAGACGCGCTTGACCGAGTGTTGAGCTCGCGTCGCCAAGTCCGCACGTCTTGGACGTCGGTACTACAACGCATGACCTCATTGGGGTAGGACAACCCGCTAGACTTAAGCATATTAATCAGCGGAGGAAAAGAAACTAACCAGGATTCCCTCAGTAACTG

>OTUtul10 *C. gibbosa*

TTACCTCCTCAGAGGCACACGTTAAAGATCGTTCCGCGTTTSTGAGTCTCGACACCGGTTGGAAAACACGTTTACAACCGGTAGCGCTGGATCCCTTGGCACGTCATTCGATGAAGACCGTTGCAAATTGCGATAAAGTGATGTGATGCGCAAGTCCACCACTTATACGTGAATCATSGAGTTGTTGAACGCACTGCACCGCGCCCTAAACCGGCTGCGGTATGCCCCTTTGAGCGTCATTGTTACCCTTCGGGAGTCTTTTTCTTCAAAGACCCGAGTTCGGAGTCCTCGGTCCTTGTTGGATCGTGTTCTCTCAGATACATCGCGCCGATCGTCCGGTGGGTCCTCTAATGCCTGAGCGTGGAGGGCCTCTGGGGTTTCGAGAGGCGTCCGACCGCGTGTTGAAGCGCCGGTCAGCAAGTCCCGCACGGCTCAGCCTGTCGGTGCTACAGCTTCATGACCTCATCGGGGTAGGACAACCCGCTAGACTTAAGCATATTAATCAGCGGAGGAAAAGAAACTAACCA

>OTUtul9 *C. gibbosa*

aTTCCGTCGTCCTCGGGACGTTAAGGCGCTCCGGTCGAGGATAAACGACCCCTCTGACCGAGGTTAAACGGTCGCTGCCTGTGTTACCTCTTCGGAGGCACACGTTAAAGATCGTTCCGCGTTGTGAGTCTAACACCAGTTGTAACTTTTTTACAACCGGCAGCGCTGGATCCCTTGGCACGTCATTCGATGAAGACCGTTGCAAATTGCGATAAAGTGATGTGATGCGCAAGTCCACCACTTATACGTGAATCATCGAGTTGTTGAACGCATTGCACCGCGCCCTAATCCGGCTGCGGTATGCCCCTTTGAGCGTCATTGTATTCCTTCGGGAGTCTTTCCTTGCGAAAGACCCGAGTTCGGAGTCCTCGGTCTTCGGATCGTGTTCTCTCAGATGCGTCGCGCCGATCGCCTGATGGGTACTCTAATGCCTGAGCGTGGAGTCCCTCGGGGTTTGAGACGCGCTTGACCGGCCGTTGGGCTCGCGTCACCAAGTCCGCGTCCTTTGGGACGGCGGTACTACAACGCATGACCTCATCGGGGTAGGACAACCCGCTAGACTTAAGCATATTAATCAGCGGAGGAAAAGAAACTAACCAGGATTCCCCTAG

>OTUtul8 C. *x ranaivosonii*

aatcGTCTTTGACGTACTGTTTCGTCGTCCTCGGGACGTTAAGGYGCTCTGGTCGAGGATAAACGACCCCTCTGACCGAGGTAAAGCGGTCCTTGTGTTACCTCTCGAGGCACACGTTAAAGATCGTTCCGCGTTGTGAGTCTAAACCAGTTGTAACTTTTTACAACCGGTAGCGCTGGATCCCTTGGCACGTCATTCGATGAAGACCGTTGCAAATTGCGATAAAGTGATGTGATGCGCAAGTCCACCACTTATACGTGAATCATCGAGTTGTTGAACGCATTGCACCGCGCCCTAATCCGGCTGCGGTATGCCCCTTTGAGCGTCATTGTATCCCTTCGGGAGCCTTTTCGTTAAGGCCCGAGTTCGGAGTCCTCGGTCTTTGGATCGTGTTCTCTTAGATGCGTCGCGCCGATTGCCTGATGGGTCGCTCCAATGCCTAAGCGTGGAGTCCCTCGGAGCCGAGACGCGCTTGACCGGGTGTTGAGCTCGCGTCGCCAAGTCCGCACGTCTCTGACGTCGGTACTACAACGCATGACCTCATTGGGGTAGGACAACCCGCTAGACTTAAGCATATTAATCAGCGGAGGAAAAGAAACTAACCAGGATTCCCTCAGTAACTGCGA

>OTUtul11 *C. ridleyi*

GTCGTCCCCGGGACGTTAAGGTGCTCTGGTCGAGGATAAACRACCCCTCTGACCGAGGCTAATCCGTCGTCCTTTCCGTGTTACCGCTCCCGGCACACGTTAAAGAGCGTTCCGCGTTGTGAGTCTCACTCGGTTGTTAAACGATCGTTTACAACCGGTAGCGATGGATCCCTTGGCACGTCATTCGATGAAGACCGTTGCAAATTGCGATAAAGTGATGTGATGCGCAAGTCCACCACTTATACGTGAATCATCGAGTTGTTGAACGCACTGCACCGCGCCCTAAACCGGCTGCGGTATGCCCCTTTGAGCGTCATTGTCATCCTTCGGGAGTCTTTGCTTGCAAAGACCCGAGTTCGGAGTCCTCGGTCCCCACCGTGTTCTCTCAGATGCGTCGYGCCGATCGCCTTTGACGGGTTCTCTAATGCCTGAGCGTGGAGTCCCGCGAGCCGAGACGCGTCCGACCGGGCGTTGGCCCGTGTCAGCAAGTCCCAGACGCAAGTCTGCGGTACTACAACACATGACCTCATCGGGGTAGGACAACCCGCTAGACTTAAGCATATTAATCAGCGGAGGAAAAGAAACTAACCAGGATTCCCTCAG

**Non-Rhizoctonia OTUs**

>OTUcop1 *C. speciosa*

TAACGAATAACTATGGTGTCTTGGTTGTCGCTGGCTCCTCGGAGCATCGTGCACGCCCGCCATTTTTATCTATCCACCTGTGCACCGAATGTAGGTCTGGATGACTCTCGCCTCCGGGCGGATGCGAGGTTTGCGTTTCGCGTGCGAGCGCTCTCCTCGAATTTCCAGGCTCTACGTCTCTTTACACACCCCAAACGTATGATGCAGAATGTAGTCAATGGGCCTTTACAAGCCTATAAAACACTATACAACTTTCAGCAACGGATCTCTTGGCTCTCGCATCGATGAAGAACGCAGCGAAATGCGATAAGTAATGTGAATTGCAGAATTCAGTGAATCATCGAATCTTTGAACGCACCTTGCGCTCCTTGGTATTCCGAGGAGCATGCCTGTTTGAGTGTCATTAAATTCTCAACCTCACCGGTTTTCTGAACCGTTCTTCGAGGCTTGGATGTGGGGGCTTGTGCAGGTCGCCTCAGCGCGGTCTGCTCCCCTGAAATGCATTAGCGAGATTCATTCTGGAACCTCCGTCTATCGGTGTGATAATTATCTACGTCGTTGACTTGGTTCGGACTCGCTTCTAACCGTCCGCGAGGACAACATACTTGACAATTG

>OTUpar1 *C*. x *ranaivosonii*

AGGATCATTAATCCATCTAAACAGCGTGGCTGCGGCCTCCGGGGTCAAACCTGGGCGGTTCGCGCTACCTGTCTACATCCTTTTTTTACGAGCACCTTTCGTTCTCCTTCGGCGGCTTACCCCGCCGTTGGAATCAAACAAAACCTTTTTTGCATCTAGCATTACCTGTTCTGATACAAACAATCGTTACAACTTTCAACAATGGATCTCTTGGCTCTGGCATCGATGAAGAACGCAGCGAAATGCGATAAGTAGTGTGAATTGCAGAATTCAGTGAATCATCGAATCTTTGAACGCACATTGCGCCCCTTGGTATTCCATGGGGCATGCCTGTTCGAGCGTCATCTACACCCTCAAGCTCTGCTTGGTGTTGGGCGTCTGTCCCGCCTCTGCGCGTGGACTCGCCCCAAATTCATTGGCAGCGGCCTTGCCTCCTCTCGCGCAGCACAATTGCGTTTCTTGAGGTGGGTGGCCCGCGTCCACGAAGCAACATAACCGTCTTTGACCTCGGATCAG

>OTUpar2 *C. flexuosa*

TAGGTGAACCTGCGGAAGGATCATTATCCATCTCAAACCAGGTGCGGTCGCGGCCCCCTTAACCGGGTGGTTCGCGCCGCATTCCTGCATCCTTTTTTTACGAGCACCTTTCGTTCTCCTTCGGCGGGGCAACCTGCCGCTGGAACTTAACAAAACCTTTTTTTGCATCTAGCATTACCTGTTCTGATACAAACAATCGTTACAACTTTCAACAATGGATCTCTTGGCTCTGGCATCGATGAAGAACGCAGCGAAATGCGATAAGTAGTGTGAATTGCAGAATTCAGTGAATCATCGAATCTTTGAACGCACATTGCGCCCCTTGGTATTCCATGGGGCATGCCTGTTCGAGCGTCATCTACACCCTCAAGCTCTGCTTGGTGTTGGGCGTCTGTCCCGCCTTTGCGCGCGGACTCGCCCCAAATTCATTGGCAGCGGTCTTTGCCTCCTCTCGCGCAGCACATTTGCGTCTGCGAGGGGGCGTGGCCCGCGTCCACGAAGCAACATTACCGTCTTTGACCTCGGATCAGG

>OTUpar2 *C. ridleyi*

GGGTGCGGCCCCTTAACCGGGTGGTTCGCGCCGCGTTCCTGCATCCTTTTTTTACGAGCACCTTTTCGTTCTCCTTCGGCGGGGCAACCTGCCGCTGGAACTTAACAAAACCTTTTTTGCATCTAGCATTACCTGTTCTGATACAAACAATCGTTACAACTTTCAACAATGGATCTCTTGGCTCTGGCATCGATGAAGAACGCAGCGAAATGCGATAAGTAGTGTGAATTGCAGAATTCAGTGAATCATCGAATCTTTGAACGCACATTGCGCCCCTTGGTATTCCATGGGGCATGCCTGTTCGAGCGTCATCTACACCCTCAAGCTCTGCTTGGTGTTGGGCGTCTGTCCCGCCTCTGCGCGCGGACTCGCCCCAAATTCATTGGCAGCGGTCTTTGCCTCCTCTCGCGCAGCACATTTGCGTCTGCGAGGGGG
